# Diversity persists while function shifts: post-heatwave reorganization of reef fish communities in Baja California

**DOI:** 10.7717/peerj.21452

**Published:** 2026-07-08

**Authors:** Paulina Filz, Rodrigo Beas-Luna, Nicolas Loiseau, Julio Lorda, Jan Freiwald, Jeremie Bauer, Luis Malpica-Cruz

**Affiliations:** 1 Facultad de Ciencias Marinas, Universidad Autónoma de Baja California, Ensenada, Baja California, Mexico; 2 Laboratorio Nacional de Biología del Cambio Climático, SECIHTI, Mexico City, Mexico; 3 MARBEC, Univ Montpellier, CNRS, IFREMER, IRD, Montpellier, France; 4 Facultad de Ciencias, Universidad Autónoma de Baja California, Ensenada, Baja California, Mexico; 5 Tijuana River National Estuarine Research Reserve, Imperial Beach, CA, United States of America; 6 Reef Check Foundation, Santa Cruz, CA, United States of America; 7 Centro de Investigación Científica y Educación Superior de Ensenada, Marine Biotechnology Department, Ensenada, Baja California, Mexico; 8 Instituto de Investigaciones Oceanológica, Universidad Autónoma de Baja California, Ensenada, Baja California, Mexico; 9 ECOCIMATI AC, Ensenada, Baja California, Mexico

**Keywords:** Kelp forests, Functional resilience, Fish communities, Alpha diversity, Resilience

## Abstract

**Background:**

Marine heatwaves are increasing in frequency and intensity, driving persistent changes in kelp forests worldwide. Along the Pacific coast of Baja California, the 2014–2016 marine heatwave produced contrasting post-disturbance states, from persistent urchin barrens to understory-dominated reefs and declining canopy kelp. Kelp forests support reef-fish assemblages that sustain key ecosystem functions, yet how these functions track habitat reorganization after extreme warming remains unclear. Because no pre-heatwave baseline exists for the region, we examined post-disturbance trajectories from 2017 onward without assuming a common starting state across subregions.

**Methods:**

We analyzed underwater visual census data from 2017 to 2024 across 12 sites spanning three biogeographic subregions (North, Middle, South) along ∼600 km of coastline. We quantified temporal changes in algae, invertebrates, and reef fishes, and assessed fish assemblages using a trait-based framework built on nine ecological traits, including body size, trophic group, growth performance, and maximum fecundity. Taxonomic and functional α-diversity were estimated as biomass-weighted Hill numbers, and β-diversity as temporal β-decay (dissimilarity over time since 2017). Species and traits driving compositional change were identified *via* SIMPER. Fish secondary productivity and biomass turnover (P/B) were calculated using a temperature-dependent growth model with species-specific life-history parameters and local sea surface temperature. Temporal trends were evaluated with generalized linear mixed models.

**Results:**

Reefs followed divergent post-heatwave trajectories across subregions. Fish species richness and functional α-diversity remained stable everywhere, yet this stability masked contrasting regional dynamics in composition. In the North, assemblages showed minimal temporal change, consistent with a persistent urchin-barren configuration. In the Middle, functional β-decay increased over time, driven by shifts in trophic, growth, and body-size traits. The South showed the broadest reorganization, with significant increases in both taxonomic and functional β-decay and the strongest trait signal in maximum fecundity. Macroinvertivore biomass declined significantly in the North and showed a pronounced, though non-significant, decline in the South, with no compensatory increase in P/B among other functional groups. Our findings reveal a decoupling between biodiversity and ecosystem functioning in post-heatwave kelp-forest fish assemblages. Stable diversity metrics can conceal substantial reorganization in how assemblages produce and sustain biomass, and the biomass-dominant groups that underpin ecosystem processes are declining without compensation. Safeguarding these processes will require shifting from tracking diversity alone to directly monitoring and managing functional groups and biomass dynamics.

## Introduction

Intensifying marine heatwaves (MHWs) have become a major driver of abrupt ecological change in marine ecosystems worldwide (Wernberg et al., 2025). These events increasingly reshape species composition and community structure at unprecedented scales as climate change intensifies (Dahms & Killen, 2023). Along the west coast of North America, the 2014–2016 MHW caused major declines in kelp forests and pushed many areas into new post-heatwave states (Beas-Luna et al., 2020; McPherson et al., 2021; Tolimieri et al., 2023). Some sites lost their canopy entirely (Filbee-Dexter et al., 2020), others shifted to mixed understory communities (Bauer et al., 2025b), and some developed into urchin barrens (Rogers-Bennett & Catton, 2019). Kelp forests are globally important coastal habitats that support diverse fish communities, which in turn provide key functions such as biomass production, nutrient cycling, and predator control (Eger et al., 2023). Along the Pacific coast of Baja California, Mexico, this reorganization resulted in contrasting post-disturbance states: the North shifted toward persistent urchin barrens, the Middle developed into understory-dominated reefs, and the South retained canopy kelp despite warmer conditions (Beas-Luna et al., 2020). Subsequent work documented stable fish species richness but declining biomass in the North following the heatwave, while changes in benthic community composition continued across subregions in the years thereafter (Bauer et al., 2025b; Bauer et al., 2025a; Bosch et al., 2022). However, how these divergent habitat trajectories have shaped fish functional traits and productivity has not been assessed.

Species with different traits tend to respond differently to environmental change. In fish communities, traits such as body size, diet, and life-history characteristics determine both how species respond to environmental change and how they contribute to ecosystem processes (Mouillot et al., 2014). Kelp forest condition is typically assessed using indicators such as species richness, canopy cover, or total fish biomass. These metrics describe community structure at a given point in time but reveal little about the dynamic processes, such as secondary production and biomass turnover, that sustain ecosystem function over time. Incorporating these dynamic metrics is crucial for understanding whether habitat reorganization affects the functional stability of fish communities and for guiding management and conservation strategies (Nagelkerken et al., 2023).

Functional stability describes whether ecological processes, such as biomass production, can be sustained as communities reorganize (Valdivia, Aguilera & Broitman, 2021). It is promoted by biodiversity through complex biotic interactions and depends on how functional traits are distributed within a community, rather than on the number of species present (Stuart-Smith et al., 2013; Yan et al., 2023; Duffy et al., 2007; Fletcher Jr et al., 2025; Hillebrand et al., 2018). Functional diversity emerges from species identities, their relative biomass, and the degree to which their traits are complementary, redundant, or distinctive. Redundant species can buffer the loss of others with similar roles, while distinctive species may hold functions that cannot easily be replaced. These trait patterns influence how changes in the initial structure of a community, such as shifts in dominance, trait composition, and biomass among functional groups, affect the community as a whole and whether increases in some groups can compensate for declines in others (McLean et al., 2019). For example, biomass turnover reflects the rate at which biomass is regenerated and provides a dynamic indicator of ecosystem functioning and community resilience over time. Declines in kelp reduce habitat complexity and resource availability (Velasco-Charpentier et al., 2021), which can shift trait composition and trophic structure and thereby weaken the stabilizing mechanisms that sustain turnover (Mahaut et al., 2025). Assessing whether these functional processes persist under contrasting post-heatwave trajectories is therefore central to understanding the functional resilience of fish communities. Throughout this study, we operationally define functional resilience as the capacity of reef-fish assemblages to sustain biomass production, trophic transfer, and reproductive output despite community reorganization.

The northern Pacific coast of the Baja California peninsula provides a unique opportunity to investigate these dynamics. Spanning nearly 600 km, the region is divided into three biogeographic subregions (North, Middle, and South) that differ in oceanographic conditions and kelp forest alternative stable states. These contrasting trajectories offer a natural setting to evaluate how habitat reorganization shapes functional processes in fish communities. Because no pre-heatwave baseline data are available, we do not assume that these subregions originated from a common ecosystem state but instead interpret them as contrasting post-disturbance trajectories.

Here, we examine functional diversity and stability in reef-fish assemblages across these contrasting post-heatwave states. Drawing on standardized monitoring data from 2017 to 2024 across the subregions of the Baja California peninsula, we (1) document how algae, invertebrates, and fishes have reorganized over time and space, (2) assess changes in the taxonomic and functional diversity of reef-fish communities across different periods, and (3) analyze how fish size and biomass turnover vary over time among different functional groups and subregions.

We tested four hypotheses. First, we hypothesized that the biomass of fish functional groups would decline in subregions where habitats restructured most strongly following the marine heatwave. Second, we hypothesized that taxonomic species richness would remain stable across subregions, whereas functional diversity would show greater sensitivity to post-heatwave habitat trajectories. Third, we predicted that fish communities would diverge progressively from their initial post-heatwave composition in subregions where habitats changed most. Fourth, we predicted that habitat reorganization would reduce the functional stability of reef-fish communities, expressed as declining body size and biomass turnover over time.

By integrating biodiversity, trait, and ecosystem-function perspectives, our study offers a functional assessment of kelp-forest resilience and provides insights into the ability of reef-fish communities to maintain ecological functions as ocean warming increases.

## Materials and Methods

### Study area

**Figure 1:**
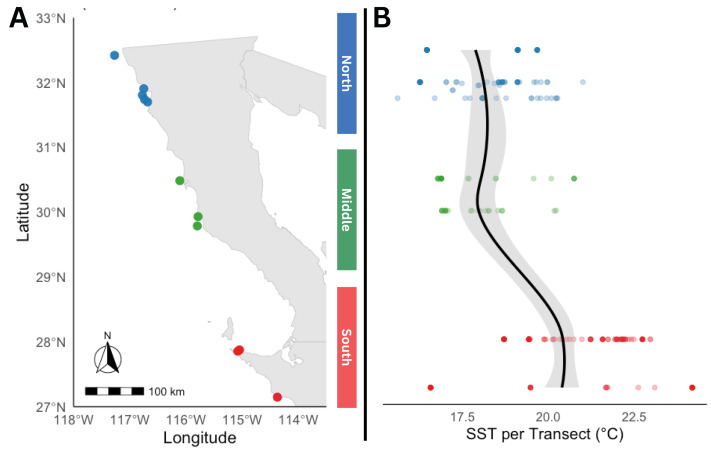
Study region, sampling design, and thermal gradient. (A) Map of the Pacific coast of the Baja California peninsula, Mexico, showing the spatial extent of the survey region (∼600 km). Monitoring sites are grouped into three latitudinal sections: North (blue), Middle (green), and South (red). (B) Mean annual sea surface temperature (SST, °C) per transect from 2017 to 2024, aligned by latitude (*y*-axis). SST data are derived from the NOAA Optimum Interpolation Sea Surface Temperature (OISST v2.1) dataset accessed through the NOAA ERDDAP server. The black line represents a LOESS-smoothed trend across all transects, while the grey shaded area denotes the 95% confidence interval (standard error band) around this trend.

The study was conducted along the northern Pacific coast of the Baja California peninsula, Mexico, covering approximately 600 km of coastline (Fig. 1A). Monitoring sites were organized into three biogeographic subregions that differ in latitude, oceanographic setting, and management context. The northern subregion comprised six sites, the Middle-subregion three sites, and the southern subregion three sites, monitored between 2017 and 2024. The northern subregion includes two federally managed, partially protected marine protected areas: Islas Coronados and Isla Todos Santos. The Middle-subregion includes two community-led no-take reserves, Isla San Jeronimo and Caracolera, which are managed by local fishing cooperatives. The southern subregion consists of the community-led no-take reserves at Piedra Blanca and Isla San Roque, also managed by local fishing communities, located near the southern distributional limit of kelp forests along the peninsula.

Ocean conditions in the study area are shaped by the interaction between the southward California Current, which transports cold, nutrient-rich water that sustains kelp productivity, and the northward Davidson Current, which brings warmer, nutrient-poor water during autumn and winter. This interaction creates a transition zone that generates a pronounced thermal gradient from north to south (Fig. 1B). Sea surface temperature data were obtained from the NOAA Optimum Interpolation Sea Surface Temperature version 2.1 (OISST v2.1) dataset, accessed through the NOAA ERDDAP server (https://coastwatch.pfeg.noaa.gov/erddap/). Daily SST data at 0.25° spatial resolution were extracted for the period 2017–2024 and matched to the exact sampling date and nearest grid cell of each transect using nearest-neighbor searches in R (*rerddap* and *FNN* packages, R Core Team, 2023). SST values were then averaged at the transect and site levels to represent local thermal conditions at the time of sampling. Figure 1B shows mean site-level SST aligned by latitude, illustrating the persistent north-to-south warming trend across the study area.

### Community composition and temporal trends

Monitoring sites were permanently established within each subregion and surveyed annually during the summer or autumn. In total, 12 sites were distributed across three biogeographic subregions. At each site, two to four replicate belt transects (30 × 2 m; 60 m^2^) were deployed within one or more subsites. Along each transect, divers using SCUBA recorded the abundance and size of macroalgae, mobile invertebrates, and reef fishes following standardized underwater visual census protocols (Beas-Luna et al., 2020; Freiwald et al., 2018). Fish total length was estimated visually to the nearest centimeter by trained divers, who calibrated their size estimates against reference objects of known length prior to the survey seasons. Algae and invertebrate data were expressed as density (individuals m^−2^). In contrast, fish counts were converted to biomass (g m^−2^) using species-specific length–weight relationships (Biomass = *a* × *Length*^b^) obtained from FishBase (https://fishbase.org/). Across all years, a total of 546 individual transects were surveyed between 2017 and 2024. Sampling effort varied among sites and years. A detailed breakdown of the number of transects per site, subregion, and year is provided in Table S1. To assess regional restructuring of kelp forest communities, we analyzed temporal changes in the density or biomass of functional groups of algae, invertebrates, and fishes. Algal groups included canopy kelp, subcanopy kelp, understory kelp, understory brown, understory green, understory red, corallines, and encrusting corallines. Because sea urchins, primarily *Mesocentrotus franciscanus* and *Strongylocentrotus purpuratus*, play a dominant and non-redundant role in shaping benthic community structure, they were treated as a separate functional group. The invertebrate herbivore group, therefore, includes all herbivores except urchins. Fish were assigned to five trophic functional groups: herbivores, planktivores, microinvertivores, macroinvertivores, and piscivores. The dominant species within each subregion and functional group are summarized in Table S3.

To account for spatial nesting and uneven survey effort, temporal trends in algal density, invertebrate abundance, and fish biomass were analyzed using generalized linear mixed-effects models (GLMMs) fitted to transect-level data. Models included Region (North, Middle, South), Year (centered and scaled), and their interaction with functional group identity (algal functional group, invertebrate functional group, fish functional group) as fixed effects. Site was included as a random intercept to account for repeated sampling and the hierarchical structure of the dataset (transects nested within sites across years). Response variables were log_1__+_-transformed prior to analysis to reduce heteroscedasticity and improve model fit. All models were fitted using the glmmTMB package in R (R Core Team, 2023). Temporal trends were inferred from the interaction between Year and Region, as well as from group-specific slopes, representing changes in density, abundance, and biomass over time across regions and functional groups. For visualization, model-based predictions were generated across the observed range of years while holding random effects at their population-level expectation. Predictions were back-transformed to the original scale, and 95% confidence intervals were calculated from the standard errors of the fitted values.

### Fish functional traits

We selected functional traits that capture key dimensions of species’ ecological strategies relevant to kelp-reef functioning and resilience, following the definition of Violle et al. (2007). Nine traits related to morphology, trophic ecology, and life history were compiled for 79 reef-fish species occurring across the study region. These included maximum total length (*MaxSizeTL*), length–weight parameters (*a*, *b*), trophic level, position in the water column, fishing vulnerability, dietary group, age at maturity (*tm*), and maximum fecundity (*FecundityMax*). Trait information was obtained primarily from FishBase (Froese & Pauly, 2026). When *tm* or *FecundityMax* values were unavailable for a given species, information was substituted from the closest related taxon within the same genus or family, prioritizing species with similar size and ecological characteristics. All substitutions, sources, and references are documented in Zenodo (Filz, 2025).

The nine selected traits capture key dimensions of species’ ecological strategies that shape both their responses to environmental change and their contributions to ecosystem functioning (Villéger et al., 2017; McLean et al., 2019; Stuart-Smith et al., 2013; Maire et al., 2021). Body size, fecundity, and age at maturity represent life-history trade-offs that influence population turnover and generation time, mediating resilience to disturbance and capacity for recovery (Brandl et al., 2019; Perry et al., 2005). Trophic level and dietary group describe species’ positions within food webs and their roles in energy transfer, nutrient cycling, and stability (Hicks et al., 2019; Tebbett, Bennett & Bellwood, 2024). Water-column position reflects habitat use and benthic–pelagic coupling (Griffiths et al., 2017), while fishing vulnerability indicates exposure to exploitation and potential loss of functional diversity (Dulvy, Sadovy & Reynolds, 2003).

### Taxonomic and functional diversity

To quantify temporal and regional changes in reef fish communities, we assessed both within-assemblage (*α*) and between-assemblage (*β*) diversity across two organizational dimensions—taxonomic and functional—and two sensitivity levels to species dominance (*q* = 0 and *q* = 1), yielding eight complementary metrics in total (Table 1). All metrics were calculated within a unified Hill numbers framework (Chao & Ricotta, 2019), which ensures mathematical comparability between taxonomic and functional components and across *q*-values. At *q* = 0, all species contribute equally regardless of biomass (richness-based); at *q* = 1, species contributions are weighted by their relative biomass (entropy-based). Taxonomic metrics quantify diversity based on species identity alone, while functional metrics additionally account for trait dissimilarity among species.

**Table 1:** Taxonomic and functional diversity metrics. Overview of the eight diversity metrics used in this study, organized by component (taxonomic/functional), scale (*α*/*β*), and sensitivity to species dominance (*q*-value).

**Metric**	**Component**	**Scale**	*q*-value	**Description**
Taxonomic richness	Taxonomic	*α*	*q* = 0	Species richness; all species weighted equally
Taxonomic entropy	Taxonomic	*α*	*q* = 1	Exponential of Shannon index; biomass-weighted
Functional richness	Functional	*α*	*q* = 0	Functional diversity; all species weighted equally
Functional entropy	Functional	*α*	*q* = 1	Functional diversity; biomass-weighted
Taxonomic *β*-decay (richness)	Taxonomic	*β*	*q* = 0	Dissimilarity from baseline; composition-based
Taxonomic *β*-decay (entropy)	Taxonomic	*β*	*q* = 1	Dissimilarity from baseline; biomass-weighted
Functional *β*-decay (richness)	Functional	*β*	*q* = 0	Functional dissimilarity from baseline; composition-based
Functional *β*-decay (entropy)	Functional	*β*	*q* = 1	Functional dissimilarity from baseline; biomass-weighted

#### *α*-diversity

Within-assemblage diversity was calculated at the transect level based on species biomass data. Taxonomic *α*-diversity was characterized by two metrics: taxonomic richness (*q* = 0), which assigns equal weight to all species, and taxonomic entropy (*q* = 1), corresponding to the exponential of the Shannon index:

(1) \begin{eqnarray*}TD={e}^{-\sum _{i=1}^{s}{p}_{i}\log \nolimits {p}_{i}}\end{eqnarray*}

where *S* is the total number of species and *p*_(*i*)_ is the relative biomass contribution of the *i*th species.

Functional *α*-diversity was estimated using the same Hill-based framework, where the parameter *τ* represents the mean functional distance among species within an assemblage. Functional diversity was calculated from pairwise trait distances as:

(2) \begin{eqnarray*}FD={ \left( \sum _{i=1}^{S}{p}_{i}{ \left( \sum _{j=1}^{S} \left[ 1-f \left( {d}_{ij}(\tau ) \right) \right] {p}_{j} \right) }^{q-1} \right) }^{1/(1-q)}.\end{eqnarray*}

For *q* = 0 (functional richness), all species contribute equally regardless of biomass:

(3) \begin{eqnarray*}FD=\sum _{i=1}^{S}\sum _{j=1}^{S}{ \left[ 1-f \left( {d}_{ij}(\tau ) \right) \right] }^{-1}.\end{eqnarray*}

For *q* = 1 (functional entropy), species contributions are weighted by their relative biomass:

(4) \begin{eqnarray*}FD={e}^{-\sum _{i=1}^{S}{p}_{i}\log \nolimits \sum _{j=1}^{S} \left[ 1-f \left( {d}_{ij}(\tau ) \right) \right] {p}_{j}}.\end{eqnarray*}

Functional distances (*d*_ij_) were calculated using the Gower metric applied to continuous and categorical traits describing species ecological roles, including maximum body size, growth parameters, trophic level, vertical position in the water column, fishing vulnerability, and reproductive traits. Only species with complete trait information were included in functional diversity calculations. All diversity metrics were calculated from transect-level community matrices, where species biomass was aggregated per transect and expressed as relative biomass for *q* = 1.

#### *β*-diversity

Between-assemblage diversity was calculated to quantify temporal changes in community composition and functional structure, using the same Hill numbers framework applied for *α*-diversity. Four *β*-diversity metrics were computed, corresponding to the taxonomic and functional components at *q* = 0 and *q* = 1. At *q* = 0, *β*-diversity reflects changes in species or trait composition irrespective of biomass; at *q* = 1, it reflects biomass-weighted differences in community structure. All indices range from 0 (identical communities) to 1 (completely distinct communities).

To quantify temporal change relative to initial community states, *β*-dissimilarity was expressed as *β*-decay: the dissimilarity between baseline communities (2017–2018) and all subsequent survey years within the same site and subregion. This approach captures the degree of cumulative community change relative to early post-heatwave conditions and yields a single, temporally ordered dissimilarity value per site and year.

#### Statistical analyses of *α*-diversity

Temporal trends in *α*-diversity were assessed separately for each of the four *α*-metrics (taxonomic richness, taxonomic entropy, functional richness, functional entropy) using generalized linear mixed models (GLMMs). For each metric, transect-level diversity values were used as the response variable. Fixed effects included subregion (North, Middle, South), Year (centered as a continuous variable), and their interaction, allowing subregion-specific temporal trends to be estimated. Site was included as a random intercept to account for non-independence arising from repeated sampling within sites. Year was treated exclusively as a continuous fixed-effect covariate and was not included as a random intercept, as it functions as a temporal predictor rather than a grouping factor. Models were fitted using a Gamma error distribution with a log link, appropriate for continuous, strictly positive response variables. Model predictions were generated at the population level and used for visualization of temporal dynamics.

#### Statistical analyses of *β*-diversity

Temporal trends in *β*-decay were assessed separately for each of the four *β*-metrics using GLMMs fitted to site-level dissimilarity values. Fixed effects included subregion, Year (centered), and their interaction. Site was included as a random intercept to account for spatial non-independence. Year was treated exclusively as a continuous fixed-effect covariate and was not included as a random intercept. Models were fitted using a beta distribution with a logit link, appropriate for bounded response variables between 0 and 1. Temporal trends were inferred from the interaction between Year and subregion, indicating changes in the magnitude of community dissimilarity over time relative to baseline conditions.

#### SIMPER analysis

To identify the components driving observed temporal dissimilarity, we complemented *β*-diversity analyses with a Similarity Percentage (SIMPER) decomposition (Clarke, 1993). SIMPER quantifies the contribution of individual community components to Bray–Curtis dissimilarity between pairs of years, allowing identification of the elements most responsible for temporal community reorganization. For each subregion (North, Middle, South), SIMPER contrasts were calculated between consecutive survey years. Where temporal gaps occurred (*e.g.*, missing data for the Middle subregion in 2021), comparisons were conducted between the nearest available years (2020–2022) to preserve temporal continuity.

SIMPER analyses were conducted at two complementary levels. First, taxonomic SIMPER was applied to species-level community matrices derived from transect-level biomass data to identify taxa contributing most to between-year changes in community composition. Second, functional SIMPER was applied to biomass-weighted community trait profiles to assess which trait dimensions contributed most to temporal changes in community functional structure. Trait profiles were calculated by weighting species-level trait values by their relative biomass within each transect. Prior to aggregation, continuous traits were rescaled to a common [0, 1] range using min–max normalization to ensure comparability across scales and to prevent traits with large numerical ranges from dominating dissimilarity estimates.

For each comparison, average SIMPER contributions and permutation-based *p*-values (999 permutations) were extracted to identify components whose contributions were unlikely to arise from random variation (*p* < 0.05). To evaluate whether temporal community change was driven by a small number of dominant contributors or by coordinated shifts across multiple components, we summarized significant SIMPER contributors across years and subregions. To visualize similarities in temporal contribution patterns, we constructed dendrograms separately for taxonomic and functional SIMPER results. For each subregion, SIMPER contributions of significant components were compiled into component-by-comparison matrices, which were then subjected to hierarchical clustering using Euclidean distance and complete linkage. This approach groups species or traits based on similarity in their temporal contribution patterns, rather than on ecological similarity in trait space. To assess whether patterns derived from SIMPER-based trait profiles were consistent with independent representations of functional structure, we compared community dissimilarity derived from trait profiles with dissimilarity calculated in a multivariate trait space. The latter was constructed using Gower distance on species-level traits followed by ordination (principal coordinates analysis) and projection of community composition into trait space. Correlation between both dissimilarity matrices was evaluated using a Mantel test with 999 permutations.

### Fish size

Fish size is a key functional attribute that strongly influences productivity, trophic interactions, and community resilience. Because it represents a fundamental dimension of functional structure, it was analyzed separately from the broader trait set. For each transect, biomass-weighted mean size was calculated for each functional group by weighting individual lengths by their estimated biomass. Temporal and spatial variation in fish size were analyzed using a GLMM. The response variable was log-transformed biomass-weighted mean size. Fixed effects included subregion (North, Middle, South), Year (scaled), functional group, and their interactions. To account for the hierarchical sampling design, Site and Sub-site nested within Site were included as random intercepts, with Year included as a fixed effect. Model fit was evaluated using marginal and conditional R^2^ values, representing the variance explained by fixed effects alone and by the full model including random effects, respectively. Temporal trends were estimated from the fitted model using marginal trends. Differences in fish size among subregions within functional groups were assessed using pairwise comparisons of estimated marginal means with Tukey adjustment for multiple testing. All analyses were conducted on the log scale.

To further resolve species-specific patterns underlying community-level trends, additional analyses were conducted at the species level. For each species within each subregion, biomass-weighted mean size was calculated by aggregating individual observations at the Site–Year level. Species-specific temporal trends were then analyzed using separate GLMMs, with log-transformed size as the response variable and Year (scaled) as a fixed effect. Site was included as a random intercept to account for repeated observations and spatial structure. Analyses were restricted to species with sufficient observations (*n* > 20 per subregion) to ensure robust model estimation. For each model, the effect of Year was extracted, and effect sizes were back transformed to represent annual percentage change in body size. Model performance was assessed using marginal R^2^ values.

### Secondary productivity and biomass turnover

We quantified fish secondary productivity using the rfishprod R package (Morais & Bellwood, 2020, available at: https://github.com/renatoamorais/rfishprod). This package applies a temperature-dependent growth framework to estimate somatic production from underwater visual census data. The method links species identity, abundance, and body length of individual fishes to species-specific growth and mortality parameters, yielding net secondary productivity expressed as kilograms per hectare per year (kg ha^−1^ yr^−1^). Species–specific length–weight coefficients and life history traits, including growth and mortality parameters, were primarily obtained from FishBase (Froese & Pauly, 2026) and complemented with predictive models (Gislason et al., 2010; Morais & Bellwood, 2018; Thorson, Jannot & Somers, 2017). Individual body mass (g) was derived from field-measured lengths using species–specific length–weight relationships, or from closely related taxa when unavailable.

Growth trajectories were modeled using the von Bertalanffy growth function (VBGF), which describes the relationship between size and age for each species. The growth coefficient (Kmax) and asymptotic length (L∞) were used to predict annual increments in body mass, scaled by the local thermal environment. Temperature dependence was explicitly incorporated *via* the mean annual sea surface temperature (SST) for each transect, derived from the NOAA Optimum Interpolation Sea Surface Temperature version 2.1 (OISST v2.1) dataset accessed through the NOAA ERDDAP server (https://coastwatch.pfeg.noaa.gov/erddap/). Expected biomass loss due to natural mortality was calculated deterministically and subtracted from total somatic growth to estimate annual fish productivity. Individual-level estimates were then aggregated across species and functional groups to obtain total secondary productivity per transect, representing the overall rate of biomass production within reef fish communities. Fish biomass turnover (P/B ×100%) was calculated as the ratio of total annual production (P) to standing biomass (B), providing the estimated rate at which community biomass is replaced over time. Turnover values were log-transformed [log(x + 1 × 10^−6^)] to improve normality and variance homogeneity.

Temporal and regional variation in turnover were analyzed using generalized linear mixed-effects models (GLMM) implemented in glmmTMB with a Gaussian error distribution. Fixed effects included scaled Year, subregion, functional group, and their interactions, while Site and Sub-site were included as nested random intercepts to account for the hierarchical sampling design. Although this random-effect structure resulted in a singular fit with random-effect variance estimated at zero, the GLMM was retained to respect the nested sampling structure. Model comparison confirmed that a fixed-effects-only specification yielded a virtually identical fit (ΔAIC < 1; GLM: AIC = 8,752.08, GLMM: AIC = 8,752.49), indicating that fixed-effect estimates and confidence intervals are not meaningfully affected by the inclusion of the random effect. Model performance was quantified using marginal R^2^ (variance explained by fixed effects) calculated with the performance package. Conditional R^2^ was not estimated due to negligible variance attributed to the random effect. Fixed effect estimates and confidence intervals were obtained using the broom.mixed R package (R Core Team, 2023). Temporal trends were quantified using estimated marginal trends and expressed as percentage change per year following back-transformation.

### Field permits

Animal welfare considerations followed relevant international guidance for non-invasive field research on wild fishes, including established ethical standards for underwater visual census surveys. Fieldwork was conducted under permits issued according to the administrative requirements of each surveyed site, as detailed below: Mexican Fisheries and Aquaculture Commission (CONAPESCA: PPD/DGOPA-001/18), and from the Mexican Natural Protected Areas Commission (CONANP: F00.DRPBCPN.RBIPPBC.-216/2021; F00.DRPBCPN.02540/2025).

### Use of artificial intelligence tools

Artificial intelligence tools were used to support parts of the analytical workflow and manuscript preparation. Specifically, ChatGPT (OpenAI; https://chatgpt.com/) and Claude (Anthropic; https://claude.ai/) were used to assist with drafting and refining R code, improving code readability, and translating and editing text into English. All analyses were conducted, checked, and interpreted by the authors, and all scientific content remains the responsibility of the authors.

## Results

### Regional restructuring of kelp forest communities

**Figure 2:**
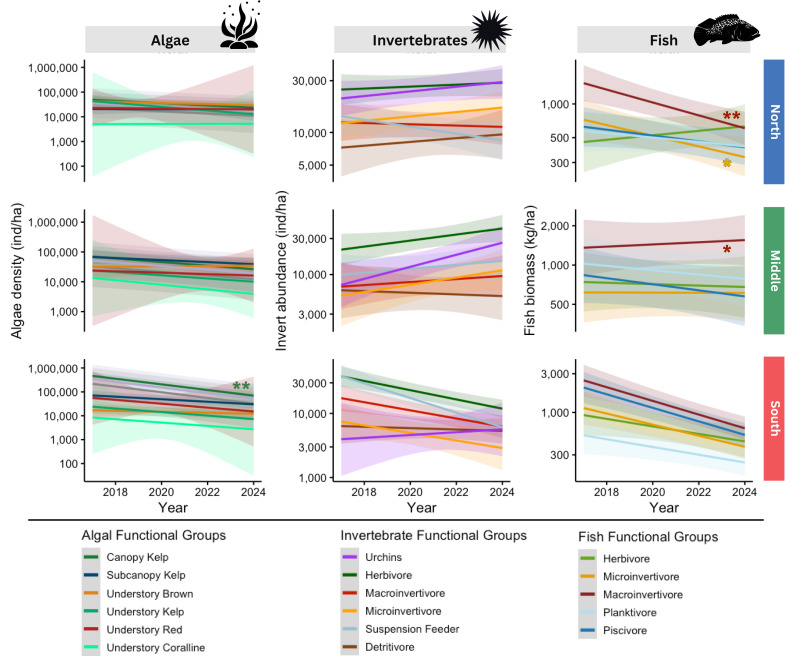
Temporal trends in benthic and fish community structure across Baja California. Temporal dynamics of algal density, invertebrate abundance, and fish biomass across three regions (North, Middle, South) from 2017 to 2024. Lines represent modelled trends derived from generalized linear mixed-effects models (GLMMs) fitted to transect-level data, with Year as a fixed effect and Site included as a random intercept to account for spatial nesting. Shaded ribbons indicate 95% confidence intervals based on population-level predictions. Response variables were log_1+_-transformed for model fitting and back-transformed for visualization. Colors denote functional groups within each trophic level (see legend). *Y*-axes are displayed on a log_1+_ scale to facilitate comparison across orders of magnitude. Created in: Canva.

To assess how the kelp-reef ecosystem reorganized after the 2014–2016 marine heatwave, we quantified temporal trajectories in algae, invertebrates, and fish from 2017 to 2024 across the North, Middle, and South subregions of Baja California (Fig. 2). Full model outputs, including fixed-effect estimates, standard errors, and interaction terms for algae density, invertebrate abundance, and fish biomass, are provided in Table S2. Algae density differed markedly across reef subregions and declined over time. Overall density was significantly higher in the South than in the North (*β* = 1.64 ± 0.23 SE, *p* < 0.001), yet canopy kelp (the dominant functional group in the South) showed a significant negative temporal trend in this subregion (*β* = −0.39 ± 0.13 SE, *p* = 0.003). Subcanopy and understory kelp densities were both substantially lower in the South relative to the North (*β* = −0.83 ± 0.19 SE, *p* < 0.001 and *β* = −2.21 ± 0.27 SE, *p* < 0.001, respectively), while subcanopy kelp was elevated in the Middle subregion (*β* = 0.69 ±0.25 SE, *p* = 0.006). Understory brown algae showed a negative South effect (*β* = −2.54 ± 0.24 SE, *p* < 0.001).

Invertebrate communities were dominated by herbivores and urchins, with both groups showing significantly higher abundance than all other functional groups (*β* = 1.16 ± 0.15 SE, *p* < 0.001 and *β* = 1.08 ± 0.15 SE, *p* < 0.001, respectively). Urchin abundance was markedly elevated in the North and declined southward, with significantly lower abundances recorded in the South (*β* = −1.26 ±  0.35 SE, *p* < 0.001). Microinvertivore abundance was similarly reduced in the South (*β* = −0.83 ± 0.29 SE, *p* = 0.004), whereas suspension feeders showed the opposite pattern, being significantly more abundant in the South compared to the North (*β* = 0.64 ± 0.23 SE, *p* = 0.006). No significant temporal trends were detected for any invertebrate functional group.

Fish biomass varied by functional group and showed notable temporal and spatial dynamics. Macroinvertivores had significantly higher biomass than herbivores across all subregions (*β* = 0.37 ± 0.15 SE, *p* = 0.012), yet their biomass declined significantly over time in the North (*β* = −0.37 ± 0.14 SE, *p* = 0.008). This negative trend was attenuated in the Middle subregion, where a significant positive three-way interaction indicated a diverging temporal trajectory for macroinvertivores (*β* = 0.44 ±  0.19 SE, *p* = 0.020). Microinvertivore biomass also declined over time in the North (*β* = −0.32 ± 0.15 SE, *p* = 0.030). Piscivore biomass was significantly higher in the South relative to the North (*β* = 0.57 ± 0.20 SE, *p* = 0.005), while no significant temporal trends were detected for piscivores or planktivores in any subregion.

### Fish taxonomic and functional diversity

#### *α*-diversity

**Figure 3:**
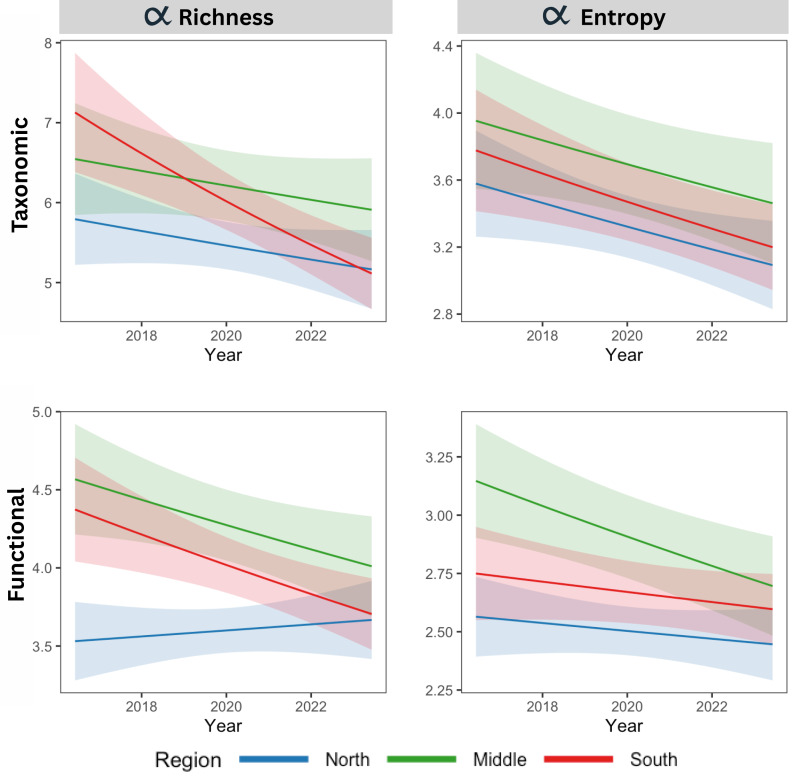
Taxonomic and functional *α*-diversity of reef fish communities over time. Temporal trends in taxonomic and functional *α*-diversity across biogeographic subregions (North = blue, Middle = green, South = red). Diversity metrics were calculated using Hill numbers, where *q* = 0 represents species richness and *q* = 1 represents Shannon entropy weighted by relative biomass. Functional diversity was derived from multidimensional trait distances (Gower metric) based on species-level traits describing size, growth, trophic level, habitat position, reproduction, and fishing vulnerability. Values were calculated at the transect level. Lines represent modelled trends over time, and shaded areas indicate uncertainty around predictions. Trends were estimated using generalized linear mixed models (GLMMs) with subregion, Year, and their interaction as fixed effects, and Site included as random intercept.

Taxonomic *α*-diversity did not exhibit significant temporal trends across any subregion (Fig. 3). Species richness (q_0_) fluctuated interannually within subregions but showed no directional change over time. Similarly, taxonomic entropy (q_1_) remained stable, with no significant effects of Year or Year × subregion interactions (all *p* > 0.05; marginal *R*^2^ = 0.024), indicating that variation in taxonomic *α*-diversity was not explained by temporal dynamics. Full model results, including temporal trends, are provided in Table S4. Sample sizes (n) and mean values per year and subregion are presented in Table S5.

Functional *α*-diversity showed significant differences among subregions. Functional richness (FDq_0_) was higher in the Middle (*β* = 0.160, *p* = 0.0058) and South (*β* = 0.096, *p* = 0.042) subregions compared to the North subregion. However, no temporal trends were detected (Year: *p* = 0.74), and interactions between Year and subregion were not significant (all *p* > 0.05), suggesting that these regional differences remained stable over time (marginal *R*^2^ = 0.049). Functional entropy (FDq_1_) was slightly higher in the Middle compared to the North (*β* = 0.142, *p* = 0.049), while differences between South and North were not significant (*p* = 0.30). As with all other *α*-diversity metrics, no temporal trends were observed (Year: *p* = 0.64), and interaction terms were not significant (all *p* > 0.05; marginal *R*^2^ = 0.035).

#### *β*-diversity

**Figure 4:**
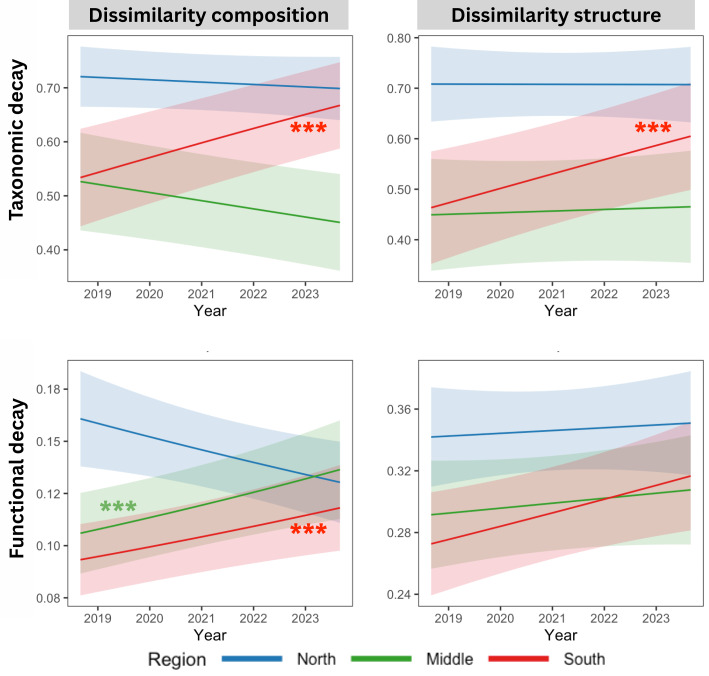
Temporal dissimilarity of reef fish communities. Temporal trends in mean taxonomic and functional *β*-decay (±SE) across years and biogeographic subregions (North = blue, Middle = green, South = red). Decay values represent Jaccard-based dissimilarity relative to the 2017–2018 baseline. The first row shows taxonomic *β*-decay in species composition (*q* = 0) and structure (*q* = 1), and the second row shows functional *β*-decay in composition (*q* = 0) and structure (*q* = 1). Trends were estimated using generalized linear mixed models (GLMMs) fitted to site-level decay values, with subregion, Year, and their interaction as fixed effects, and Site included as a random intercept to account for spatial non-independence. Models were fitted using a beta distribution with a logit link. Shaded areas represent 95% confidence intervals of model predictions. Asterisks indicate significant temporal trends based on the interaction between Year and subregion (*p* < 0.05).

Taxonomic *β*-decay describes how strongly fish communities diverge from their initial state over time (Fig. 4). For taxonomic composition (*q* = 0), decay was lower in the Middle compared to the North (*β* = −0.954 ± 0.414 SE, *p* = 0.021), indicating smaller changes in species composition in the Middle subregion relative to the starting community. No difference was detected between the South and North (*β* = −0.434 ± 0.413 SE, *p* = 0.29). There was no overall temporal trend across all subregions (Year: *β* = −0.021 ± 0.047 SE, *p* = 0.65), and no interaction between Year and subregion in the Middle (*β* = −0.039 ± 0.024 SE, *p* = 0.11). In contrast, decay increased over time in the South, as indicated by a positive interaction between Year and subregion (*β* = 0.134 ± 0.026 SE, *p* < 0.001). Fixed effects explained around a quarter of the variation in taxonomic decay (marginal *R*^2^ = 0.27). A similar pattern was observed for taxonomic structure (*q* = 1), which accounts for species biomass. Decay was lower in the Middle compared to the North (*β* = −1.051 ±  0.470 SE, *p* = 0.025), while no difference was found between the South and North (*β* = −0.705 ± 0.469 SE, *p* = 0.13). No overall temporal trend was detected (Year: *β* = −0.001 ± 0.079 SE, *p* = 0.99), and no interaction was found for the Middle (*β* = 0.014 ±  0.028 SE, *p* = 0.62). In the South, decay increased over time (*β* = 0.116 ± 0.029 SE, *p* < 0.001). Fixed effects explained a part of the variance (marginal *R*^2^ = 0.228).

For functional *β*-decay (*q* = 0), which reflects changes in functional trait composition rather than species identity, no regional differences were detected (all *p* > 0.05), and no overall temporal trend was observed (Year: *β* = −0.049 ± 0.038 SE, *p* = 0.20). However, decay increased over time in both the Middle (*β* = 0.107 ± 0.030 SE, *p* < 0.001) and the South (*β* = 0.102 ± 0.030 SE, *p* < 0.001). Fixed effects explained a small proportion of the variance (marginal *R*^2^ = 0.017). Functional structure (*q* = 1) showed no significant regional differences (all *p* > 0.05), no temporal trend (Year: *β* = 0.008 ± 0.036 SE, *p* = 0.83), and no significant interactions between Year and subregion (all *p* > 0.05), with fixed effects explaining a small proportion of the variance (marginal *R*^2^ = 0.023). Full model results are provided in Table S4, and sample sizes and descriptive statistics per year and subregion are reported in Table S6.

#### SIMPER analysis

To identify the species and traits driving temporal *β*-dissimilarity, we applied SIMPER analysis to consecutive year-pair comparisons within each subregion (Tables S13 and S14, Fig. S2). A combined overview of taxonomic and functional SIMPER contributions across all year-pair comparisons and subregions is provided in Table S7. Across subregions, the species with the highest mean contributions to Bray–Curtis dissimilarity were not necessarily the ones that contributed significantly from year to year. For example, in the North and Middle, several high-ranking species (*e.g.*, *Bodianus pulcher*, *Rhacochilus vacca*) ranked near the top by mean SIMPER contribution but did not reach significance (*p* < 0.05) in any single consecutive-year comparison. Statistically consistent signals, defined as species significant in at least one year-pair, came instead from a smaller subset of taxa (Table S13). Taxonomic turnover in the North was driven by infrequent, non-repeated contributions from resident species, primarily *Paralabrax clathratus* and *Chromis punctipinnis* (each significant in two comparisons), consistent with interannual fluctuation within a compositionally stable, low-diversity assemblage. In the Middle, *Embiotoca jacksoni* was the only species with repeated significant contributions (three comparisons), despite not ranking among the five highest mean contributors, pointing to episodic dominance shifts rather than gradual directional change. The South showed the broadest and most consistent taxonomic signal, with *P. clathratus* and *Anisotremus davidsonii* each significant in three comparisons and five additional species significant in two or more, indicating widespread and repeated community reorganization.

Functional patterns reinforced these regional differences (Table S14): in the North, functional change was primarily associated with shifts in fishing vulnerability and growth parameters, with fishing vulnerability contributing significantly in four year-pair comparisons and the growth parameter b in two, while reproductive and trophic traits showed no significant contributions, indicating that despite ongoing taxonomic turnover, functional reorganization was concentrated in vulnerability and growth dimensions. In the Middle, change was driven primarily by trophic position and growth-related traits, with trophic level significant in two comparisons and the growth coefficient a, fishing vulnerability, and maximum body size each significant once, rather than by reproductive traits such as maximum fecundity. In the South, functional reorganization was multidimensional, with maximum fecundity showing the strongest and most consistent signal (four significant comparisons), alongside coordinated shifts in trophic position, age at maturity, and fishing vulnerability. Hierarchical clustering of significant SIMPER contributors showed that taxonomic contributions in the South were more structured and temporally coherent than in the North and Middle (Fig. S2A), while functional clustering revealed coordinated shifts among multiple traits only in the South, with little to no consistent structure in the other subregions (Fig. S2B).

The validity of SIMPER-based functional trait profiles was supported by a Mantel test comparing community dissimilarity derived from trait profiles with dissimilarity calculated in an independent multivariate trait space constructed using Gower distance and principal coordinates analysis. The two dissimilarity matrices were significantly correlated (Mantel *r* = 0.014, *p* = 0.001), confirming that despite the low correlation coefficient, expected given the dimensionality reduction and differences in trait representation between approaches, the trait profiles captured a consistent and non-random signal of functional community structure. Our results indicate that community reorganization in the South was driven by a coordinated set of taxa and traits acting simultaneously, whereas change in the North was functionally structured around vulnerability and growth dimensions, and change in the Middle was more episodic and trophically mediated. A more detailed description of all SIMPER results is provided in the Supplementary Materials.

### Fish size and biomass turnover

**Figure 5:**
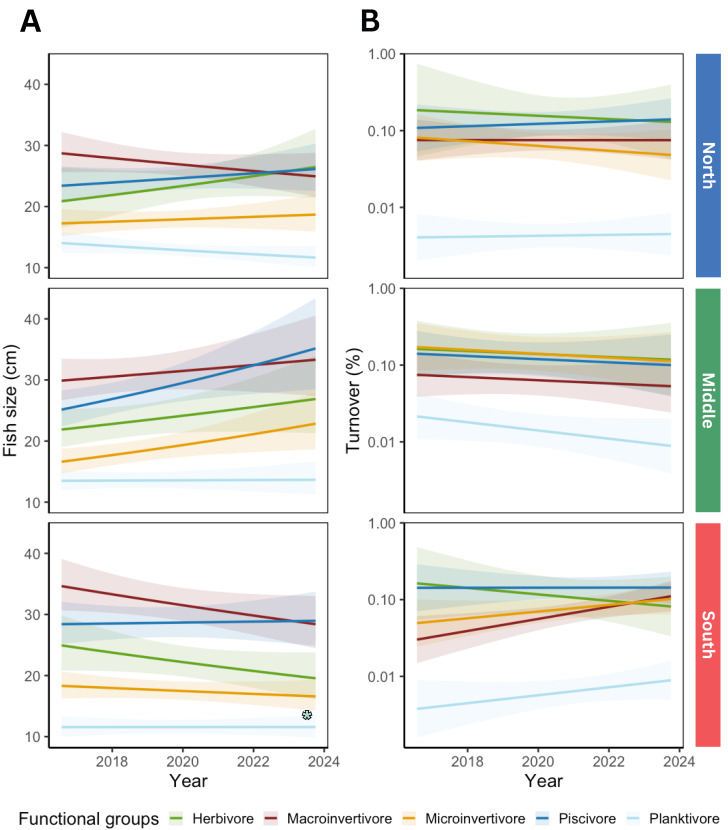
Temporal trends in fish size and biomass turnover across Baja California. (A) Biomass-weighted mean body size (cm) of reef fishes from 2017–2024 across three subregions (North, Middle, South), estimated at the transect level and analyzed using generalized linear mixed-effects models (GLMMs). (B) Biomass turnover (P/B × 100%) over the same period, calculated at the transect level and analyzed using GLMMs. In both panels, lines represent model predictions and shaded ribbons indicate 95% confidence intervals. Models included year, subregion, functional group, and their interactions as fixed effects, with site included as a random intercept. Colors denote functional groups: herbivores (green), macroinvertivores (brown), microinvertivores (orange), piscivores (blue) and planktivores (light blue). Asterisks indicate significant temporal trends (*P* < 0.05).

Biomass-weighted fish size showed no significant overall differences among subregions and no consistent temporal trend across all groups (all *p* > 0.05). Fixed effects explained a substantial proportion of the variance in fish size (marginal *R*^2^ = 0.46), with a slightly higher proportion when including random effects (conditional *R*^2^ = 0.50). Fish size differed among functional groups, whereby macroinvertivores were larger on average (Fig. 5A; Table S9). Microinvertivores and planktivores were smaller relative to the reference group (all *p* < 0.05). Piscivores did not differ significantly. Regional differences were detected within specific functional groups. In the South, macroinvertivores and piscivores were larger compared to North (both *p* < 0.01), while no significant regional differences were found for microinvertivores or planktivores. Pairwise comparisons confirmed these patterns, showing differences between North and both Middle and South for macroinvertivores, and between North and Middle for piscivores, while other contrasts were not significant. Planktivores showed a significant decline in size over time (*p* < 0.05), whereas no significant temporal trends were detected for the other groups. A significant interaction indicated that temporal trends in planktivore size differed in the South relative to North (*p* < 0.05). Full model results are provided in Table S9, and results of pairwise comparisons are presented in Table S10.

Species-specific analyses were restricted to species with sufficient observations (*n* > 20 per subregion) and revealed that temporal changes in body size were limited to a small subset of taxa. Most species showed no significant temporal trends within subregions, with low effect sizes and negligible variance explained by year (Table S11). Significant changes were detected in only two species. In the southern subregion, *Halichoeres semicinctus* exhibited a significant decline in body size (−9.1% per year, *p* = 0.030; marginal *R*^2^ = 0.19), representing the strongest negative trend observed across all species. In contrast, in the northern region, *Paralabrax clathratus* showed a significant increase in body size (+10.6% per year, *p* = 0.024; marginal *R*^2^ = 0.17). No significant trends were detected for these species in other subregions. Across the remaining species, including *Chromis punctipinnis*, *Hypsypops rubicundus*, *Oxyjulis californica*, and *Bodianus pulcher*, temporal trends were weak and non-significant despite occasional directional shifts, with annual changes generally below ±6%. A visual overview of species-specific trends is presented in Fig. S1 and the corresponding statistical results are provided in Table S11.

Biomass turnover did not exhibit significant temporal trends across the study period (Year: estimate = −0.116 ±  0.387 SE, *p* = 0.765; Fig. 5B). Similarly, no significant interactions between Year and subregion or functional group were detected (all *p* >  0.28), indicating that turnover remained temporally stable across subregions and functional groups. In contrast, differences in turnover were observed among functional groups. Compared to herbivores (reference group), turnover was significantly lower in macroinvertivores (estimate = −0.704 ± 0.338 SE, *p* = 0.037), microinvertivores (estimate = −0.912 ± 0.360 SE, *p* = 0.011), and planktivores (estimate = −3.568 ± 0.347 SE, *p* <  0.001), while piscivores did not differ significantly from herbivores (estimate = −0.198 ± 0.347 SE, *p* = 0.568). Most interaction terms between functional group, subregion, and Year were not significant (all *p* >  0.28), indicating that differences in turnover among functional groups did not vary across subregions or over time. A single exception was detected for planktivores in the Middle subregion, which showed higher turnover relative to the North (subregion × functional group interaction: estimate = 1.245 ±  0.544 SE, *p* = 0.022). Fixed effects explained a moderate proportion of variance in turnover (marginal *R*^2^ = 0.205), while random-effect variance for Site was negligible, resulting in singular model fits and preventing estimation of conditional R^2^. Full model results are provided in Table S12.

## Discussion

Our study shows that reef-fish communities can maintain stable species richness and trait diversity while undergoing substantial changes in biomass distribution and functional processes as kelp forests reorganize following the 2014–2016 marine heatwave. Across the Baja California peninsula, this reorganization resulted in contrasting habitat trajectories, ranging from persistent urchin barrens with little to no canopy kelp in the North, to gradual understory-dominated reefs in the Middle, and continued, albeit declining, canopy kelp in the South. These patterns occur along a latitudinal temperature gradient, with warmer conditions toward the South, where kelp forests approach their distributional limit. Despite these divergent post-heatwave states and subregion-specific thermal conditions, we detected no significant losses in taxonomic richness or functional *α*-diversity within subregions. Instead, community change unfolded as a gradual reorganization over time, with fish communities in the South becoming increasingly dissimilar from one year to the next in both taxonomic and functional composition, as well as in taxonomic structure. Within this broader pattern, macroinvertivore biomass declined significantly in the North and also decreased in the South, although the latter trend was not statistically significant.

The contrasting patterns of functional reorganization among subregions suggest that similar *α*-diversity can mask fundamentally different ecological dynamics, with regional differences concerning how communities were reorganized rather than how many species or functional roles were represented. In the North, neither taxonomic nor functional *β*-decay increased significantly through time relative to the 2017–2018 baseline, so the fish community remained compositionally and functionally close to its early post-heatwave state. The only detectable functional change in the North in year-to-year SIMPER comparisons was concentrated in fishing vulnerability and growth parameters, with no significant contributions from reproductive or trophic traits. This combination of stable *β*-decay and narrow, non-directional trait turnover is consistent with a fish community locked into a persistent urchin-barren configuration (Eger et al., 2024; Filbee-Dexter & Scheibling, 2014), where limited habitat complexity constrains colonization and maintains a relatively fixed species pool (Sanabria-Fernández, Génin & Dakos, 2024; Olán-González et al., 2023).

By contrast, the Middle and South subregions diverged from their early post-heatwave state, but in different ways. In the Middle, functional *β*-decay (*q* = 0) increased significantly through time while taxonomic *β*-decay did not, indicating that the community changed more in the functional traits it carried than in species identity. SIMPER analyses further identified trophic level and growth-related traits (growth coefficient a, maximum body size, fishing vulnerability) as the main contributors in the Middle, rather than reproductive traits. The South showed the broadest reorganization, with significant temporal increases in both taxonomic *β*-decay (*q* = 0 and *q* = 1) and functional *β*-decay (*q* = 0), whereas biomass-weighted functional structure (*q* = 1) remained stable. SIMPER in the South revealed coordinated shifts across multiple traits, with maximum fecundity producing the strongest and most consistent signal alongside trophic position, age at maturity, and fishing vulnerability, rather than change along a single dominant axis. Such coordinated, multi-trait reorganization indicates a deeper reconfiguration of how biomass is produced and transferred through the food web, rather than a simple reshuffling of biomass dominance (Gravel, Albouy & Thuiller, 2016).

In kelp forests specifically, loss of canopy and the shift to urchin barrens or understory-dominated reefs reduces three-dimensional structure and prey availability, which can modify the size structure and trophic organization of associated fish assemblages (Graham, 2004; Pérez-Matus et al., 2017). More generally, traits related to body size, trophic position, and life-history strategies can shift in response to habitat change even when species richness remains stable (Hadj-Hammou et al., 2024). This suggests that the observed functional changes in the Middle and South are consistent with habitat-driven reorganization, in which environmental change reshapes how functional traits are expressed within fish assemblages rather than altering species composition alone. In our system, this implies that resilience of fish communities does not mean a return to former conditions, but the ability to sustain key functions, such as biomass production (Hidalgo et al., 2022), trophic transfer (Calderon-Aguilera et al., 2022), and reproductive output (Le Bris et al., 2015), as kelp forests decline.

Interpreting these community-level patterns requires considering the biology of the dominant taxa driving interannual change. Three species recurred as major contributors across subregions: the California sheephead (*Bodianus pulcher*), a large wrasse that preys on benthic invertebrates including sea urchins and that can exert top-down control on urchin populations in southern California kelp forests (Cowen, 1983; Hamilton, Newsome & Caselle, 2014); the Garibaldi (*Hypsypops rubicundus*), a site-attached, non-fished damselfish whose abundance largely tracks habitat condition (Fuentes Calderon et al., 2024); and the kelp bass (*Paralabrax clathratus*), a generalist predator with a flexible, kelp-associated diet (Mendoza-Carranza & Rosales-Casian, 2022). Because *B. pulcher* is one of the few fishes in this system known to consume sea urchins, its dynamics are particularly relevant to whether fish-mediated top-down pressure could help constrain urchin dominance in the North (Hamilton, Newsome & Caselle, 2014).

However, our data provide little evidence that such fish-mediated top-down pressure is currently operating in the North. On the contrary, macroinvertivore biomass, the functional group encompassing the main known urchin predators in these assemblages, declined significantly over time in the North (*β* = −0.37 ± 0.14 SE, *p* = 0.008; Fig. 3), further weakening the potential for predator-driven urchin control (Steneck et al., 2002; Hamilton & Caselle, 2015). Complementary linear trend analyses confirmed that the biomass of these three species showed no directional change through time in any subregion (Table S8), indicating that the overall decline is a community-level rather than a species-specific signal. Interannual variation was driven consistently by this small set of resident species within an already simplified assemblage, which showed the lowest functional *α*-richness and *α*-entropy among subregions. Although *B. pulcher* produced the largest mean contribution to Bray–Curtis dissimilarity across consecutive year-pair comparisons in each subregion, none of these contributions were significant. Moreover, effective urchin predation by *B. pulcher* is strongly size-dependent, since only large individuals can successfully consume adult sea urchins (Cowen, 1983; Hamilton et al., 2007; Selden et al., 2017). Therefore, we suggest that the large SIMPER contribution of *B. pulcher* reflects short-term, non-directional fluctuations in the biomass of an already small population, rather than sustained directional changes capable of shifting the system away from urchin dominance, although our data cannot test this mechanism directly.

In the South, the decline in macroinvertivore biomass was driven primarily by the replacement of larger-bodied species with smaller taxa rather than by uniform size reductions within species. Only the Rock Wrasse (*Halichoeres semicinctus*) showed a significant decline in mean body size (−9.1% per year, *p* = 0.030), the strongest negative size trend observed in this study. As a benthic carnivore preying predominantly on small invertebrates, a shift toward smaller size classes in *H. semicinctus* can modify prey selectivity and top-down control on benthic invertebrate assemblages, even where biomass change is little affected (Keppeler, Montaña & Winemiller, 2020). Importantly, this size-related shift did not occur in isolation: because fecundity scales nonlinearly with body size, the replacement of larger-bodied macroinvertivores by smaller taxa (together with the fecundity-dominated SIMPER signal described above) translates into disproportionate reductions in reproductive output at the assemblage level (Hadj-Hammou et al., 2024).

Although the fish community structure changed substantially, we found no evidence of compensation through higher biomass turnover. As noted above, macroinvertivore biomass declined in the North and, less clearly, in the South, while mean body size remained stable across every trophic group. Under metabolic scaling theory (Brown et al., 2004; Anderson-Teixeira et al., 2009), smaller, fast-growing taxa are expected to exhibit higher turnover rates than larger, slow-growing species. Studies from coral reef systems suggests that this size-structured dynamic can act as a buffering mechanism, whereby increases in the turnover of small-bodied groups, such as herbivores, microinvertivores, and planktivores, compensate for declines in larger, slow-turnover taxa (Morais & Bellwood, 2020; Seguin et al., 2023). In our system, however, macroinvertivore turnover remained unchanged despite the biomass loss, and no other functional group scaled up biomass production to compensate. This decline is particularly striking because species with slower life histories are generally expected to benefit from warming through faster growth and earlier maturation, while fast-life-history species face rising metabolic demands and reduced population growth (Wang et al., 2020). That macroinvertivores declined despite this thermal expectation indicates that temperature alone cannot account for the losses, pointing instead to fishing pressure and habitat reorganization following kelp decline; at the same time, the groups best placed to offset the loss are themselves thermally constrained, further limiting compensation. In other regions such as the Northwest Pacific, fast, early maturing species are projected to track favorable conditions poleward and restructure communities along the way (Osland et al., 2021), but no comparable redistribution was observed along Baja California’s persistent north-to-south warming gradient. The resulting lack of compensatory buffering (Morais et al., 2023) suggests that macroinvertivore biomass here may not be sustained over time, reducing the system’s capacity to recover lost functional integrity.

Habitat reorganization, ocean warming, and fishing pressure all co-occur across subregions, and their individual effects cannot be fully separated with the available data. The SIMPER results suggest that fishing had its strongest signal in the North, where community shifts were concentrated in species with high fishing vulnerability, but a weaker signal in the South, where changes were driven mainly by life-history traits such as fecundity and age at maturity. Across the region, however, fishing is unlikely to be the main driver: size distributions showed no truncation typical of heavily fished assemblages (Tu, Chen & Hsieh, 2018), heavily and lightly fished functional groups followed similar temporal trajectories, and life-history traits such as growth, maturation, and lifespan respond more strongly to environmental conditions than to fishing pressure (Beukhof et al., 2019). These patterns are consistent with habitat change being a major driver of functional reorganization, while fishing might act as a secondary, spatially variable pressure.

Regardless of the relative weight of each driver, our findings underline the need for management actions that jointly address fishing pressure and climate-driven habitat change to stabilize kelp habitats and support key functional groups. Evidence from other temperate systems shows that historical overfishing can shift communities toward dominance by fishing-tolerant but climatically sensitive species, compounding anthropogenic and environmental pressures (McLean et al., 2019). Protecting the remaining canopy-forming kelp forests, reducing fishing pressure on vulnerable species, and limiting further urchin expansion through targeted culling while safeguarding their predators may help sustain the regenerative processes that underpin ecosystem recovery and prevent long-term functional collapse (Roberts et al., 2024). Importantly, such interventions are most effective when embedded within broader nature-based solution frameworks that combine ecological restoration with co-interventions addressing socio-economic drivers and stakeholder trade-offs (Filz et al., 2025). Proactive fisheries management, when coupled with ecosystem restoration, is therefore likely to be critical in preventing kelp forests in Baja California from crossing ecological thresholds beyond which recovery becomes increasingly constrained.

Our study integrates taxonomic, functional, and productivity-based perspectives across subregions and years, providing a more comprehensive understanding of fish community reorganization. Several limitations should nevertheless be considered. Because monitoring began after the 2014–2016 heatwave, pre-disturbance baselines are not available, and we cannot determine whether the three subregions originated from a common ecosystem state or whether pre-existing regional differences were amplified by the heatwave. Our inferences therefore concern divergence from the early post-heatwave baseline (2017–2018), not from a pristine condition. The eight-year dataset may also be too short to capture the slower demographic responses of long-lived temperate fishes, which often unfold across decades (Moraes et al., 2012). Fishing exerts direct and indirect effects that interact with habitat loss, and CONAPESCA landings provide only a coarse proxy of fishing pressure, subject to reporting uncertainty and spatial aggregation. Uncertainty about fishing activity within and among nominally protected sites, combined with the averaging of fished and non-fished sites per subregion, further constrains attribution to any single driver.

Nevertheless, the consistency of our results across taxonomic and functional metrics indicates that the regional trajectories in community composition, functional structure, and biomass dynamics are robust despite these constraints. The persistence of functional differences under conservative assumptions suggests that longer time series are likely to refine the magnitude of change rather than alter the direction of the regional patterns observed. A more focused analysis incorporating fisheries data will be key to understanding how fishing interacts with habitat change to shape fish functional traits in Baja California. Continued monitoring combined with high-quality fisheries data will further be essential to assess local stressors and determine whether current trends represent long-term shifts in functional ecosystem state. Such data will also allow evaluation of whether remaining kelp forests retain the capacity to buffer further disturbance.

## Conclusion

This study tested whether reef-fish assemblages can sustain ecosystem functions as kelp forests reorganize into contrasting post-heatwave states along the Baja California peninsula. We hypothesized that biomass would decline in the most disturbed subregions, that taxonomic diversity would remain more stable than functional diversity, that assemblages would diverge progressively from their early post-heatwave state, and that habitat reorganization would reduce functional stability through changes in body size and biomass turnover. Our findings partly support these predictions and partly qualify them. Consistent with the second hypothesis, fish species richness and functional *α*-diversity remained stable across all subregions, yet this apparent stability masked contrasting regional dynamics in composition and function. In the North, assemblages showed little temporal change, consistent with persistent urchin-barren conditions and a compositionally constrained fish assemblage. In the Middle, functional *β*-decay increased over time through coordinated shifts in trophic, growth, and body-size traits rather than a single dominant driver. The South showed the broadest reorganization, with increases in both taxonomic and functional *β*-decay and the strongest signal in maximum fecundity. Supporting the first and fourth hypotheses, macroinvertivore biomass declined significantly in the North and showed a pronounced, though non-significant, decline in the South, and these losses were not offset by increased biomass turnover in other functional groups.

Our inferences are bounded by three constraints. First, because monitoring began after the 2014–2016 heatwave, our analyses capture post-disturbance trajectories rather than direct shifts from pre-heatwave conditions, and we cannot determine whether the subregions are diverging from, converging toward, or otherwise departing from their pre-heatwave state. Second, fishing pressure and habitat change co-occur across subregions, and their relative contributions cannot be fully disentangled with the available data. Third, the eight-year time series may be insufficient to capture the slower demographic responses of long-lived temperate fishes. We speculate that the lack of compensatory turnover observed here signals an early stage of functional erosion that may become more pronounced as further warming and fishing pressure accumulate; testing this will require longer time series and explicit coupling to habitat recovery dynamics. Future work should therefore (i) extend long-term monitoring across all three subregions, (ii) integrate spatially resolved fisheries data to isolate fishing from habitat effects, (iii) directly link fish functional processes to kelp recovery, and (iv) incorporate pre-heatwave baselines from adjacent regions where available, to anchor inferences about resilience more firmly. By integrating taxonomic diversity, functional traits, and biomass dynamics, our study demonstrates a clear decoupling between biodiversity patterns and ecosystem functioning in post-heatwave kelp-forest fish assemblages. Stable diversity metrics can conceal substantial reorganization in how assemblages produce and sustain biomass. Safeguarding these processes will require a shift from tracking diversity alone to directly monitoring and managing functional groups and biomass dynamics.

##  Supplemental Information

10.7717/peerj.21452/supp-1 Supplemental Information 1 Species-specific body-size trends

10.7717/peerj.21452/supp-2 Supplemental Information 2 SIMPER clustering dendrograms

10.7717/peerj.21452/supp-3 Supplemental Information 3 Supplemental tables and figures

## Additional Information and Declarations

### Data Availability

The following information was supplied regarding data availability: The data used in this study are owned by the MexCal program at the Universidad Autónoma de Baja California (UABC). The MexCal dataset is public but contains sensitive data related to fishers and managers. The de-identified MexCal dataset is available at GitHub and Zenodo: – https://github.com/rbeas/mexcal-data-access. – Rodrigo Beas. (2026). rbeas/mexcal-data-access: PeerJ-130524 (V1.0.0). Zenodo. https://doi.org/10.5281/zenodo.20400246. Further information about the program is available at https://www.mex-cal.org. NOAA Optimum Interpolation Sea Surface Temperature version 2.1 (OISST v2.1) data is available at: https://coastwatch.pfeg.noaa.gov/erddap/griddap/ncdcOisst21Agg_LonPM180.html. The specific subset used in this study was queried with the following parameters: Latitude: 23° to 32.75°N Longitude: −119° to −110° To compute productivity and biomass turnover, we used the rfishprod package, which is available on GitHub: https://github.com/renatoamorais/rfishprod. The analysis is available at Zenodo: Filz, P. (2026). Diversity persists while function shifts: post-heatwave reorganization of reef fish communities in Baja California [Data set]. Zenodo. https://doi.org/10.5281/zenodo.19712775.
