# Supplementary figures and images for: Diversity persists while function shifts: post-heatwave reorganization of reef fish communities in Baja California
Source: PeerJ. 2026 Jul 8;14:e21452. doi: 10.7717/peerj.21452 (PMC13355618; doi:10.7717/peerj.21452)

North

South

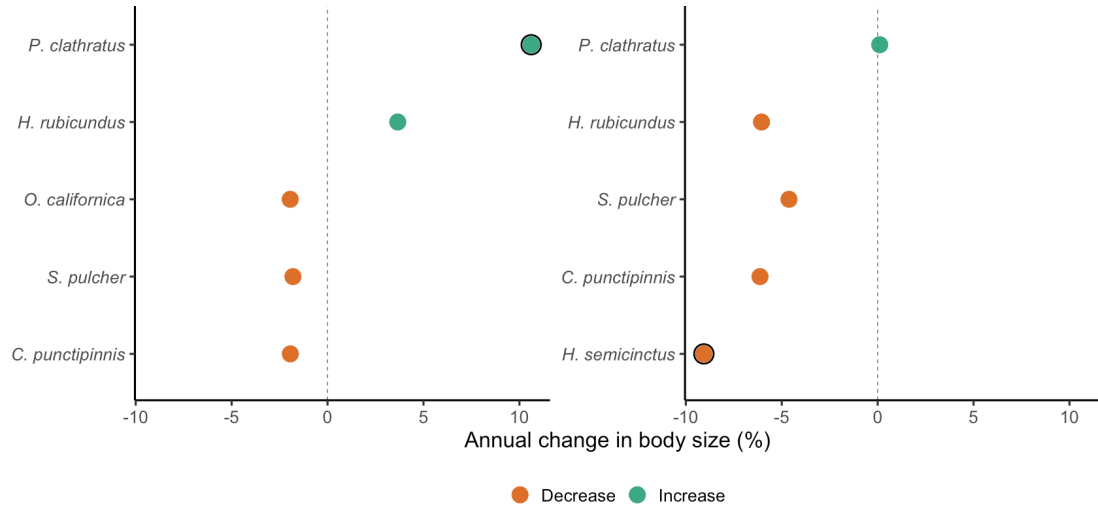

Supplement: Supplemental Information 1 [file peerj-14-21452-s001.pdf]

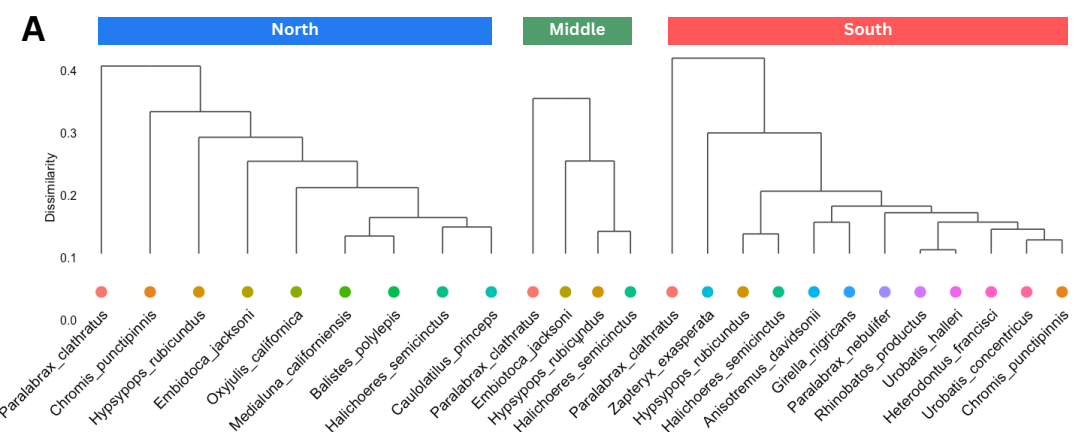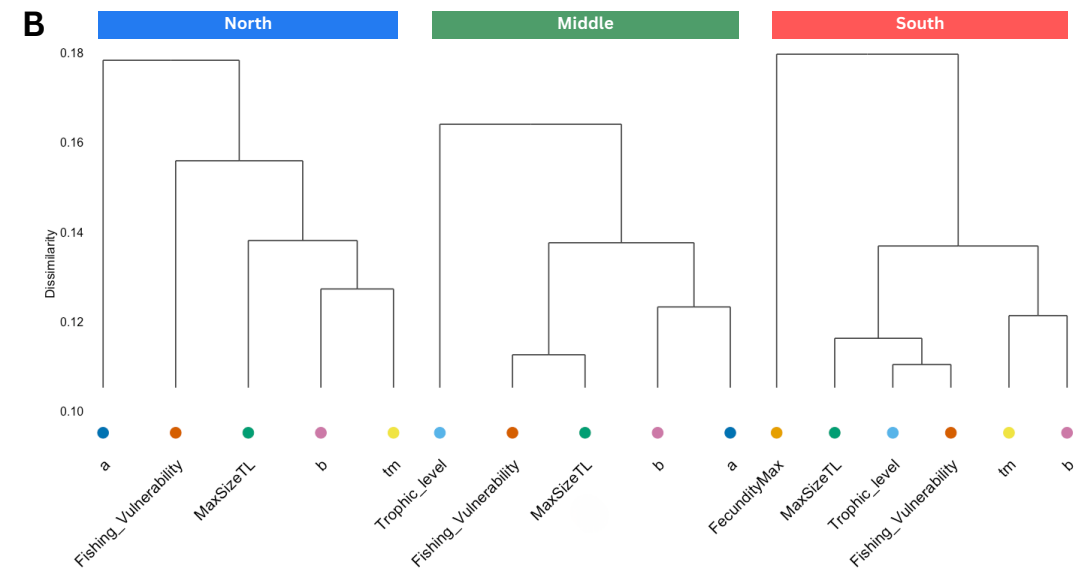

Supplement: Supplemental Information 2 [file peerj-14-21452-s002.pdf]
