# Supplementary material for: Diversity persists while function shifts: post-heatwave reorganization of reef fish communities in Baja California
Source: PeerJ. 2026 Jul 8;14:e21452. doi: 10.7717/peerj.21452 (PMC13355618; doi:10.7717/peerj.21452)

**Supplemental Materials**

*Diversity persists while function shifts: reef-fish responses to post-heatwave kelp-forest reorganisation in Baja California*

**Contents**

**Table S1** Sampling design and effort

**Table S2** Fixed-effects estimates from GLMMs for fish biomass, algae density, and invertebrate abundance

**Table S3** Dominant species by subregion and functional group

**Table S4** GLMM results for α-diversity and β-decay

**Table S5** Observed values of taxonomic and functional α-diversity

**Table S6** Observed values of taxonomic and functional β-decay

**Table S7** Combined taxonomic and functional SIMPER results across subregions and year-pairs

**Table S8** Temporal trends in biomass of SIMPER-identified top species

**Table S9** GLMM results for biomass-weighted fish size

**Table S10** Pairwise comparisons of biomass-weighted fish size among subregions

**Table S11** Species-specific mixed-effects results for temporal trends in body size

**Figure S1** Species-specific body size trends

**Table S12** GLMM results for biomass turnover (P/B)

**Table S13** Major taxonomic contributors to temporal community change (SIMPER)

**Table S14** Major functional contributors to temporal community change (SIMPER)

**Figure S2** SIMPER clustering dendrograms

**Table S1 | Sampling design and effort**

Detailed sampling structure of reef-fish surveys across subregions (North, Middle, South), sites, and years (2017–2024). The table reports the number of belt transects (30 × 2 m; 60 m²) conducted per site and year, along with the number of subsites sampled. Transects represent the smallest sampling unit and were nested within subsites and sites. This hierarchical structure and uneven sampling effort were explicitly accounted for in all statistical analyses using generalised linear mixed-effects models (GLMMs) with Site (and Sub-site where appropriate) as random intercepts.

| **Subregion** | **Site** | **Year** | **n transects** | **n subsites** |
| --- | --- | --- | --- | --- |
| North | Campo Kennedy | 2018 | 3 | 1 |
| North | Campo Kennedy | 2019 | 6 | 3 |
| North | Campo Kennedy | 2020 | 5 | 3 |
| North | Campo Kennedy | 2021 | 6 | 3 |
| North | Campo Kennedy | 2022 | 3 | 1 |
| North | Gaviotas | 2024 | 1 | 1 |
| North | Isla Todos Santos | 2017 | 6 | 3 |
| North | Isla Todos Santos | 2018 | 6 | 3 |
| North | Isla Todos Santos | 2019 | 5 | 3 |
| North | Isla Todos Santos | 2020 | 12 | 5 |
| North | Isla Todos Santos | 2021 | 6 | 3 |
| North | Isla Todos Santos | 2022 | 5 | 3 |
| North | Isla Todos Santos | 2023 | 9 | 4 |
| North | Isla Todos Santos | 2024 | 4 | 2 |
| North | Islas Coronados | 2021 | 10 | 5 |
| North | Islas Coronados | 2022 | 19 | 10 |
| North | Islas Coronados | 2023 | 28 | 5 |
| North | Punta Banda | 2018 | 3 | 1 |
| North | Punta Banda | 2019 | 6 | 3 |
| North | Punta Banda | 2020 | 6 | 3 |
| North | Punta Banda | 2022 | 6 | 3 |
| North | Punta Banda | 2023 | 13 | 3 |
| North | San Miguel | 2017 | 4 | 2 |
| North | San Miguel | 2018 | 24 | 11 |
| North | San Miguel | 2020 | 6 | 3 |
| North | San Miguel | 2023 | 10 | 3 |
| Middle | Isla San Jeronimo | 2017 | 14 | 5 |
| Middle | Isla San Jeronimo | 2018 | 6 | 3 |
| Middle | Isla San Jeronimo | 2019 | 12 | 6 |
| Middle | Isla San Jeronimo | 2020 | 10 | 5 |
| Middle | Isla San Jeronimo | 2022 | 4 | 2 |
| Middle | Isla San Jeronimo | 2023 | 9 | 5 |
| Middle | Isla San Jeronimo | 2024 | 8 | 4 |
| Middle | Isla San Martin | 2017 | 12 | 4 |
| Middle | Isla San Martin | 2020 | 6 | 3 |
| Middle | Isla San Martin | 2023 | 6 | 3 |
| Middle | Isla San Martin | 2024 | 8 | 3 |
| Middle | Punta Baja | 2022 | 2 | 1 |
| South | Gavilanes | 2017 | 6 | 3 |
| South | Gavilanes | 2018 | 6 | 3 |
| South | Gavilanes | 2019 | 6 | 3 |
| South | Gavilanes | 2020 | 6 | 3 |
| South | Gavilanes | 2021 | 6 | 3 |
| South | Gavilanes | 2022 | 18 | 1 |
| South | Gavilanes | 2023 | 17 | 3 |
| South | Gavilanes | 2024 | 12 | 2 |
| South | Isla San Roque | 2019 | 6 | 3 |
| South | Isla San Roque | 2020 | 6 | 3 |
| South | Isla San Roque | 2022 | 18 | 1 |
| South | Isla San Roque | 2023 | 18 | 3 |
| South | Isla San Roque | 2024 | 6 | 3 |
| South | Piedra Blanca | 2017 | 6 | 3 |
| South | Piedra Blanca | 2018 | 6 | 3 |
| South | Piedra Blanca | 2019 | 6 | 3 |
| South | Piedra Blanca | 2020 | 6 | 3 |
| South | Piedra Blanca | 2021 | 6 | 3 |
| South | Piedra Blanca | 2022 | 36 | 5 |
| South | Piedra Blanca | 2023 | 18 | 3 |
| South | Piedra Blanca | 2024 | 22 | 3 |
| Total |  |  | 546 |  |

**Table S2 | Fixed-effects estimates from GLMMs for fish biomass, algae density, and invertebrate abundance**

Separate GLMMs were fitted to transect-level data for each of three response variables: fish biomass (g m⁻²), algae density (individuals m⁻²), and invertebrate abundance (individuals m⁻²). All three response variables were log₁₊-transformed prior to modelling. Models were fitted with the glmmTMB R package. Fixed effects include Year (centred and scaled), Subregion (North, Middle, South; North as reference), functional-group identity (reference levels: Herbivore for fish and invertebrates, canopy kelp for algae), and all two- and three-way interactions. Site was included as a random intercept. Estimates (β) and standard errors (SE) are on the log scale. Significance codes: *** p < 0.001, ** p < 0.01, * p < 0.05, . p < 0.1.

| **Response** | **Term** | **Estimate** | **SE** | **z** | **p** |  |
| --- | --- | --- | --- | --- | --- | --- |
| Fish biomass | (Intercept) | 6.342 | 0.171 | 37.02 | <0.001 | *** |
| Fish biomass | Year | 0.096 | 0.126 | 0.76 | 0.448 |  |
| Fish biomass | Middle | 0.209 | 0.270 | 0.77 | 0.439 |  |
| Fish biomass | South | -0.007 | 0.243 | -0.03 | 0.978 |  |
| Fish biomass | Macroinvertivore | 0.374 | 0.150 | 2.50 | 0.013 | * |
| Fish biomass | Microinvertivore | -0.271 | 0.164 | -1.65 | 0.099 | . |
| Fish biomass | Piscivore | -0.183 | 0.157 | -1.17 | 0.241 |  |
| Fish biomass | Planktivore | -0.199 | 0.159 | -1.25 | 0.212 |  |
| Fish biomass | Year × Middle | -0.122 | 0.159 | -0.76 | 0.445 |  |
| Fish biomass | Year × South | -0.317 | 0.166 | -1.91 | 0.056 | . |
| Fish biomass | Year × Macroinvertivore | -0.372 | 0.140 | -2.66 | 0.008 | ** |
| Fish biomass | Year × Microinvertivore | -0.324 | 0.149 | -2.17 | 0.030 | * |
| Fish biomass | Year × Piscivore | -0.221 | 0.145 | -1.53 | 0.127 |  |
| Fish biomass | Year × Planktivore | -0.190 | 0.150 | -1.26 | 0.207 |  |
| Fish biomass | Middle × Macroinvertivore | 0.379 | 0.236 | 1.61 | 0.108 |  |
| Fish biomass | South × Macroinvertivore | 0.200 | 0.194 | 1.03 | 0.302 |  |
| Fish biomass | Middle × Microinvertivore | 0.141 | 0.260 | 0.54 | 0.588 |  |
| Fish biomass | South × Microinvertivore | 0.235 | 0.208 | 1.13 | 0.259 |  |
| Fish biomass | Middle × Piscivore | 0.113 | 0.260 | 0.43 | 0.665 |  |
| Fish biomass | South × Piscivore | 0.565 | 0.200 | 2.83 | 0.005 | ** |
| Fish biomass | Middle × Planktivore | 0.402 | 0.255 | 1.58 | 0.115 |  |
| Fish biomass | South × Planktivore | -0.390 | 0.212 | -1.83 | 0.067 | . |
| Fish biomass | Year × Middle × Macroinvertivore | 0.438 | 0.188 | 2.33 | 0.020 | * |
| Fish biomass | Year × South × Macroinvertivore | 0.188 | 0.188 | 1.00 | 0.316 |  |
| Fish biomass | Year × Middle × Microinvertivore | 0.348 | 0.201 | 1.73 | 0.083 | . |
| Fish biomass | Year × South × Microinvertivore | 0.218 | 0.198 | 1.10 | 0.270 |  |
| Fish biomass | Year × Middle × Piscivore | 0.136 | 0.195 | 0.69 | 0.487 |  |
| Fish biomass | Year × South × Piscivore | 0.043 | 0.193 | 0.22 | 0.824 |  |
| Fish biomass | Year × Middle × Planktivore | 0.136 | 0.201 | 0.68 | 0.498 |  |
| Fish biomass | Year × South × Planktivore | 0.183 | 0.204 | 0.90 | 0.369 |  |
| Algae density | (Intercept) | 10.410 | 0.179 | 58.21 | <0.001 | *** |
| Algae density | Year | -0.239 | 0.118 | -2.02 | 0.043 | * |
| Algae density | Middle | 0.227 | 0.281 | 0.81 | 0.418 |  |
| Algae density | South | 1.637 | 0.233 | 7.03 | <0.001 | *** |
| Algae density | encrusting coralline | -0.764 | 0.759 | -1.01 | 0.314 |  |
| Algae density | subcanopy kelp | -0.494 | 0.160 | -3.09 | 0.002 | ** |
| Algae density | understory brown | 0.053 | 0.155 | 0.34 | 0.731 |  |
| Algae density | understory coralline | -0.308 | 0.689 | -0.45 | 0.654 |  |
| Algae density | understory green | -1.862 | 0.882 | -2.11 | 0.035 | * |
| Algae density | understory kelp | -0.387 | 0.175 | -2.21 | 0.027 | * |
| Algae density | understory red | -0.449 | 0.830 | -0.54 | 0.589 |  |
| Algae density | Year × Middle | -0.080 | 0.176 | -0.46 | 0.648 |  |
| Algae density | Year × South | -0.385 | 0.131 | -2.94 | 0.003 | ** |
| Algae density | Year × encrusting coralline | NA | NA | NA | NA | NA |
| Algae density | Year × subcanopy kelp | 0.228 | 0.165 | 1.38 | 0.166 |  |
| Algae density | Year × understory brown | 0.123 | 0.148 | 0.83 | 0.405 |  |
| Algae density | Year × understory coralline | NA | NA | NA | NA | NA |
| Algae density | Year × understory green | 0.256 | 1.226 | 0.21 | 0.835 |  |
| Algae density | Year × understory kelp | -0.161 | 0.192 | -0.84 | 0.402 |  |
| Algae density | Year × understory red | 0.193 | 0.948 | 0.20 | 0.839 |  |
| Algae density | Middle × encrusting coralline | NA | NA | NA | NA | NA |
| Algae density | South × encrusting coralline | NA | NA | NA | NA | NA |
| Algae density | Middle × subcanopy kelp | 0.686 | 0.249 | 2.75 | 0.006 | ** |
| Algae density | South × subcanopy kelp | -0.832 | 0.193 | -4.31 | <0.001 | *** |
| Algae density | Middle × understory brown | -0.281 | 0.296 | -0.95 | 0.343 |  |
| Algae density | South × understory brown | -2.537 | 0.245 | -10.37 | <0.001 | *** |
| Algae density | Middle × understory coralline | NA | NA | NA | NA | NA |
| Algae density | South × understory coralline | NA | NA | NA | NA | NA |
| Algae density | Middle × understory green | 0.077 | 1.079 | 0.07 | 0.944 |  |
| Algae density | South × understory green | -1.742 | 1.343 | -1.30 | 0.195 |  |
| Algae density | Middle × understory kelp | -0.626 | 0.312 | -2.00 | 0.045 | * |
| Algae density | South × understory kelp | -2.212 | 0.271 | -8.15 | <0.001 | *** |
| Algae density | Middle × understory red | -0.318 | 1.476 | -0.21 | 0.830 |  |
| Algae density | South × understory red | -1.358 | 0.759 | -1.79 | 0.073 | . |
| Algae density | Year × Middle × encrusting coralline | NA | NA | NA | NA | NA |
| Algae density | Year × South × encrusting coralline | NA | NA | NA | NA | NA |
| Algae density | Year × Middle × subcanopy kelp | -0.089 | 0.226 | -0.39 | 0.694 |  |
| Algae density | Year × South × subcanopy kelp | 0.116 | 0.194 | 0.59 | 0.552 |  |
| Algae density | Year × Middle × understory brown | 0.223 | 0.264 | 0.84 | 0.398 |  |
| Algae density | Year × South × understory brown | 0.391 | 0.218 | 1.79 | 0.073 | . |
| Algae density | Year × Middle × understory coralline | NA | NA | NA | NA | NA |
| Algae density | Year × South × understory coralline | NA | NA | NA | NA | NA |
| Algae density | Year × Middle × understory green | -0.341 | 1.423 | -0.24 | 0.810 |  |
| Algae density | Year × South × understory green | NA | NA | NA | NA | NA |
| Algae density | Year × Middle × understory kelp | 0.197 | 0.282 | 0.70 | 0.484 |  |
| Algae density | Year × South × understory kelp | 0.394 | 0.283 | 1.39 | 0.164 |  |
| Algae density | Year × Middle × understory red | NA | NA | NA | NA | NA |
| Algae density | Year × South × understory red | NA | NA | NA | NA | NA |
| Invertebrate abundance | (Intercept) | 9.047 | 0.166 | 54.54 | <0.001 | *** |
| Invertebrate abundance | Year | 0.087 | 0.148 | 0.59 | 0.554 |  |
| Invertebrate abundance | Middle | -0.415 | 0.293 | -1.42 | 0.156 |  |
| Invertebrate abundance | South | -0.389 | 0.224 | -1.74 | 0.082 | . |
| Invertebrate abundance | herbivore | 1.156 | 0.149 | 7.73 | <0.001 | *** |
| Invertebrate abundance | macroinvertivore | 0.323 | 0.163 | 1.98 | 0.048 | * |
| Invertebrate abundance | microinvertivore | 0.548 | 0.158 | 3.46 | <0.001 | *** |
| Invertebrate abundance | suspension feeder | 0.215 | 0.170 | 1.27 | 0.204 |  |
| Invertebrate abundance | urchins | 1.083 | 0.154 | 7.01 | <0.001 | *** |
| Invertebrate abundance | Year × Middle | -0.143 | 0.244 | -0.59 | 0.558 |  |
| Invertebrate abundance | Year × South | -0.141 | 0.200 | -0.70 | 0.481 |  |
| Invertebrate abundance | Year × herbivore | -0.042 | 0.161 | -0.26 | 0.792 |  |
| Invertebrate abundance | Year × macroinvertivore | -0.117 | 0.173 | -0.68 | 0.497 |  |
| Invertebrate abundance | Year × microinvertivore | 0.015 | 0.168 | 0.09 | 0.931 |  |
| Invertebrate abundance | Year × suspension feeder | -0.248 | 0.176 | -1.41 | 0.158 |  |
| Invertebrate abundance | Year × urchins | 0.021 | 0.166 | 0.13 | 0.900 |  |
| Invertebrate abundance | Middle × herbivore | 0.546 | 0.297 | 1.84 | 0.065 | . |
| Invertebrate abundance | South × herbivore | 0.070 | 0.211 | 0.33 | 0.739 |  |
| Invertebrate abundance | Middle × macroinvertivore | 0.071 | 0.333 | 0.21 | 0.831 |  |
| Invertebrate abundance | South × macroinvertivore | 0.199 | 0.233 | 0.86 | 0.392 |  |
| Invertebrate abundance | Middle × microinvertivore | -0.175 | 0.344 | -0.51 | 0.611 |  |
| Invertebrate abundance | South × microinvertivore | -0.828 | 0.286 | -2.89 | 0.004 | ** |
| Invertebrate abundance | Middle × suspension feeder | 0.597 | 0.326 | 1.83 | 0.067 | . |
| Invertebrate abundance | South × suspension feeder | 0.637 | 0.233 | 2.74 | 0.006 | ** |
| Invertebrate abundance | Middle × urchins | -0.092 | 0.307 | -0.30 | 0.763 |  |
| Invertebrate abundance | South × urchins | -1.258 | 0.354 | -3.56 | <0.001 | *** |
| Invertebrate abundance | Year × Middle × herbivore | 0.298 | 0.274 | 1.09 | 0.276 |  |
| Invertebrate abundance | Year × South × herbivore | -0.265 | 0.220 | -1.21 | 0.227 |  |
| Invertebrate abundance | Year × Middle × macroinvertivore | 0.273 | 0.304 | 0.90 | 0.369 |  |
| Invertebrate abundance | Year × South × macroinvertivore | -0.147 | 0.241 | -0.61 | 0.542 |  |
| Invertebrate abundance | Year × Middle × microinvertivore | 0.281 | 0.328 | 0.86 | 0.392 |  |
| Invertebrate abundance | Year × South × microinvertivore | -0.257 | 0.281 | -0.91 | 0.362 |  |
| Invertebrate abundance | Year × Middle × suspension feeder | 0.435 | 0.301 | 1.44 | 0.149 |  |
| Invertebrate abundance | Year × South × suspension feeder | -0.270 | 0.240 | -1.13 | 0.260 |  |
| Invertebrate abundance | Year × Middle × urchins | 0.434 | 0.295 | 1.47 | 0.141 |  |
| Invertebrate abundance | Year × South × urchins | 0.143 | 0.372 | 0.38 | 0.701 |  |

**Table S3 | Dominant species by subregion and functional group**

Dominant species (by mean density for algae and invertebrates, and mean biomass for fishes) in each subregion and functional group, based on pooled 2017–2024 transect-level data.

| **Subregion** | **Functional group** | **Species** | **Value** | **Taxon** |
| --- | --- | --- | --- | --- |
| Section | Function | *Genusspecies* | Value | Type |
| North | Canopy kelp | *Macrocystis pyrifera juvenile* | 0.46 | Algae |
| North | Urchins | *Strongylocentrotus purpuratus* | 50.28 | Invertebrate |
| North | Macroinvertivore | *Balistes polylepis* | 162.21 | Fish |
| Middle | Canopy kelp | *Macrocystis pyrifera stipes* | 0.17 | Algae |
| Middle | Herbivore | *Megastrea spp* | 7.43 | Invertebrate |
| Middle | Macroinvertivore | *Zapteryx exasperata* | 208.51 | Fish |
| South | Canopy kelp | *Macrocystis pyrifera* | 0.33 | Algae |
| South | Herbivore | *Megastraea undosa* | 7.35 | Invertebrate |
| South | Macroinvertivore | *Zapteryx exasperata* | 164.39 | Fish |

**Table S4 | GLMM results for α-diversity and β-decay**

Results of generalised linear mixed-effects models (GLMMs) for taxonomic and functional α-diversity (Hill numbers at q = 0 and q = 1) and β-decay (dissimilarity relative to the 2017–2018 baseline). α-diversity models used a Gamma error distribution with a log link; β-decay models used a beta error distribution with a logit link. Fixed effects: Subregion (North = reference), Year (centred and scaled), and their interaction. Site included as a random intercept. Significance codes: *** p < 0.001, ** p < 0.01, * p < 0.05, . p < 0.1.

| **Model** | **Term** | **Estimate** | **SE** | **z** | **p** |  | **R² marg.** | **R² cond.** |
| --- | --- | --- | --- | --- | --- | --- | --- | --- |
| α taxonomic (q = 0) | (Intercept) | 1.690 | 0.056 | 30.36 | <0.001 | *** | 0.037 | 0.113 |
| α taxonomic (q = 0) | Middle | 0.130 | 0.073 | 1.77 | 0.078 |  | 0.037 | 0.113 |
| α taxonomic (q = 0) | South | 0.081 | 0.059 | 1.38 | 0.169 |  | 0.037 | 0.113 |
| α taxonomic (q = 0) | Year | -0.016 | 0.023 | -0.71 | 0.476 |  | 0.037 | 0.113 |
| α taxonomic (q = 0) | Middle × Year | 0.002 | 0.024 | 0.08 | 0.938 |  | 0.037 | 0.113 |
| α taxonomic (q = 0) | South × Year | -0.031 | 0.021 | -1.45 | 0.147 |  | 0.037 | 0.113 |
| α taxonomic (q = 1) | (Intercept) | 1.190 | 0.057 | 21.06 | <0.001 | *** | 0.024 | 0.103 |
| α taxonomic (q = 1) | Middle | 0.107 | 0.093 | 1.15 | 0.250 |  | 0.024 | 0.103 |
| α taxonomic (q = 1) | South | 0.042 | 0.079 | 0.53 | 0.594 |  | 0.024 | 0.103 |
| α taxonomic (q = 1) | Year | -0.021 | 0.019 | -1.10 | 0.273 |  | 0.024 | 0.103 |
| α taxonomic (q = 1) | Middle × Year | 0.002 | 0.023 | 0.08 | 0.934 |  | 0.024 | 0.103 |
| α taxonomic (q = 1) | South × Year | -0.003 | 0.021 | -0.14 | 0.890 |  | 0.024 | 0.103 |
| α functional (q = 0) | (Intercept) | 1.284 | 0.040 | 31.78 | <0.001 | *** | 0.049 | 0.109 |
| α functional (q = 0) | Middle | 0.160 | 0.058 | 2.76 | 0.006 | ** | 0.049 | 0.109 |
| α functional (q = 0) | South | 0.096 | 0.047 | 2.03 | 0.042 | * | 0.049 | 0.109 |
| α functional (q = 0) | Year | 0.005 | 0.016 | 0.33 | 0.742 |  | 0.049 | 0.109 |
| α functional (q = 0) | Middle × Year | -0.024 | 0.018 | -1.33 | 0.184 |  | 0.049 | 0.109 |
| α functional (q = 0) | South × Year | -0.029 | 0.017 | -1.75 | 0.081 |  | 0.049 | 0.109 |
| α functional (q = 1) | (Intercept) | 0.914 | 0.042 | 21.74 | <0.001 | *** | 0.035 | 0.093 |
| α functional (q = 1) | Middle | 0.142 | 0.072 | 1.96 | 0.050 | * | 0.035 | 0.093 |
| α functional (q = 1) | South | 0.064 | 0.061 | 1.04 | 0.298 |  | 0.035 | 0.093 |
| α functional (q = 1) | Year | -0.007 | 0.014 | -0.47 | 0.637 |  | 0.035 | 0.093 |
| α functional (q = 1) | Middle × Year | -0.015 | 0.018 | -0.84 | 0.399 |  | 0.035 | 0.093 |
| α functional (q = 1) | South × Year | -0.001 | 0.017 | -0.09 | 0.932 |  | 0.035 | 0.093 |
| β-decay taxonomic (q = 0) | (Intercept) | 0.887 | 0.252 | 3.52 | <0.001 | *** | 0.270 | 0.775 |
| β-decay taxonomic (q = 0) | Middle | -0.954 | 0.414 | -2.31 | 0.021 | * | 0.270 | 0.775 |
| β-decay taxonomic (q = 0) | South | -0.434 | 0.413 | -1.05 | 0.293 |  | 0.270 | 0.775 |
| β-decay taxonomic (q = 0) | Year | -0.021 | 0.047 | -0.45 | 0.652 |  | 0.270 | 0.775 |
| β-decay taxonomic (q = 0) | Middle × Year | -0.039 | 0.024 | -1.61 | 0.108 |  | 0.270 | 0.775 |
| β-decay taxonomic (q = 0) | South × Year | 0.134 | 0.026 | 5.21 | <0.001 | *** | 0.270 | 0.775 |
| β-decay taxonomic (q = 1) | (Intercept) | 0.884 | 0.303 | 2.91 | 0.004 | ** | 0.228 | 0.759 |
| β-decay taxonomic (q = 1) | Middle | -1.051 | 0.470 | -2.24 | 0.025 | * | 0.228 | 0.759 |
| β-decay taxonomic (q = 1) | South | -0.705 | 0.469 | -1.51 | 0.132 |  | 0.228 | 0.759 |
| β-decay taxonomic (q = 1) | Year | -0.001 | 0.079 | -0.01 | 0.990 |  | 0.228 | 0.759 |
| β-decay taxonomic (q = 1) | Middle × Year | 0.014 | 0.028 | 0.49 | 0.622 |  | 0.228 | 0.759 |
| β-decay taxonomic (q = 1) | South × Year | 0.116 | 0.029 | 3.95 | <0.001 | *** | 0.228 | 0.759 |
| β-decay functional (q = 0) | (Intercept) | -1.792 | 0.142 | -12.65 | <0.001 | *** | 0.017 | 0.063 |
| β-decay functional (q = 0) | Middle | -0.178 | 0.217 | -0.82 | 0.412 |  | 0.017 | 0.063 |
| β-decay functional (q = 0) | South | -0.333 | 0.215 | -1.55 | 0.121 |  | 0.017 | 0.063 |
| β-decay functional (q = 0) | Year | -0.049 | 0.038 | -1.28 | 0.202 |  | 0.017 | 0.063 |
| β-decay functional (q = 0) | Middle × Year | 0.107 | 0.030 | 3.51 | <0.001 | *** | 0.017 | 0.063 |
| β-decay functional (q = 0) | South × Year | 0.102 | 0.030 | 3.37 | <0.001 | *** | 0.017 | 0.063 |
| β-decay functional (q = 1) | (Intercept) | -0.632 | 0.115 | -5.49 | <0.001 | *** | 0.023 | 0.127 |
| β-decay functional (q = 1) | Middle | -0.212 | 0.171 | -1.24 | 0.216 |  | 0.023 | 0.127 |
| β-decay functional (q = 1) | South | -0.229 | 0.169 | -1.35 | 0.176 |  | 0.023 | 0.127 |
| β-decay functional (q = 1) | Year | 0.008 | 0.036 | 0.22 | 0.826 |  | 0.023 | 0.127 |
| β-decay functional (q = 1) | Middle × Year | 0.007 | 0.026 | 0.28 | 0.779 |  | 0.023 | 0.127 |
| β-decay functional (q = 1) | South × Year | 0.034 | 0.027 | 1.28 | 0.200 |  | 0.023 | 0.127 |

**Table S5 | Observed values of taxonomic and functional α-diversity**

Descriptive statistics for taxonomic and functional α-diversity (Hill numbers at q = 0 and q = 1), calculated at the transect level and summarised per subregion and year. Abbreviations: n, number of transects; mean, arithmetic mean; sd, standard deviation; se, standard error; min and max, observed range.

| **Metric** | **Subregion** | **Year** | **n** | **Mean** | **SD** | **SE** | **Min** | **Max** |
| --- | --- | --- | --- | --- | --- | --- | --- | --- |
| α functional (q = 0) | North | 2017 | 10 | 4.284 | 0.484 | 0.153 | 3.463 | 5.141 |
| α functional (q = 0) | North | 2018 | 36 | 3.324 | 1.094 | 0.182 | 1.000 | 5.236 |
| α functional (q = 0) | North | 2019 | 17 | 2.812 | 1.140 | 0.277 | 1.000 | 5.029 |
| α functional (q = 0) | North | 2020 | 29 | 3.626 | 1.151 | 0.214 | 1.000 | 5.441 |
| α functional (q = 0) | North | 2021 | 22 | 4.097 | 0.952 | 0.203 | 1.000 | 5.229 |
| α functional (q = 0) | North | 2022 | 33 | 3.911 | 1.108 | 0.193 | 1.000 | 5.733 |
| α functional (q = 0) | North | 2023 | 60 | 3.387 | 1.006 | 0.130 | 1.000 | 5.657 |
| α functional (q = 0) | North | 2024 | 5 | 3.906 | 1.717 | 0.768 | 2.000 | 6.264 |
| α functional (q = 0) | Middle | 2017 | 26 | 4.797 | 0.705 | 0.138 | 3.000 | 5.642 |
| α functional (q = 0) | Middle | 2018 | 6 | 4.364 | 0.760 | 0.310 | 3.383 | 5.029 |
| α functional (q = 0) | Middle | 2019 | 12 | 3.893 | 1.024 | 0.296 | 2.000 | 4.946 |
| α functional (q = 0) | Middle | 2020 | 16 | 4.429 | 1.165 | 0.291 | 2.000 | 5.510 |
| α functional (q = 0) | Middle | 2022 | 6 | 3.410 | 1.325 | 0.541 | 1.056 | 4.628 |
| α functional (q = 0) | Middle | 2023 | 15 | 4.201 | 0.976 | 0.252 | 2.481 | 5.574 |
| α functional (q = 0) | Middle | 2024 | 16 | 4.141 | 0.994 | 0.249 | 1.056 | 5.395 |
| α functional (q = 0) | South | 2017 | 12 | 4.258 | 0.870 | 0.251 | 2.533 | 5.566 |
| α functional (q = 0) | South | 2018 | 12 | 4.544 | 0.655 | 0.189 | 3.502 | 5.628 |
| α functional (q = 0) | South | 2019 | 18 | 3.833 | 1.324 | 0.312 | 1.000 | 5.571 |
| α functional (q = 0) | South | 2020 | 18 | 4.746 | 0.867 | 0.204 | 2.551 | 5.961 |
| α functional (q = 0) | South | 2021 | 12 | 4.662 | 0.764 | 0.221 | 3.041 | 5.853 |
| α functional (q = 0) | South | 2022 | 72 | 3.293 | 1.000 | 0.118 | 1.000 | 5.579 |
| α functional (q = 0) | South | 2023 | 53 | 3.870 | 0.984 | 0.135 | 1.000 | 6.530 |
| α functional (q = 0) | South | 2024 | 40 | 3.909 | 1.313 | 0.208 | 1.000 | 5.897 |
| α functional (q = 1) | North | 2017 | 10 | 2.495 | 0.726 | 0.230 | 1.225 | 3.413 |
| α functional (q = 1) | North | 2018 | 36 | 2.383 | 0.830 | 0.138 | 1.000 | 4.616 |
| α functional (q = 1) | North | 2019 | 17 | 2.044 | 0.781 | 0.189 | 1.000 | 3.743 |
| α functional (q = 1) | North | 2020 | 29 | 2.934 | 1.028 | 0.191 | 1.000 | 5.042 |
| α functional (q = 1) | North | 2021 | 22 | 2.702 | 0.857 | 0.183 | 1.000 | 4.360 |
| α functional (q = 1) | North | 2022 | 33 | 2.662 | 1.019 | 0.177 | 1.000 | 5.204 |
| α functional (q = 1) | North | 2023 | 60 | 2.376 | 0.786 | 0.101 | 1.000 | 4.276 |
| α functional (q = 1) | North | 2024 | 5 | 2.062 | 1.004 | 0.449 | 1.088 | 3.436 |
| α functional (q = 1) | Middle | 2017 | 26 | 3.228 | 0.649 | 0.127 | 2.155 | 4.268 |
| α functional (q = 1) | Middle | 2018 | 6 | 3.041 | 0.675 | 0.276 | 2.357 | 3.986 |
| α functional (q = 1) | Middle | 2019 | 12 | 2.659 | 0.810 | 0.234 | 1.469 | 4.154 |
| α functional (q = 1) | Middle | 2020 | 16 | 2.783 | 0.955 | 0.239 | 1.365 | 4.539 |
| α functional (q = 1) | Middle | 2022 | 6 | 2.693 | 1.012 | 0.413 | 1.008 | 3.689 |
| α functional (q = 1) | Middle | 2023 | 15 | 2.838 | 0.753 | 0.194 | 1.129 | 4.065 |
| α functional (q = 1) | Middle | 2024 | 16 | 2.724 | 0.916 | 0.229 | 1.050 | 4.602 |
| α functional (q = 1) | South | 2017 | 12 | 3.065 | 0.684 | 0.197 | 2.159 | 4.461 |
| α functional (q = 1) | South | 2018 | 12 | 2.790 | 0.679 | 0.196 | 1.685 | 3.963 |
| α functional (q = 1) | South | 2019 | 18 | 2.587 | 0.769 | 0.181 | 1.000 | 3.828 |
| α functional (q = 1) | South | 2020 | 18 | 2.868 | 0.615 | 0.145 | 1.694 | 4.326 |
| α functional (q = 1) | South | 2021 | 12 | 2.429 | 0.653 | 0.189 | 1.591 | 3.807 |
| α functional (q = 1) | South | 2022 | 72 | 2.436 | 0.836 | 0.099 | 1.000 | 4.079 |
| α functional (q = 1) | South | 2023 | 53 | 2.628 | 0.597 | 0.082 | 1.000 | 4.060 |
| α functional (q = 1) | South | 2024 | 40 | 2.799 | 0.975 | 0.154 | 1.000 | 4.749 |
| α taxonomic (q = 0) | North | 2017 | 10 | 6.900 | 1.449 | 0.458 | 4.000 | 9.000 |
| α taxonomic (q = 0) | North | 2018 | 36 | 5.500 | 2.720 | 0.453 | 1.000 | 11.000 |
| α taxonomic (q = 0) | North | 2019 | 17 | 4.647 | 2.290 | 0.555 | 1.000 | 9.000 |
| α taxonomic (q = 0) | North | 2020 | 29 | 5.414 | 2.383 | 0.443 | 1.000 | 9.000 |
| α taxonomic (q = 0) | North | 2021 | 22 | 6.273 | 2.142 | 0.457 | 1.000 | 11.000 |
| α taxonomic (q = 0) | North | 2022 | 33 | 5.515 | 2.048 | 0.357 | 1.000 | 10.000 |
| α taxonomic (q = 0) | North | 2023 | 60 | 4.733 | 2.146 | 0.277 | 1.000 | 12.000 |
| α taxonomic (q = 0) | North | 2024 | 5 | 5.400 | 3.209 | 1.435 | 2.000 | 10.000 |
| α taxonomic (q = 0) | Middle | 2017 | 26 | 7.192 | 1.674 | 0.328 | 3.000 | 10.000 |
| α taxonomic (q = 0) | Middle | 2018 | 6 | 5.500 | 1.378 | 0.563 | 4.000 | 7.000 |
| α taxonomic (q = 0) | Middle | 2019 | 12 | 5.000 | 1.706 | 0.492 | 2.000 | 7.000 |
| α taxonomic (q = 0) | Middle | 2020 | 16 | 6.562 | 2.190 | 0.547 | 2.000 | 9.000 |
| α taxonomic (q = 0) | Middle | 2022 | 6 | 5.167 | 2.229 | 0.910 | 2.000 | 8.000 |
| α taxonomic (q = 0) | Middle | 2023 | 15 | 5.867 | 1.598 | 0.413 | 3.000 | 8.000 |
| α taxonomic (q = 0) | Middle | 2024 | 16 | 6.438 | 1.788 | 0.447 | 2.000 | 9.000 |
| α taxonomic (q = 0) | South | 2017 | 12 | 6.750 | 2.417 | 0.698 | 3.000 | 11.000 |
| α taxonomic (q = 0) | South | 2018 | 12 | 8.250 | 2.667 | 0.770 | 5.000 | 13.000 |
| α taxonomic (q = 0) | South | 2019 | 18 | 5.111 | 2.026 | 0.478 | 1.000 | 8.000 |
| α taxonomic (q = 0) | South | 2020 | 18 | 7.833 | 2.307 | 0.544 | 3.000 | 12.000 |
| α taxonomic (q = 0) | South | 2021 | 12 | 7.083 | 2.151 | 0.621 | 4.000 | 12.000 |
| α taxonomic (q = 0) | South | 2022 | 72 | 4.375 | 1.496 | 0.176 | 1.000 | 8.000 |
| α taxonomic (q = 0) | South | 2023 | 53 | 5.358 | 1.642 | 0.226 | 1.000 | 9.000 |
| α taxonomic (q = 0) | South | 2024 | 40 | 5.800 | 2.554 | 0.404 | 1.000 | 11.000 |
| α taxonomic (q = 1) | North | 2017 | 10 | 3.302 | 1.282 | 0.405 | 1.281 | 5.275 |
| α taxonomic (q = 1) | North | 2018 | 36 | 3.280 | 1.387 | 0.231 | 1.000 | 6.498 |
| α taxonomic (q = 1) | North | 2019 | 17 | 2.977 | 1.577 | 0.383 | 1.000 | 6.229 |
| α taxonomic (q = 1) | North | 2020 | 29 | 3.968 | 1.726 | 0.321 | 1.000 | 7.205 |
| α taxonomic (q = 1) | North | 2021 | 22 | 3.627 | 1.415 | 0.302 | 1.000 | 6.199 |
| α taxonomic (q = 1) | North | 2022 | 33 | 3.337 | 1.517 | 0.264 | 1.000 | 7.529 |
| α taxonomic (q = 1) | North | 2023 | 60 | 3.003 | 1.294 | 0.167 | 1.000 | 7.558 |
| α taxonomic (q = 1) | North | 2024 | 5 | 2.424 | 1.326 | 0.593 | 1.088 | 4.035 |
| α taxonomic (q = 1) | Middle | 2017 | 26 | 4.256 | 1.141 | 0.224 | 2.438 | 6.266 |
| α taxonomic (q = 1) | Middle | 2018 | 6 | 3.508 | 0.932 | 0.381 | 2.487 | 5.002 |
| α taxonomic (q = 1) | Middle | 2019 | 12 | 2.983 | 0.961 | 0.277 | 1.544 | 4.565 |
| α taxonomic (q = 1) | Middle | 2020 | 16 | 3.660 | 1.606 | 0.401 | 1.365 | 6.505 |
| α taxonomic (q = 1) | Middle | 2022 | 6 | 3.264 | 1.301 | 0.531 | 1.169 | 5.002 |
| α taxonomic (q = 1) | Middle | 2023 | 15 | 3.575 | 1.248 | 0.322 | 1.243 | 6.202 |
| α taxonomic (q = 1) | Middle | 2024 | 16 | 3.708 | 1.530 | 0.383 | 1.863 | 6.915 |
| α taxonomic (q = 1) | South | 2017 | 12 | 4.274 | 1.488 | 0.430 | 2.353 | 6.844 |
| α taxonomic (q = 1) | South | 2018 | 12 | 4.034 | 1.401 | 0.405 | 2.231 | 6.409 |
| α taxonomic (q = 1) | South | 2019 | 18 | 3.134 | 1.056 | 0.249 | 1.000 | 4.927 |
| α taxonomic (q = 1) | South | 2020 | 18 | 4.031 | 1.386 | 0.327 | 1.786 | 7.247 |
| α taxonomic (q = 1) | South | 2021 | 12 | 3.139 | 0.936 | 0.270 | 1.773 | 4.684 |
| α taxonomic (q = 1) | South | 2022 | 72 | 2.970 | 1.108 | 0.131 | 1.000 | 5.127 |
| α taxonomic (q = 1) | South | 2023 | 53 | 3.224 | 0.916 | 0.126 | 1.000 | 5.471 |
| α taxonomic (q = 1) | South | 2024 | 40 | 3.621 | 1.568 | 0.248 | 1.000 | 6.970 |

**Table S6 | Observed values of taxonomic and functional β-decay**

Descriptive statistics for taxonomic and functional β-decay (Hill-based Jaccard dissimilarity relative to the 2017–2018 baseline) at q = 0 and q = 1, calculated at the transect level and summarised per subregion and year.

| **Metric** | **Subregion** | **Year** | **n** | **Mean** | **SD** | **SE** | **Min** | **Max** |
| --- | --- | --- | --- | --- | --- | --- | --- | --- |
| β-decay functional (q = 0) | North | 2019 | 96 | 0.205 | 0.184 | 0.019 | 0 | 0.718 |
| β-decay functional (q = 0) | North | 2020 | 345 | 0.199 | 0.198 | 0.011 | 0 | 1.000 |
| β-decay functional (q = 0) | North | 2021 | 90 | 0.165 | 0.192 | 0.020 | 0 | 0.729 |
| β-decay functional (q = 0) | North | 2022 | 87 | 0.127 | 0.123 | 0.013 | 0 | 0.585 |
| β-decay functional (q = 0) | North | 2023 | 427 | 0.193 | 0.198 | 0.010 | 0 | 1.000 |
| β-decay functional (q = 0) | North | 2024 | 48 | 0.134 | 0.149 | 0.022 | 0 | 0.667 |
| β-decay functional (q = 0) | Middle | 2019 | 240 | 0.139 | 0.147 | 0.010 | 0 | 0.550 |
| β-decay functional (q = 0) | Middle | 2020 | 272 | 0.135 | 0.140 | 0.008 | 0 | 0.545 |
| β-decay functional (q = 0) | Middle | 2022 | 80 | 0.176 | 0.236 | 0.026 | 0 | 0.690 |
| β-decay functional (q = 0) | Middle | 2023 | 252 | 0.154 | 0.133 | 0.008 | 0 | 0.443 |
| β-decay functional (q = 0) | Middle | 2024 | 256 | 0.152 | 0.164 | 0.010 | 0 | 0.690 |
| β-decay functional (q = 0) | South | 2019 | 144 | 0.066 | 0.104 | 0.009 | 0 | 0.457 |
| β-decay functional (q = 0) | South | 2020 | 144 | 0.101 | 0.106 | 0.009 | 0 | 0.448 |
| β-decay functional (q = 0) | South | 2021 | 144 | 0.097 | 0.114 | 0.010 | 0 | 0.410 |
| β-decay functional (q = 0) | South | 2022 | 648 | 0.200 | 0.184 | 0.007 | 0 | 0.776 |
| β-decay functional (q = 0) | South | 2023 | 420 | 0.122 | 0.141 | 0.007 | 0 | 0.653 |
| β-decay functional (q = 0) | South | 2024 | 408 | 0.143 | 0.200 | 0.010 | 0 | 1.000 |
| β-decay functional (q = 1) | North | 2019 | 96 | 0.294 | 0.209 | 0.021 | 0.019 | 0.989 |
| β-decay functional (q = 1) | North | 2020 | 345 | 0.318 | 0.210 | 0.011 | 0.011 | 1.000 |
| β-decay functional (q = 1) | North | 2021 | 90 | 0.216 | 0.151 | 0.016 | 0.006 | 0.642 |
| β-decay functional (q = 1) | North | 2022 | 87 | 0.284 | 0.200 | 0.021 | 0.002 | 0.936 |
| β-decay functional (q = 1) | North | 2023 | 427 | 0.288 | 0.238 | 0.012 | 0.013 | 1.000 |
| β-decay functional (q = 1) | North | 2024 | 48 | 0.477 | 0.258 | 0.037 | 0.097 | 0.980 |
| β-decay functional (q = 1) | Middle | 2019 | 240 | 0.263 | 0.180 | 0.012 | 0.011 | 0.808 |
| β-decay functional (q = 1) | Middle | 2020 | 272 | 0.291 | 0.197 | 0.012 | 0.002 | 0.908 |
| β-decay functional (q = 1) | Middle | 2022 | 80 | 0.281 | 0.167 | 0.019 | 0.021 | 0.782 |
| β-decay functional (q = 1) | Middle | 2023 | 252 | 0.244 | 0.169 | 0.011 | 0.013 | 0.754 |
| β-decay functional (q = 1) | Middle | 2024 | 256 | 0.294 | 0.198 | 0.012 | 0.012 | 0.838 |
| β-decay functional (q = 1) | South | 2019 | 144 | 0.271 | 0.203 | 0.017 | 0.011 | 0.789 |
| β-decay functional (q = 1) | South | 2020 | 144 | 0.173 | 0.144 | 0.012 | 0.006 | 0.658 |
| β-decay functional (q = 1) | South | 2021 | 144 | 0.219 | 0.169 | 0.014 | 0.009 | 0.801 |
| β-decay functional (q = 1) | South | 2022 | 648 | 0.365 | 0.247 | 0.010 | 0.006 | 0.999 |
| β-decay functional (q = 1) | South | 2023 | 420 | 0.296 | 0.181 | 0.009 | 0.004 | 0.877 |
| β-decay functional (q = 1) | South | 2024 | 408 | 0.257 | 0.206 | 0.010 | 0.007 | 1.000 |
| β-decay taxonomic (q = 0) | North | 2019 | 96 | 0.679 | 0.181 | 0.019 | 0.200 | 0.889 |
| β-decay taxonomic (q = 0) | North | 2020 | 345 | 0.683 | 0.171 | 0.009 | 0.200 | 1.000 |
| β-decay taxonomic (q = 0) | North | 2021 | 90 | 0.592 | 0.150 | 0.016 | 0.200 | 0.889 |
| β-decay taxonomic (q = 0) | North | 2022 | 87 | 0.588 | 0.185 | 0.020 | 0.000 | 1.000 |
| β-decay taxonomic (q = 0) | North | 2023 | 427 | 0.678 | 0.197 | 0.010 | 0.000 | 1.000 |
| β-decay taxonomic (q = 0) | North | 2024 | 48 | 0.563 | 0.163 | 0.024 | 0.250 | 0.800 |
| β-decay taxonomic (q = 0) | Middle | 2019 | 240 | 0.579 | 0.171 | 0.011 | 0.143 | 1.000 |
| β-decay taxonomic (q = 0) | Middle | 2020 | 272 | 0.507 | 0.199 | 0.012 | 0.000 | 1.000 |
| β-decay taxonomic (q = 0) | Middle | 2022 | 80 | 0.603 | 0.192 | 0.022 | 0.333 | 1.000 |
| β-decay taxonomic (q = 0) | Middle | 2023 | 252 | 0.459 | 0.158 | 0.010 | 0.125 | 0.857 |
| β-decay taxonomic (q = 0) | Middle | 2024 | 256 | 0.456 | 0.193 | 0.012 | 0.000 | 1.000 |
| β-decay taxonomic (q = 0) | South | 2019 | 144 | 0.545 | 0.137 | 0.011 | 0.200 | 0.818 |
| β-decay taxonomic (q = 0) | South | 2020 | 144 | 0.561 | 0.152 | 0.013 | 0.125 | 0.889 |
| β-decay taxonomic (q = 0) | South | 2021 | 144 | 0.562 | 0.159 | 0.013 | 0.000 | 0.917 |
| β-decay taxonomic (q = 0) | South | 2022 | 648 | 0.701 | 0.218 | 0.009 | 0.000 | 1.000 |
| β-decay taxonomic (q = 0) | South | 2023 | 420 | 0.564 | 0.164 | 0.008 | 0.000 | 1.000 |
| β-decay taxonomic (q = 0) | South | 2024 | 408 | 0.641 | 0.210 | 0.010 | 0.000 | 1.000 |
| β-decay taxonomic (q = 1) | North | 2019 | 96 | 0.619 | 0.300 | 0.031 | 0.032 | 0.997 |
| β-decay taxonomic (q = 1) | North | 2020 | 345 | 0.573 | 0.241 | 0.013 | 0.088 | 1.000 |
| β-decay taxonomic (q = 1) | North | 2021 | 90 | 0.380 | 0.209 | 0.022 | 0.018 | 0.926 |
| β-decay taxonomic (q = 1) | North | 2022 | 87 | 0.569 | 0.261 | 0.028 | 0.025 | 1.000 |
| β-decay taxonomic (q = 1) | North | 2023 | 427 | 0.594 | 0.266 | 0.013 | 0.051 | 1.000 |
| β-decay taxonomic (q = 1) | North | 2024 | 48 | 0.632 | 0.235 | 0.034 | 0.151 | 0.990 |
| β-decay taxonomic (q = 1) | Middle | 2019 | 240 | 0.447 | 0.218 | 0.014 | 0.051 | 1.000 |
| β-decay taxonomic (q = 1) | Middle | 2020 | 272 | 0.423 | 0.219 | 0.013 | 0.060 | 1.000 |
| β-decay taxonomic (q = 1) | Middle | 2022 | 80 | 0.473 | 0.216 | 0.024 | 0.073 | 1.000 |
| β-decay taxonomic (q = 1) | Middle | 2023 | 252 | 0.403 | 0.200 | 0.013 | 0.044 | 0.990 |
| β-decay taxonomic (q = 1) | Middle | 2024 | 256 | 0.471 | 0.224 | 0.014 | 0.034 | 1.000 |
| β-decay taxonomic (q = 1) | South | 2019 | 144 | 0.504 | 0.218 | 0.018 | 0.035 | 0.890 |
| β-decay taxonomic (q = 1) | South | 2020 | 144 | 0.440 | 0.203 | 0.017 | 0.038 | 0.854 |
| β-decay taxonomic (q = 1) | South | 2021 | 144 | 0.402 | 0.202 | 0.017 | 0.060 | 0.876 |
| β-decay taxonomic (q = 1) | South | 2022 | 648 | 0.645 | 0.285 | 0.011 | 0.035 | 1.000 |
| β-decay taxonomic (q = 1) | South | 2023 | 420 | 0.504 | 0.214 | 0.010 | 0.030 | 1.000 |
| β-decay taxonomic (q = 1) | South | 2024 | 408 | 0.532 | 0.261 | 0.013 | 0.053 | 1.000 |

**Table S7 | Combined taxonomic and functional SIMPER results**

SIMPER decomposition of Bray–Curtis dissimilarity between consecutive survey years, computed separately for taxonomic (species-level) and functional (trait-weighted) community matrices within each subregion. Component: species or trait; average, mean contribution to dissimilarity; overall, overall mean dissimilarity in the comparison; sd, standard deviation; ratio, average/sd; ava, avb, mean abundance (or trait value) of the component in the two compared years; ord, rank order; cusum, cumulative contribution; p, permutation p-value (999 permutations). Comparison indicates the two years being contrasted; Level indicates taxonomic or functional SIMPER.

| **Component** | **Average** | **Overall** | **SD** | **Ratio** | **ava** | **avb** | **Ord** | **Cusum** | **p** | **Comparison** | **Subregion** | **Level** |
| --- | --- | --- | --- | --- | --- | --- | --- | --- | --- | --- | --- | --- |
| Bodianus pulcher | 0.230 | 0.750 | 0.200 | 1.15 | 3337.50 | 1981.75 | 18 | 0.742 | 0.228 | 2017_vs_2018 | North | Taxonomic |
| Embiotoca jacksoni | 0.120 | 0.750 | 0.243 | 0.50 | 4343.37 | 112.59 | 4 | 0.794 | 0.010 | 2017_vs_2018 | North | Taxonomic |
| Hypsypops rubicundus | 0.107 | 0.750 | 0.110 | 0.97 | 995.37 | 666.85 | 1 | 0.907 | 0.079 | 2017_vs_2018 | North | Taxonomic |
| Oxyjulis californica | 0.053 | 0.750 | 0.062 | 0.87 | 486.09 | 312.76 | 9 | 0.610 | 0.426 | 2017_vs_2018 | North | Taxonomic |
| Paralabrax nebulifer | 0.046 | 0.750 | 0.137 | 0.34 | 12.32 | 1522.81 | 15 | 0.996 | 0.980 | 2017_vs_2018 | North | Taxonomic |
| Paralabrax clathratus | 0.039 | 0.750 | 0.050 | 0.78 | 235.27 | 409.96 | 3 | 0.681 | 0.960 | 2017_vs_2018 | North | Taxonomic |
| Anisotremus davidsonii | 0.033 | 0.750 | 0.139 | 0.24 | 2.75 | 996.21 | 25 | 1.000 | 0.960 | 2017_vs_2018 | North | Taxonomic |
| Sebastes serriceps | 0.026 | 0.750 | 0.065 | 0.40 | 0 | 360.10 | 22 | 0.999 | >0.999 | 2017_vs_2018 | North | Taxonomic |
| Chromis punctipinnis | 0.026 | 0.750 | 0.031 | 0.85 | 276.63 | 81.85 | 5 | 0.307 | 0.059 | 2017_vs_2018 | North | Taxonomic |
| Girella nigricans | 0.014 | 0.750 | 0.056 | 0.25 | 0 | 137.49 | 11 | 0.956 | 0.970 | 2017_vs_2018 | North | Taxonomic |
| Bodianus pulcher | 0.187 | 0.863 | 0.196 | 0.96 | 1981.75 | 344.57 | 11 | 0.553 | 0.416 | 2018_vs_2019 | North | Taxonomic |
| Sebastes serriceps | 0.084 | 0.863 | 0.163 | 0.51 | 360.10 | 658.32 | 45 | 0.983 | 0.158 | 2018_vs_2019 | North | Taxonomic |
| Hypsypops rubicundus | 0.072 | 0.863 | 0.103 | 0.70 | 666.85 | 169.46 | 23 | 0.819 | 0.782 | 2018_vs_2019 | North | Taxonomic |
| Oxyjulis californica | 0.067 | 0.863 | 0.104 | 0.65 | 312.76 | 318.55 | 9 | 0.398 | 0.228 | 2018_vs_2019 | North | Taxonomic |
| Rhacochilus vacca | 0.067 | 0.863 | 0.164 | 0.41 | 174.18 | 706.43 | 14 | 0.880 | 0.188 | 2018_vs_2019 | North | Taxonomic |
| Paralabrax clathratus | 0.066 | 0.863 | 0.087 | 0.76 | 409.96 | 165.16 | 3 | 0.476 | 0.257 | 2018_vs_2019 | North | Taxonomic |
| Paralabrax nebulifer | 0.066 | 0.863 | 0.167 | 0.40 | 1522.81 | 44.45 | 48 | 0.979 | 0.663 | 2018_vs_2019 | North | Taxonomic |
| Chromis punctipinnis | 0.058 | 0.863 | 0.144 | 0.41 | 81.85 | 781.56 | 5 | 0.217 | 0.129 | 2018_vs_2019 | North | Taxonomic |
| Anisotremus davidsonii | 0.040 | 0.863 | 0.161 | 0.25 | 996.21 | 0.36 | 49 | 0.994 | 0.644 | 2018_vs_2019 | North | Taxonomic |
| Embiotoca jacksoni | 0.028 | 0.863 | 0.049 | 0.57 | 112.59 | 150.77 | 4 | 0.629 | 0.257 | 2018_vs_2019 | North | Taxonomic |
| Paralabrax clathratus | 0.126 | 0.856 | 0.147 | 0.85 | 165.16 | 1028.60 | 9 | 0.531 | 0.178 | 2019_vs_2020 | North | Taxonomic |
| Bodianus pulcher | 0.122 | 0.856 | 0.124 | 0.99 | 344.57 | 906.04 | 11 | 0.611 | 0.406 | 2019_vs_2020 | North | Taxonomic |
| Chromis punctipinnis | 0.105 | 0.856 | 0.168 | 0.63 | 781.56 | 479.59 | 4 | 0.147 | 0.248 | 2019_vs_2020 | North | Taxonomic |
| Hypsypops rubicundus | 0.101 | 0.856 | 0.116 | 0.87 | 169.46 | 840.18 | 19 | 0.853 | 0.149 | 2019_vs_2020 | North | Taxonomic |
| Rhacochilus vacca | 0.068 | 0.856 | 0.152 | 0.45 | 706.43 | 126.67 | 51 | 0.904 | 0.178 | 2019_vs_2020 | North | Taxonomic |
| Embiotoca jacksoni | 0.055 | 0.856 | 0.079 | 0.70 | 150.77 | 367.12 | 6 | 0.676 | 0.386 | 2019_vs_2020 | North | Taxonomic |
| Oxyjulis californica | 0.054 | 0.856 | 0.099 | 0.55 | 318.55 | 173.05 | 1 | 0.413 | 0.188 | 2019_vs_2020 | North | Taxonomic |
| Girella nigricans | 0.052 | 0.856 | 0.096 | 0.54 | 0 | 676.07 | 15 | 0.923 | 0.713 | 2019_vs_2020 | North | Taxonomic |
| Sebastes serriceps | 0.045 | 0.856 | 0.132 | 0.34 | 658.32 | 0 | 23 | 0.978 | 0.119 | 2019_vs_2020 | North | Taxonomic |
| Sebastes atrovirens | 0.026 | 0.856 | 0.065 | 0.40 | 96.53 | 41.55 | 22 | 0.948 | 0.366 | 2019_vs_2020 | North | Taxonomic |
| Hypsypops rubicundus | 0.136 | 0.777 | 0.138 | 0.99 | 840.18 | 1633.48 | 15 | 0.883 | 0.089 | 2020_vs_2021 | North | Taxonomic |
| Chromis punctipinnis | 0.136 | 0.777 | 0.172 | 0.79 | 479.59 | 2089.15 | 9 | 0.175 | 0.050 | 2020_vs_2021 | North | Taxonomic |
| Paralabrax clathratus | 0.116 | 0.777 | 0.136 | 0.85 | 1028.60 | 1028.70 | 5 | 0.641 | 0.723 | 2020_vs_2021 | North | Taxonomic |
| Bodianus pulcher | 0.110 | 0.777 | 0.110 | 1.00 | 906.04 | 1142.56 | 12 | 0.703 | 0.812 | 2020_vs_2021 | North | Taxonomic |
| Girella nigricans | 0.048 | 0.777 | 0.084 | 0.57 | 676.07 | 248.06 | 11 | 0.940 | 0.980 | 2020_vs_2021 | North | Taxonomic |
| Embiotoca jacksoni | 0.048 | 0.777 | 0.072 | 0.66 | 367.12 | 169.49 | 6 | 0.764 | 0.871 | 2020_vs_2021 | North | Taxonomic |
| Halichoeres semicinctus | 0.043 | 0.777 | 0.068 | 0.63 | 103.69 | 883.96 | 1 | 0.350 | 0.040 | 2020_vs_2021 | North | Taxonomic |
| Oxyjulis californica | 0.026 | 0.777 | 0.060 | 0.43 | 173.05 | 92.43 | 4 | 0.499 | 0.921 | 2020_vs_2021 | North | Taxonomic |
| Medialuna californiensis | 0.023 | 0.777 | 0.035 | 0.67 | 75.07 | 380.98 | 22 | 0.972 | 0.069 | 2020_vs_2021 | North | Taxonomic |
| Caulolatilus princeps | 0.018 | 0.777 | 0.045 | 0.40 | 0 | 232.31 | 62 | 0.955 | 0.010 | 2020_vs_2021 | North | Taxonomic |
| Hypsypops rubicundus | 0.150 | 0.791 | 0.149 | 1.01 | 1633.48 | 1558.88 | 62 | 0.911 | 0.455 | 2021_vs_2022 | North | Taxonomic |
| Bodianus pulcher | 0.129 | 0.791 | 0.146 | 0.88 | 1142.56 | 1558.26 | 13 | 0.721 | 0.683 | 2021_vs_2022 | North | Taxonomic |
| Paralabrax clathratus | 0.120 | 0.791 | 0.150 | 0.80 | 1028.70 | 1112.22 | 1 | 0.647 | 0.426 | 2021_vs_2022 | North | Taxonomic |
| Chromis punctipinnis | 0.114 | 0.791 | 0.166 | 0.69 | 2089.15 | 383.07 | 9 | 0.190 | 0.010 | 2021_vs_2022 | North | Taxonomic |
| Caulolatilus princeps | 0.058 | 0.791 | 0.138 | 0.42 | 232.31 | 548.26 | 24 | 0.982 | 0.851 | 2021_vs_2022 | North | Taxonomic |
| Halichoeres semicinctus | 0.046 | 0.791 | 0.070 | 0.66 | 883.96 | 250.49 | 5 | 0.352 | 0.089 | 2021_vs_2022 | North | Taxonomic |
| Girella nigricans | 0.042 | 0.791 | 0.085 | 0.50 | 248.06 | 750.53 | 14 | 0.978 | 0.921 | 2021_vs_2022 | North | Taxonomic |
| Embiotoca jacksoni | 0.037 | 0.791 | 0.069 | 0.53 | 169.49 | 362.95 | 2 | 0.779 | 0.614 | 2021_vs_2022 | North | Taxonomic |
| Balistes polylepis | 0.026 | 0.791 | 0.099 | 0.26 | 219.98 | 443.20 | 59 | 1.000 | 0.564 | 2021_vs_2022 | North | Taxonomic |
| Oxyjulis californica | 0.025 | 0.791 | 0.037 | 0.68 | 92.43 | 290.93 | 4 | 0.503 | 0.802 | 2021_vs_2022 | North | Taxonomic |
| Bodianus pulcher | 0.155 | 0.803 | 0.167 | 0.93 | 1558.26 | 874.82 | 12 | 0.692 | 0.960 | 2022_vs_2023 | North | Taxonomic |
| Hypsypops rubicundus | 0.143 | 0.803 | 0.140 | 1.02 | 1558.88 | 510.13 | 2 | 0.895 | 0.010 | 2022_vs_2023 | North | Taxonomic |
| Paralabrax clathratus | 0.129 | 0.803 | 0.157 | 0.82 | 1112.22 | 313.31 | 1 | 0.617 | 0.010 | 2022_vs_2023 | North | Taxonomic |
| Chromis punctipinnis | 0.069 | 0.803 | 0.129 | 0.53 | 383.07 | 689.57 | 5 | 0.192 | 0.990 | 2022_vs_2023 | North | Taxonomic |
| Girella nigricans | 0.060 | 0.803 | 0.102 | 0.59 | 750.53 | 262.46 | 14 | 0.944 | 0.446 | 2022_vs_2023 | North | Taxonomic |
| Caulolatilus princeps | 0.058 | 0.803 | 0.158 | 0.37 | 548.26 | 21.19 | 74 | 0.957 | 0.010 | 2022_vs_2023 | North | Taxonomic |
| Embiotoca jacksoni | 0.047 | 0.803 | 0.090 | 0.53 | 362.95 | 222.26 | 13 | 0.764 | 0.960 | 2022_vs_2023 | North | Taxonomic |
| Oxyjulis californica | 0.035 | 0.803 | 0.050 | 0.70 | 290.93 | 110.52 | 4 | 0.531 | 0.099 | 2022_vs_2023 | North | Taxonomic |
| Halichoeres semicinctus | 0.023 | 0.803 | 0.047 | 0.49 | 250.49 | 49.86 | 9 | 0.371 | 0.040 | 2022_vs_2023 | North | Taxonomic |
| Balistes polylepis | 0.017 | 0.803 | 0.094 | 0.18 | 443.20 | 0 | 58 | 1.000 | 0.010 | 2022_vs_2023 | North | Taxonomic |
| Paralabrax clathratus | 0.207 | 0.871 | 0.236 | 0.88 | 313.31 | 2455.81 | 5 | 0.715 | 0.010 | 2023_vs_2024 | North | Taxonomic |
| Hypsypops rubicundus | 0.152 | 0.871 | 0.163 | 0.93 | 510.13 | 862.88 | 20 | 0.926 | 0.089 | 2023_vs_2024 | North | Taxonomic |
| Chromis punctipinnis | 0.136 | 0.871 | 0.170 | 0.80 | 689.57 | 890.51 | 4 | 0.237 | 0.129 | 2023_vs_2024 | North | Taxonomic |
| Bodianus pulcher | 0.128 | 0.871 | 0.158 | 0.81 | 874.82 | 190.14 | 3 | 0.815 | 0.970 | 2023_vs_2024 | North | Taxonomic |
| Oxyjulis californica | 0.087 | 0.871 | 0.135 | 0.64 | 110.52 | 261.95 | 1 | 0.568 | 0.020 | 2023_vs_2024 | North | Taxonomic |
| Embiotoca jacksoni | 0.037 | 0.871 | 0.101 | 0.37 | 222.26 | 64.05 | 6 | 0.858 | 0.911 | 2023_vs_2024 | North | Taxonomic |
| Girella nigricans | 0.035 | 0.871 | 0.079 | 0.45 | 262.46 | 42.58 | 14 | 0.962 | 0.713 | 2023_vs_2024 | North | Taxonomic |
| Halichoeres semicinctus | 0.013 | 0.871 | 0.024 | 0.51 | 49.86 | 24.12 | 9 | 0.412 | 0.287 | 2023_vs_2024 | North | Taxonomic |
| Rhacochilus toxotes | 0.011 | 0.871 | 0.067 | 0.17 | 116.50 | 0 | 48 | 0.996 | 0.743 | 2023_vs_2024 | North | Taxonomic |
| Hexagrammos decagrammus | 0.011 | 0.871 | 0.082 | 0.14 | 286.41 | 0 | 72 | 1.000 | 0.950 | 2023_vs_2024 | North | Taxonomic |
| Bodianus pulcher | 0.211 | 0.721 | 0.168 | 1.26 | 2089.37 | 505.19 | 4 | 0.784 | 0.277 | 2017_vs_2018 | Middle | Taxonomic |
| Chromis punctipinnis | 0.123 | 0.721 | 0.127 | 0.97 | 1013.78 | 144.57 | 5 | 0.293 | 0.208 | 2017_vs_2018 | Middle | Taxonomic |
| Girella nigricans | 0.107 | 0.721 | 0.152 | 0.71 | 998.39 | 102.86 | 15 | 0.975 | 0.485 | 2017_vs_2018 | Middle | Taxonomic |
| Hypsypops rubicundus | 0.066 | 0.721 | 0.053 | 1.24 | 553.41 | 66.76 | 6 | 0.939 | 0.079 | 2017_vs_2018 | Middle | Taxonomic |
| Paralabrax clathratus | 0.058 | 0.721 | 0.052 | 1.13 | 436.26 | 275.91 | 9 | 0.704 | 0.129 | 2017_vs_2018 | Middle | Taxonomic |
| Oxyjulis californica | 0.049 | 0.721 | 0.058 | 0.85 | 263.20 | 240.98 | 12 | 0.612 | 0.119 | 2017_vs_2018 | Middle | Taxonomic |
| Halichoeres semicinctus | 0.030 | 0.721 | 0.027 | 1.13 | 189.69 | 0 | 1 | 0.464 | 0.020 | 2017_vs_2018 | Middle | Taxonomic |
| Hermosilla azurea | 0.023 | 0.721 | 0.058 | 0.39 | 0 | 144.70 | 28 | 1.000 | 0.178 | 2017_vs_2018 | Middle | Taxonomic |
| Embiotoca jacksoni | 0.009 | 0.721 | 0.012 | 0.79 | 51.70 | 30.68 | 3 | 0.853 | 0.277 | 2017_vs_2018 | Middle | Taxonomic |
| Atherinopsidae spp | 0.009 | 0.721 | 0.046 | 0.20 | 101.09 | 0 | 31 | 0.995 | 0.168 | 2017_vs_2018 | Middle | Taxonomic |
| Bodianus pulcher | 0.276 | 0.821 | 0.221 | 1.25 | 505.19 | 6366.03 | 41 | 0.817 | 0.119 | 2018_vs_2019 | Middle | Taxonomic |
| Paralabrax clathratus | 0.211 | 0.821 | 0.186 | 1.13 | 275.91 | 2984.71 | 12 | 0.752 | 0.010 | 2018_vs_2019 | Middle | Taxonomic |
| Hypsypops rubicundus | 0.071 | 0.821 | 0.078 | 0.92 | 66.76 | 1026.59 | 30 | 0.962 | 0.109 | 2018_vs_2019 | Middle | Taxonomic |
| Girella nigricans | 0.060 | 0.821 | 0.111 | 0.54 | 102.86 | 1557.63 | 43 | 0.989 | 0.475 | 2018_vs_2019 | Middle | Taxonomic |
| Zapteryx exasperata | 0.053 | 0.821 | 0.178 | 0.30 | 0 | 1042.56 | 38 | 1.000 | 0.267 | 2018_vs_2019 | Middle | Taxonomic |
| Halichoeres semicinctus | 0.052 | 0.821 | 0.095 | 0.55 | 0 | 672.14 | 4 | 0.593 | 0.396 | 2018_vs_2019 | Middle | Taxonomic |
| Oxyjulis californica | 0.032 | 0.821 | 0.046 | 0.70 | 240.98 | 137.11 | 9 | 0.679 | 0.376 | 2018_vs_2019 | Middle | Taxonomic |
| Chromis punctipinnis | 0.020 | 0.821 | 0.024 | 0.84 | 144.57 | 171.77 | 5 | 0.336 | 0.366 | 2018_vs_2019 | Middle | Taxonomic |
| Hermosilla azurea | 0.015 | 0.821 | 0.044 | 0.34 | 144.70 | 0 | 24 | 1.000 | 0.446 | 2018_vs_2019 | Middle | Taxonomic |
| Medialuna californiensis | 0.012 | 0.821 | 0.036 | 0.33 | 0 | 396.15 | 31 | 0.998 | 0.376 | 2018_vs_2019 | Middle | Taxonomic |
| Bodianus pulcher | 0.277 | 0.695 | 0.221 | 1.25 | 6366.03 | 19334.40 | 2 | 0.820 | 0.693 | 2019_vs_2020 | Middle | Taxonomic |
| Paralabrax clathratus | 0.136 | 0.695 | 0.139 | 0.98 | 2984.71 | 5909.41 | 12 | 0.759 | 0.287 | 2019_vs_2020 | Middle | Taxonomic |
| Hypsypops rubicundus | 0.062 | 0.695 | 0.078 | 0.79 | 1026.59 | 1843.56 | 3 | 0.940 | 0.307 | 2019_vs_2020 | Middle | Taxonomic |
| Girella nigricans | 0.052 | 0.695 | 0.081 | 0.65 | 1557.63 | 1602.86 | 13 | 0.982 | 0.436 | 2019_vs_2020 | Middle | Taxonomic |
| Halichoeres semicinctus | 0.043 | 0.695 | 0.058 | 0.73 | 672.14 | 867.04 | 4 | 0.595 | 0.208 | 2019_vs_2020 | Middle | Taxonomic |
| Zapteryx exasperata | 0.034 | 0.695 | 0.104 | 0.33 | 1042.56 | 781.92 | 37 | 1.000 | 0.396 | 2019_vs_2020 | Middle | Taxonomic |
| Chromis punctipinnis | 0.022 | 0.695 | 0.025 | 0.86 | 171.77 | 1599.07 | 5 | 0.399 | 0.574 | 2019_vs_2020 | Middle | Taxonomic |
| Embiotoca jacksoni | 0.015 | 0.695 | 0.025 | 0.60 | 0 | 452.31 | 41 | 0.869 | 0.050 | 2019_vs_2020 | Middle | Taxonomic |
| Oxyjulis californica | 0.013 | 0.695 | 0.021 | 0.61 | 137.11 | 255.36 | 9 | 0.683 | 0.347 | 2019_vs_2020 | Middle | Taxonomic |
| Stereolepis gigas | 0.012 | 0.695 | 0.048 | 0.25 | 0 | 843.09 | 51 | 1.000 | 0.495 | 2019_vs_2020 | Middle | Taxonomic |
| Bodianus pulcher | 0.277 | 0.758 | 0.220 | 1.26 | 19334.40 | 3157.39 | 2 | 0.815 | 0.772 | 2020_vs_2022 | Middle | Taxonomic |
| Paralabrax clathratus | 0.162 | 0.758 | 0.167 | 0.97 | 5909.41 | 1695.17 | 9 | 0.761 | 0.129 | 2020_vs_2022 | Middle | Taxonomic |
| Embiotoca jacksoni | 0.070 | 0.758 | 0.087 | 0.81 | 452.31 | 2290.52 | 12 | 0.860 | 0.059 | 2020_vs_2022 | Middle | Taxonomic |
| Hypsypops rubicundus | 0.068 | 0.758 | 0.090 | 0.76 | 1843.56 | 451.33 | 3 | 0.944 | 0.307 | 2020_vs_2022 | Middle | Taxonomic |
| Halichoeres semicinctus | 0.040 | 0.758 | 0.058 | 0.70 | 867.04 | 33.23 | 4 | 0.579 | 0.139 | 2020_vs_2022 | Middle | Taxonomic |
| Girella nigricans | 0.034 | 0.758 | 0.058 | 0.59 | 1602.86 | 0 | 41 | 0.983 | 0.455 | 2020_vs_2022 | Middle | Taxonomic |
| Medialuna californiensis | 0.027 | 0.758 | 0.059 | 0.45 | 78.61 | 789.10 | 22 | 0.996 | 0.248 | 2020_vs_2022 | Middle | Taxonomic |
| Chromis punctipinnis | 0.023 | 0.758 | 0.026 | 0.87 | 1599.07 | 141.05 | 5 | 0.365 | 0.743 | 2020_vs_2022 | Middle | Taxonomic |
| Oxyjulis californica | 0.014 | 0.758 | 0.023 | 0.58 | 255.36 | 84.35 | 6 | 0.672 | 0.297 | 2020_vs_2022 | Middle | Taxonomic |
| Stereolepis gigas | 0.013 | 0.758 | 0.051 | 0.26 | 843.09 | 0 | 51 | 1.000 | 0.307 | 2020_vs_2022 | Middle | Taxonomic |
| Bodianus pulcher | 0.211 | 0.761 | 0.187 | 1.13 | 3157.39 | 2357.34 | 12 | 0.790 | 0.455 | 2022_vs_2023 | Middle | Taxonomic |
| Embiotoca jacksoni | 0.112 | 0.761 | 0.122 | 0.92 | 2290.52 | 173.54 | 9 | 0.869 | 0.020 | 2022_vs_2023 | Middle | Taxonomic |
| Paralabrax clathratus | 0.112 | 0.761 | 0.114 | 0.98 | 1695.17 | 666.61 | 1 | 0.710 | 0.079 | 2022_vs_2023 | Middle | Taxonomic |
| Chromis punctipinnis | 0.106 | 0.761 | 0.114 | 0.93 | 141.05 | 1469.26 | 5 | 0.277 | 0.842 | 2022_vs_2023 | Middle | Taxonomic |
| Girella nigricans | 0.061 | 0.761 | 0.110 | 0.56 | 0 | 669.18 | 14 | 0.994 | 0.822 | 2022_vs_2023 | Middle | Taxonomic |
| Hypsypops rubicundus | 0.060 | 0.761 | 0.063 | 0.96 | 451.33 | 642.36 | 28 | 0.966 | 0.426 | 2022_vs_2023 | Middle | Taxonomic |
| Medialuna californiensis | 0.040 | 0.761 | 0.089 | 0.46 | 789.10 | 0 | 21 | 0.999 | 0.109 | 2022_vs_2023 | Middle | Taxonomic |
| Oxyjulis californica | 0.021 | 0.761 | 0.029 | 0.74 | 84.35 | 268.05 | 4 | 0.571 | 0.723 | 2022_vs_2023 | Middle | Taxonomic |
| Rhinobatos productus | 0.012 | 0.761 | 0.050 | 0.24 | 0 | 159.30 | 24 | 1.000 | 0.723 | 2022_vs_2023 | Middle | Taxonomic |
| Halichoeres semicinctus | 0.010 | 0.761 | 0.021 | 0.49 | 33.23 | 89.84 | 6 | 0.424 | 0.693 | 2022_vs_2023 | Middle | Taxonomic |
| Bodianus pulcher | 0.175 | 0.741 | 0.170 | 1.03 | 2357.34 | 2070.77 | 12 | 0.700 | 0.624 | 2023_vs_2024 | Middle | Taxonomic |
| Chromis punctipinnis | 0.112 | 0.741 | 0.107 | 1.05 | 1469.26 | 874.09 | 5 | 0.235 | 0.158 | 2023_vs_2024 | Middle | Taxonomic |
| Rhacochilus vacca | 0.082 | 0.741 | 0.180 | 0.46 | 0 | 2374.54 | 13 | 0.949 | 0.307 | 2023_vs_2024 | Middle | Taxonomic |
| Paralabrax clathratus | 0.078 | 0.741 | 0.109 | 0.72 | 666.61 | 871.36 | 4 | 0.603 | 0.584 | 2023_vs_2024 | Middle | Taxonomic |
| Girella nigricans | 0.072 | 0.741 | 0.107 | 0.67 | 669.18 | 327.52 | 28 | 0.966 | 0.218 | 2023_vs_2024 | Middle | Taxonomic |
| Hypsypops rubicundus | 0.057 | 0.741 | 0.069 | 0.83 | 642.36 | 683.82 | 3 | 0.907 | 0.406 | 2023_vs_2024 | Middle | Taxonomic |
| Tetronarce californica | 0.046 | 0.741 | 0.181 | 0.26 | 0 | 2684.35 | 78 | 1.000 | 0.653 | 2023_vs_2024 | Middle | Taxonomic |
| Halichoeres semicinctus | 0.028 | 0.741 | 0.036 | 0.78 | 89.84 | 349.70 | 1 | 0.386 | 0.089 | 2023_vs_2024 | Middle | Taxonomic |
| Oxyjulis californica | 0.022 | 0.741 | 0.028 | 0.78 | 268.05 | 122.31 | 11 | 0.497 | 0.337 | 2023_vs_2024 | Middle | Taxonomic |
| Embiotoca jacksoni | 0.016 | 0.741 | 0.022 | 0.73 | 173.54 | 116.24 | 9 | 0.777 | 0.426 | 2023_vs_2024 | Middle | Taxonomic |
| Bodianus pulcher | 0.145 | 0.758 | 0.123 | 1.17 | 2100.63 | 4940.68 | 23 | 0.713 | 0.109 | 2017_vs_2018 | South | Taxonomic |
| Paralabrax clathratus | 0.144 | 0.758 | 0.140 | 1.03 | 2681.54 | 5662.42 | 9 | 0.646 | 0.455 | 2017_vs_2018 | South | Taxonomic |
| Zapteryx exasperata | 0.122 | 0.758 | 0.221 | 0.55 | 0 | 6946.11 | 36 | 1.000 | 0.020 | 2017_vs_2018 | South | Taxonomic |
| Hypsypops rubicundus | 0.078 | 0.758 | 0.129 | 0.61 | 593.88 | 3938.93 | 39 | 0.882 | 0.109 | 2017_vs_2018 | South | Taxonomic |
| Anisotremus davidsonii | 0.051 | 0.758 | 0.140 | 0.37 | 292.63 | 1138.06 | 13 | 1.000 | 0.168 | 2017_vs_2018 | South | Taxonomic |
| Paralabrax nebulifer | 0.048 | 0.758 | 0.075 | 0.63 | 700.33 | 728.48 | 38 | 0.995 | 0.139 | 2017_vs_2018 | South | Taxonomic |
| Girella nigricans | 0.031 | 0.758 | 0.045 | 0.70 | 886.00 | 190.90 | 29 | 0.955 | >0.999 | 2017_vs_2018 | South | Taxonomic |
| Halichoeres semicinctus | 0.026 | 0.758 | 0.038 | 0.68 | 406.79 | 608.55 | 4 | 0.381 | 0.851 | 2017_vs_2018 | South | Taxonomic |
| Urobatis concentricus | 0.023 | 0.758 | 0.071 | 0.33 | 0 | 320.98 | 34 | 1.000 | 0.010 | 2017_vs_2018 | South | Taxonomic |
| Chromis punctipinnis | 0.023 | 0.758 | 0.030 | 0.77 | 60.18 | 1074.75 | 5 | 0.191 | 0.030 | 2017_vs_2018 | South | Taxonomic |
| Bodianus pulcher | 0.151 | 0.790 | 0.133 | 1.14 | 4940.68 | 2888.17 | 12 | 0.717 | 0.921 | 2018_vs_2019 | South | Taxonomic |
| Paralabrax clathratus | 0.145 | 0.790 | 0.142 | 1.02 | 5662.42 | 1445.04 | 9 | 0.643 | 0.020 | 2018_vs_2019 | South | Taxonomic |
| Zapteryx exasperata | 0.130 | 0.790 | 0.214 | 0.61 | 6946.11 | 804.44 | 34 | 1.000 | 0.030 | 2018_vs_2019 | South | Taxonomic |
| Hypsypops rubicundus | 0.081 | 0.790 | 0.125 | 0.65 | 3938.93 | 990.34 | 1 | 0.895 | 0.416 | 2018_vs_2019 | South | Taxonomic |
| Girella nigricans | 0.058 | 0.790 | 0.140 | 0.42 | 190.90 | 3213.72 | 51 | 0.961 | >0.999 | 2018_vs_2019 | South | Taxonomic |
| Anisotremus davidsonii | 0.041 | 0.790 | 0.139 | 0.29 | 1138.06 | 0 | 6 | 1.000 | 0.020 | 2018_vs_2019 | South | Taxonomic |
| Paralabrax nebulifer | 0.039 | 0.790 | 0.074 | 0.53 | 728.48 | 122.47 | 38 | 0.998 | 0.010 | 2018_vs_2019 | South | Taxonomic |
| Halichoeres semicinctus | 0.034 | 0.790 | 0.036 | 0.93 | 608.55 | 1123.64 | 4 | 0.375 | 0.990 | 2018_vs_2019 | South | Taxonomic |
| Chromis punctipinnis | 0.027 | 0.790 | 0.029 | 0.96 | 1074.75 | 413.90 | 5 | 0.191 | 0.891 | 2018_vs_2019 | South | Taxonomic |
| Urobatis concentricus | 0.022 | 0.790 | 0.068 | 0.33 | 320.98 | 0 | 32 | 1.000 | 0.010 | 2018_vs_2019 | South | Taxonomic |
| Bodianus pulcher | 0.192 | 0.760 | 0.176 | 1.09 | 2888.17 | 6134.79 | 9 | 0.742 | 0.406 | 2019_vs_2020 | South | Taxonomic |
| Paralabrax clathratus | 0.161 | 0.760 | 0.158 | 1.01 | 1445.04 | 6257.60 | 12 | 0.660 | 0.030 | 2019_vs_2020 | South | Taxonomic |
| Zapteryx exasperata | 0.079 | 0.760 | 0.141 | 0.56 | 804.44 | 2120.52 | 32 | 1.000 | 0.089 | 2019_vs_2020 | South | Taxonomic |
| Girella nigricans | 0.070 | 0.760 | 0.150 | 0.47 | 3213.72 | 342.84 | 6 | 0.958 | 0.990 | 2019_vs_2020 | South | Taxonomic |
| Hypsypops rubicundus | 0.063 | 0.760 | 0.072 | 0.86 | 990.34 | 1224.27 | 29 | 0.902 | 0.040 | 2019_vs_2020 | South | Taxonomic |
| Halichoeres semicinctus | 0.040 | 0.760 | 0.038 | 1.07 | 1123.64 | 1061.96 | 4 | 0.463 | 0.960 | 2019_vs_2020 | South | Taxonomic |
| Anisotremus davidsonii | 0.030 | 0.760 | 0.065 | 0.46 | 0 | 1365.36 | 36 | 1.000 | 0.020 | 2019_vs_2020 | South | Taxonomic |
| Urobatis halleri | 0.028 | 0.760 | 0.072 | 0.40 | 401.21 | 436.40 | 46 | 1.000 | 0.921 | 2019_vs_2020 | South | Taxonomic |
| Heterodontus francisci | 0.023 | 0.760 | 0.077 | 0.29 | 0 | 945.83 | 14 | 1.000 | 0.030 | 2019_vs_2020 | South | Taxonomic |
| Chromis punctipinnis | 0.020 | 0.760 | 0.027 | 0.72 | 413.90 | 324.50 | 5 | 0.252 | 0.970 | 2019_vs_2020 | South | Taxonomic |
| Bodianus pulcher | 0.203 | 0.771 | 0.175 | 1.16 | 6134.79 | 1582.13 | 2 | 0.761 | 0.317 | 2020_vs_2021 | South | Taxonomic |
| Paralabrax clathratus | 0.175 | 0.771 | 0.165 | 1.06 | 6257.60 | 1180.72 | 9 | 0.704 | 0.228 | 2020_vs_2021 | South | Taxonomic |
| Zapteryx exasperata | 0.088 | 0.771 | 0.157 | 0.56 | 2120.52 | 370.52 | 34 | 1.000 | 0.248 | 2020_vs_2021 | South | Taxonomic |
| Hypsypops rubicundus | 0.076 | 0.771 | 0.084 | 0.91 | 1224.27 | 122.23 | 13 | 0.911 | 0.010 | 2020_vs_2021 | South | Taxonomic |
| Halichoeres semicinctus | 0.044 | 0.771 | 0.036 | 1.24 | 1061.96 | 28.55 | 4 | 0.491 | 0.010 | 2020_vs_2021 | South | Taxonomic |
| Heterodontus francisci | 0.041 | 0.771 | 0.100 | 0.41 | 945.83 | 320.21 | 14 | 1.000 | 0.653 | 2020_vs_2021 | South | Taxonomic |
| Anisotremus davidsonii | 0.034 | 0.771 | 0.071 | 0.48 | 1365.36 | 0 | 11 | 1.000 | 0.446 | 2020_vs_2021 | South | Taxonomic |
| Paralabrax nebulifer | 0.020 | 0.771 | 0.033 | 0.62 | 143.68 | 240.57 | 21 | 0.998 | 0.683 | 2020_vs_2021 | South | Taxonomic |
| Caulolatilus princeps | 0.020 | 0.771 | 0.064 | 0.31 | 113.16 | 169.74 | 1 | 0.984 | 0.248 | 2020_vs_2021 | South | Taxonomic |
| Urobatis halleri | 0.017 | 0.771 | 0.039 | 0.44 | 436.40 | 0 | 47 | 1.000 | 0.307 | 2020_vs_2021 | South | Taxonomic |
| Bodianus pulcher | 0.213 | 0.773 | 0.176 | 1.21 | 1582.13 | 1389.54 | 9 | 0.706 | 0.257 | 2021_vs_2022 | South | Taxonomic |
| Paralabrax clathratus | 0.191 | 0.773 | 0.164 | 1.16 | 1180.72 | 1034.39 | 2 | 0.656 | 0.158 | 2021_vs_2022 | South | Taxonomic |
| Heterodontus francisci | 0.052 | 0.773 | 0.149 | 0.35 | 320.21 | 346.21 | 72 | 1.000 | 0.396 | 2021_vs_2022 | South | Taxonomic |
| Halichoeres semicinctus | 0.051 | 0.773 | 0.082 | 0.62 | 28.55 | 518.38 | 4 | 0.523 | >0.999 | 2021_vs_2022 | South | Taxonomic |
| Hypsypops rubicundus | 0.039 | 0.773 | 0.042 | 0.93 | 122.23 | 303.26 | 12 | 0.860 | 0.990 | 2021_vs_2022 | South | Taxonomic |
| Paralabrax nebulifer | 0.036 | 0.773 | 0.051 | 0.70 | 240.57 | 53.78 | 15 | 0.978 | 0.020 | 2021_vs_2022 | South | Taxonomic |
| Zapteryx exasperata | 0.034 | 0.773 | 0.117 | 0.29 | 370.52 | 0 | 26 | 1.000 | 0.089 | 2021_vs_2022 | South | Taxonomic |
| Embiotoca jacksoni | 0.025 | 0.773 | 0.058 | 0.43 | 24.65 | 143.42 | 18 | 0.752 | 0.960 | 2021_vs_2022 | South | Taxonomic |
| Girella nigricans | 0.024 | 0.773 | 0.045 | 0.54 | 98.95 | 83.42 | 13 | 0.923 | 0.257 | 2021_vs_2022 | South | Taxonomic |
| Chromis punctipinnis | 0.019 | 0.773 | 0.037 | 0.51 | 27.36 | 98.12 | 5 | 0.275 | 0.941 | 2021_vs_2022 | South | Taxonomic |
| Paralabrax clathratus | 0.214 | 0.779 | 0.187 | 1.15 | 1034.39 | 2514.17 | 9 | 0.670 | 0.020 | 2022_vs_2023 | South | Taxonomic |
| Bodianus pulcher | 0.170 | 0.779 | 0.146 | 1.16 | 1389.54 | 1473.16 | 12 | 0.724 | 0.822 | 2022_vs_2023 | South | Taxonomic |
| Halichoeres semicinctus | 0.083 | 0.779 | 0.106 | 0.78 | 518.38 | 543.62 | 5 | 0.494 | 0.149 | 2022_vs_2023 | South | Taxonomic |
| Hypsypops rubicundus | 0.054 | 0.779 | 0.056 | 0.97 | 303.26 | 479.52 | 29 | 0.880 | 0.673 | 2022_vs_2023 | South | Taxonomic |
| Girella nigricans | 0.042 | 0.779 | 0.099 | 0.43 | 83.42 | 322.49 | 18 | 0.932 | 0.010 | 2022_vs_2023 | South | Taxonomic |
| Anisotremus davidsonii | 0.042 | 0.779 | 0.121 | 0.35 | 19.76 | 706.47 | 70 | 0.996 | 0.010 | 2022_vs_2023 | South | Taxonomic |
| Chromis punctipinnis | 0.032 | 0.779 | 0.071 | 0.46 | 98.12 | 363.51 | 4 | 0.275 | 0.594 | 2022_vs_2023 | South | Taxonomic |
| Embiotoca jacksoni | 0.029 | 0.779 | 0.063 | 0.46 | 143.42 | 85.08 | 23 | 0.778 | 0.990 | 2022_vs_2023 | South | Taxonomic |
| Heterodontus francisci | 0.018 | 0.779 | 0.097 | 0.19 | 346.21 | 13.30 | 76 | 1.000 | >0.999 | 2022_vs_2023 | South | Taxonomic |
| Calamus brachysomus | 0.015 | 0.779 | 0.055 | 0.27 | 88.61 | 0 | 53 | 1.000 | >0.999 | 2022_vs_2023 | South | Taxonomic |
| Paralabrax clathratus | 0.214 | 0.720 | 0.170 | 1.26 | 2514.17 | 1923.29 | 9 | 0.640 | 0.772 | 2023_vs_2024 | South | Taxonomic |
| Bodianus pulcher | 0.133 | 0.720 | 0.127 | 1.04 | 1473.16 | 1009.77 | 12 | 0.695 | 0.218 | 2023_vs_2024 | South | Taxonomic |
| Halichoeres semicinctus | 0.064 | 0.720 | 0.087 | 0.73 | 543.62 | 560.55 | 5 | 0.483 | 0.119 | 2023_vs_2024 | South | Taxonomic |
| Hypsypops rubicundus | 0.050 | 0.720 | 0.052 | 0.96 | 479.52 | 518.80 | 51 | 0.897 | 0.426 | 2023_vs_2024 | South | Taxonomic |
| Girella nigricans | 0.039 | 0.720 | 0.086 | 0.46 | 322.49 | 162.33 | 61 | 0.955 | 0.802 | 2023_vs_2024 | South | Taxonomic |
| Rhinobatos productus | 0.039 | 0.720 | 0.076 | 0.51 | 63.38 | 560.76 | 48 | 1.000 | 0.010 | 2023_vs_2024 | South | Taxonomic |
| Anisotremus davidsonii | 0.038 | 0.720 | 0.111 | 0.34 | 706.47 | 25.57 | 70 | 0.999 | 0.950 | 2023_vs_2024 | South | Taxonomic |
| Chromis punctipinnis | 0.035 | 0.720 | 0.074 | 0.47 | 363.51 | 299.86 | 4 | 0.298 | 0.614 | 2023_vs_2024 | South | Taxonomic |
| Urobatis halleri | 0.033 | 0.720 | 0.101 | 0.33 | 0 | 431.84 | 44 | 1.000 | 0.010 | 2023_vs_2024 | South | Taxonomic |
| Embiotoca jacksoni | 0.017 | 0.720 | 0.041 | 0.42 | 85.08 | 68.97 | 28 | 0.749 | 0.584 | 2023_vs_2024 | South | Taxonomic |
| a | 0.972 | 3.020 | 18.714 | 0.05 | 0.21 | -0.14 | 3 | 0.572 | 0.168 | 2017_vs_2018 | North | Functional |
| b | 0.757 | 3.020 | 15.142 | 0.05 | 0.01 | 0.23 | 5 | 0.721 | 0.208 | 2017_vs_2018 | North | Functional |
| Fishing_Vulnerability | 0.449 | 3.020 | 7.723 | 0.06 | 0.24 | 0.35 | 6 | 0.971 | 0.376 | 2017_vs_2018 | North | Functional |
| FecundityMax | 0.383 | 3.020 | 4.226 | 0.09 | -0.20 | 0.24 | 4 | 1.000 | 0.158 | 2017_vs_2018 | North | Functional |
| tm | 0.371 | 3.020 | 5.123 | 0.07 | 0.05 | 0.14 | 1 | 1.024 | 0.198 | 2017_vs_2018 | North | Functional |
| MaxSizeTL | 0.161 | 3.020 | 2.134 | 0.08 | 0.02 | 0.20 | 2 | 0.322 | 0.446 | 2017_vs_2018 | North | Functional |
| Trophic_level | -0.073 | 3.020 | 2.018 | -0.04 | -0.09 | 0.00 | 7 | 0.848 | 0.871 | 2017_vs_2018 | North | Functional |
| tm | 0.109 | 0.112 | 1.823 | 0.06 | 0.14 | -0.15 | 7 | 2.358 | 0.188 | 2018_vs_2019 | North | Functional |
| MaxSizeTL | 0.095 | 0.112 | 2.689 | 0.04 | 0.20 | -0.15 | 6 | 0.977 | 0.347 | 2018_vs_2019 | North | Functional |
| b | 0.055 | 0.112 | 3.282 | 0.02 | 0.23 | 0.20 | 3 | 2.311 | 0.257 | 2018_vs_2019 | North | Functional |
| a | 0.018 | 0.112 | 5.020 | 0.00 | -0.14 | -0.14 | 1 | 1.822 | 0.277 | 2018_vs_2019 | North | Functional |
| Fishing_Vulnerability | 0.005 | 0.112 | 3.885 | 0.00 | 0.35 | -0.01 | 5 | 2.516 | 0.465 | 2018_vs_2019 | North | Functional |
| FecundityMax | -0.018 | 0.112 | 5.586 | -0.00 | 0.24 | -0.12 | 4 | 1.000 | 0.455 | 2018_vs_2019 | North | Functional |
| Trophic_level | -0.152 | 0.112 | 3.900 | -0.04 | 0.00 | -0.15 | 2 | 2.470 | 0.822 | 2018_vs_2019 | North | Functional |
| Trophic_level | 0.283 | 0.099 | 5.656 | 0.05 | -0.15 | -0.28 | 5 | 6.312 | 0.099 | 2019_vs_2020 | North | Functional |
| FecundityMax | 0.182 | 0.099 | 6.350 | 0.03 | -0.12 | 0.08 | 3 | 1.000 | 0.327 | 2019_vs_2020 | North | Functional |
| MaxSizeTL | 0.110 | 0.099 | 2.961 | 0.04 | -0.15 | -0.10 | 4 | 2.852 | 0.376 | 2019_vs_2020 | North | Functional |
| Fishing_Vulnerability | 0.052 | 0.099 | 5.526 | 0.01 | -0.01 | 0.27 | 2 | 5.164 | 0.178 | 2019_vs_2020 | North | Functional |
| a | -0.114 | 0.099 | 2.869 | -0.04 | -0.14 | 0.16 | 7 | 4.689 | 0.525 | 2019_vs_2020 | North | Functional |
| tm | -0.145 | 0.099 | 2.278 | -0.06 | -0.15 | -0.11 | 6 | 3.701 | 0.347 | 2019_vs_2020 | North | Functional |
| b | -0.268 | 0.099 | 2.282 | -0.12 | 0.20 | 0.05 | 1 | 5.794 | 0.653 | 2019_vs_2020 | North | Functional |
| FecundityMax | 0.163 | -0.039 | 1.880 | 0.09 | 0.08 | -0.06 | 5 | 1.000 | 0.426 | 2020_vs_2021 | North | Functional |
| b | 0.045 | -0.039 | 3.885 | 0.01 | 0.05 | -0.14 | 1 | -5.385 | 0.644 | 2020_vs_2021 | North | Functional |
| MaxSizeTL | 0.005 | -0.039 | 3.380 | 0.00 | -0.10 | -0.07 | 7 | -4.118 | 0.653 | 2020_vs_2021 | North | Functional |
| Trophic_level | -0.011 | -0.039 | 5.017 | -0.00 | -0.28 | -0.24 | 4 | -5.111 | 0.446 | 2020_vs_2021 | North | Functional |
| a | -0.040 | -0.039 | 4.221 | -0.01 | 0.16 | 0.14 | 3 | -5.250 | 0.604 | 2020_vs_2021 | North | Functional |
| tm | -0.086 | -0.039 | 3.689 | -0.02 | -0.11 | 0.05 | 6 | -1.917 | 0.208 | 2020_vs_2021 | North | Functional |
| Fishing_Vulnerability | -0.115 | -0.039 | 3.872 | -0.03 | 0.27 | 0.28 | 2 | -4.088 | 0.257 | 2020_vs_2021 | North | Functional |
| FecundityMax | 1.054 | 4.714 | 17.902 | 0.06 | -0.06 | 0.15 | 5 | 1.000 | 0.079 | 2021_vs_2022 | North | Functional |
| a | 0.808 | 4.714 | 9.424 | 0.09 | 0.14 | 0.02 | 2 | 0.395 | 0.050 | 2021_vs_2022 | North | Functional |
| b | 0.730 | 4.714 | 11.551 | 0.06 | -0.14 | 0.05 | 3 | 0.550 | 0.129 | 2021_vs_2022 | North | Functional |
| MaxSizeTL | 0.679 | 4.714 | 8.896 | 0.08 | -0.07 | 0.06 | 7 | 0.224 | 0.079 | 2021_vs_2022 | North | Functional |
| tm | 0.652 | 4.714 | 12.796 | 0.05 | 0.05 | 0.06 | 4 | 0.934 | 0.079 | 2021_vs_2022 | North | Functional |
| Trophic_level | 0.480 | 4.714 | 7.427 | 0.06 | -0.24 | -0.12 | 1 | 0.694 | 0.149 | 2021_vs_2022 | North | Functional |
| Fishing_Vulnerability | 0.312 | 4.714 | 5.428 | 0.06 | 0.28 | 0.49 | 6 | 0.832 | 0.069 | 2021_vs_2022 | North | Functional |
| FecundityMax | 0.399 | 0.771 | 6.300 | 0.06 | 0.15 | -0.04 | 2 | 1.000 | 0.139 | 2022_vs_2023 | North | Functional |
| Trophic_level | 0.241 | 0.771 | 12.939 | 0.02 | -0.12 | -0.26 | 6 | 1.231 | 0.297 | 2022_vs_2023 | North | Functional |
| MaxSizeTL | 0.211 | 0.771 | 19.302 | 0.01 | 0.06 | -0.05 | 7 | 0.517 | 0.277 | 2022_vs_2023 | North | Functional |
| tm | 0.097 | 0.771 | 5.840 | 0.02 | 0.06 | 0.04 | 3 | 1.215 | 0.139 | 2022_vs_2023 | North | Functional |
| Fishing_Vulnerability | 0.094 | 0.771 | 15.455 | 0.01 | 0.49 | 0.31 | 5 | 1.354 | 0.277 | 2022_vs_2023 | North | Functional |
| b | -0.107 | 0.771 | 22.273 | -0.00 | 0.05 | -0.00 | 1 | 1.105 | 0.426 | 2022_vs_2023 | North | Functional |
| a | -0.166 | 0.771 | 26.257 | -0.01 | 0.02 | 0.18 | 4 | 0.831 | 0.317 | 2022_vs_2023 | North | Functional |
| a | -0.086 | -1.509 | 1.241 | -0.07 | 0.18 | -0.37 | 6 | 0.118 | 0.624 | 2023_vs_2024 | North | Functional |
| tm | -0.092 | -1.509 | 1.216 | -0.08 | 0.04 | -0.41 | 4 | 0.740 | 0.515 | 2023_vs_2024 | North | Functional |
| b | -0.142 | -1.509 | 3.035 | -0.05 | -0.00 | 0.50 | 3 | 0.212 | 0.564 | 2023_vs_2024 | North | Functional |
| MaxSizeTL | -0.189 | -1.509 | 2.591 | -0.07 | -0.05 | -0.39 | 2 | 0.057 | 0.752 | 2023_vs_2024 | North | Functional |
| FecundityMax | -0.293 | -1.509 | 6.896 | -0.04 | -0.04 | 0.12 | 5 | 1.000 | 0.792 | 2023_vs_2024 | North | Functional |
| Trophic_level | -0.316 | -1.509 | 3.984 | -0.08 | -0.26 | -0.35 | 1 | 0.337 | 0.772 | 2023_vs_2024 | North | Functional |
| Fishing_Vulnerability | -0.392 | -1.509 | 5.377 | -0.07 | 0.31 | -0.18 | 7 | 0.531 | 0.743 | 2023_vs_2024 | North | Functional |
| FecundityMax | -0.159 | -4.198 | 5.151 | -0.03 | -0.14 | 0.03 | 4 | 1.000 | 0.812 | 2017_vs_2018 | Middle | Functional |
| MaxSizeTL | -0.372 | -4.198 | 5.357 | -0.07 | 0.02 | 0.05 | 7 | 0.038 | 0.683 | 2017_vs_2018 | Middle | Functional |
| tm | -0.454 | -4.198 | 6.692 | -0.07 | -0.04 | -0.13 | 3 | 0.626 | 0.782 | 2017_vs_2018 | Middle | Functional |
| Fishing_Vulnerability | -0.475 | -4.198 | 5.680 | -0.08 | 0.22 | 0.24 | 2 | 0.465 | 0.762 | 2017_vs_2018 | Middle | Functional |
| a | -0.491 | -4.198 | 8.223 | -0.06 | -0.07 | -0.17 | 1 | 0.126 | 0.792 | 2017_vs_2018 | Middle | Functional |
| b | -0.678 | -4.198 | 11.558 | -0.06 | 0.24 | 0.39 | 6 | 0.234 | 0.792 | 2017_vs_2018 | Middle | Functional |
| Trophic_level | -1.570 | -4.198 | 24.727 | -0.06 | -0.48 | -0.50 | 5 | 0.348 | 0.782 | 2017_vs_2018 | Middle | Functional |
| Trophic_level | 7.831 | 20.252 | 58.772 | 0.13 | -0.50 | 0.08 | 6 | 0.799 | 0.069 | 2018_vs_2019 | Middle | Functional |
| a | 3.335 | 20.252 | 29.521 | 0.11 | -0.17 | -0.27 | 2 | 0.551 | 0.178 | 2018_vs_2019 | Middle | Functional |
| b | 3.181 | 20.252 | 18.074 | 0.18 | 0.39 | 0.15 | 3 | 0.708 | 0.010 | 2018_vs_2019 | Middle | Functional |
| tm | 1.833 | 20.252 | 14.805 | 0.12 | -0.13 | 0.23 | 7 | 0.956 | 0.208 | 2018_vs_2019 | Middle | Functional |
| MaxSizeTL | 1.692 | 20.252 | 19.920 | 0.08 | 0.05 | 0.30 | 4 | 0.387 | 0.257 | 2018_vs_2019 | Middle | Functional |
| FecundityMax | 1.496 | 20.252 | 7.079 | 0.21 | 0.03 | 0.29 | 5 | 1.000 | 0.010 | 2018_vs_2019 | Middle | Functional |
| Fishing_Vulnerability | 0.883 | 20.252 | 16.322 | 0.05 | 0.24 | 0.66 | 1 | 0.883 | 0.257 | 2018_vs_2019 | Middle | Functional |
| FecundityMax | 0.243 | 1.255 | 0.311 | 0.78 | 0.29 | 0.33 | 3 | 1.000 | 0.129 | 2019_vs_2020 | Middle | Functional |
| Trophic_level | 0.197 | 1.255 | 0.677 | 0.29 | 0.08 | 0.09 | 5 | 0.635 | 0.178 | 2019_vs_2020 | Middle | Functional |
| MaxSizeTL | 0.195 | 1.255 | 0.557 | 0.35 | 0.30 | 0.27 | 7 | 0.193 | 0.307 | 2019_vs_2020 | Middle | Functional |
| Fishing_Vulnerability | 0.162 | 1.255 | 0.417 | 0.39 | 0.66 | 0.63 | 2 | 0.762 | 0.198 | 2019_vs_2020 | Middle | Functional |
| a | 0.159 | 1.255 | 0.730 | 0.22 | -0.27 | -0.15 | 4 | 0.350 | 0.168 | 2019_vs_2020 | Middle | Functional |
| tm | 0.159 | 1.255 | 0.319 | 0.50 | 0.23 | 0.14 | 6 | 0.888 | 0.050 | 2019_vs_2020 | Middle | Functional |
| b | 0.140 | 1.255 | 0.272 | 0.52 | 0.15 | 0.12 | 1 | 0.506 | 0.010 | 2019_vs_2020 | Middle | Functional |
| FecundityMax | 0.396 | 1.928 | 2.205 | 0.18 | 0.33 | 0.02 | 3 | 1.000 | 0.040 | 2020_vs_2022 | Middle | Functional |
| tm | 0.355 | 1.928 | 1.832 | 0.19 | 0.14 | -0.03 | 5 | 0.944 | 0.010 | 2020_vs_2022 | Middle | Functional |
| a | 0.321 | 1.928 | 1.480 | 0.22 | -0.15 | 0.31 | 6 | 0.390 | 0.089 | 2020_vs_2022 | Middle | Functional |
| Trophic_level | 0.275 | 1.928 | 1.139 | 0.24 | 0.09 | 0.01 | 4 | 0.699 | 0.099 | 2020_vs_2022 | Middle | Functional |
| MaxSizeTL | 0.270 | 1.928 | 0.905 | 0.30 | 0.27 | 0.17 | 7 | 0.205 | 0.178 | 2020_vs_2022 | Middle | Functional |
| Fishing_Vulnerability | 0.202 | 1.928 | 0.482 | 0.42 | 0.63 | 0.40 | 1 | 0.839 | 0.178 | 2020_vs_2022 | Middle | Functional |
| b | 0.108 | 1.928 | 0.603 | 0.18 | 0.12 | -0.13 | 2 | 0.556 | 0.139 | 2020_vs_2022 | Middle | Functional |
| tm | 0.003 | -0.836 | 1.060 | 0.00 | -0.03 | 0.02 | 3 | 0.712 | 0.396 | 2022_vs_2023 | Middle | Functional |
| MaxSizeTL | -0.007 | -0.836 | 1.207 | -0.01 | 0.17 | -0.02 | 6 | -0.003 | 0.396 | 2022_vs_2023 | Middle | Functional |
| Fishing_Vulnerability | -0.017 | -0.836 | 1.685 | -0.01 | 0.40 | 0.21 | 4 | 0.439 | 0.376 | 2022_vs_2023 | Middle | Functional |
| FecundityMax | -0.172 | -0.836 | 1.783 | -0.10 | 0.02 | -0.21 | 2 | 1.000 | 0.446 | 2022_vs_2023 | Middle | Functional |
| Trophic_level | -0.174 | -0.836 | 2.917 | -0.06 | 0.01 | -0.59 | 7 | 0.231 | 0.416 | 2022_vs_2023 | Middle | Functional |
| b | -0.228 | -0.836 | 1.998 | -0.11 | -0.13 | 0.21 | 5 | 0.025 | 0.386 | 2022_vs_2023 | Middle | Functional |
| a | -0.241 | -0.836 | 1.420 | -0.17 | 0.31 | 0.04 | 1 | 0.005 | 0.515 | 2022_vs_2023 | Middle | Functional |
| MaxSizeTL | 1.077 | 4.443 | 12.061 | 0.09 | -0.02 | 0.14 | 1 | 0.242 | 0.079 | 2023_vs_2024 | Middle | Functional |
| b | 0.936 | 4.443 | 13.289 | 0.07 | 0.21 | -0.03 | 6 | 0.617 | 0.079 | 2023_vs_2024 | Middle | Functional |
| tm | 0.730 | 4.443 | 8.728 | 0.08 | 0.02 | 0.08 | 5 | 0.953 | 0.050 | 2023_vs_2024 | Middle | Functional |
| Trophic_level | 0.698 | 4.443 | 7.636 | 0.09 | -0.59 | -0.11 | 4 | 0.774 | 0.317 | 2023_vs_2024 | Middle | Functional |
| a | 0.481 | 4.443 | 4.018 | 0.12 | 0.04 | 0.02 | 3 | 0.453 | 0.050 | 2023_vs_2024 | Middle | Functional |
| Fishing_Vulnerability | 0.314 | 4.443 | 2.828 | 0.11 | 0.21 | 0.41 | 2 | 0.883 | 0.208 | 2023_vs_2024 | Middle | Functional |
| FecundityMax | 0.207 | 4.443 | 2.173 | 0.10 | -0.21 | -0.12 | 7 | 1.000 | 0.673 | 2023_vs_2024 | Middle | Functional |
| FecundityMax | 0.254 | 1.095 | 0.196 | 1.29 | 0.68 | 0.45 | 1 | 1.000 | 0.158 | 2017_vs_2018 | South | Functional |
| tm | 0.205 | 1.095 | 0.191 | 1.07 | -0.06 | 0.33 | 5 | 0.934 | 0.129 | 2017_vs_2018 | South | Functional |
| b | 0.181 | 1.095 | 0.203 | 0.89 | 0.17 | -0.11 | 3 | 0.585 | 0.089 | 2017_vs_2018 | South | Functional |
| Trophic_level | 0.164 | 1.095 | 0.199 | 0.82 | -0.04 | 0.15 | 4 | 0.735 | 0.069 | 2017_vs_2018 | South | Functional |
| a | 0.132 | 1.095 | 0.148 | 0.89 | -0.06 | -0.16 | 6 | 0.419 | 0.564 | 2017_vs_2018 | South | Functional |
| Fishing_Vulnerability | 0.086 | 1.095 | 0.076 | 1.12 | 0.65 | 0.65 | 2 | 0.856 | 0.673 | 2017_vs_2018 | South | Functional |
| MaxSizeTL | 0.072 | 1.095 | 0.077 | 0.93 | 0.18 | 0.23 | 7 | 0.232 | 0.564 | 2017_vs_2018 | South | Functional |
| b | 1.973 | 10.081 | 23.682 | 0.08 | -0.11 | 0.34 | 7 | 0.547 | 0.010 | 2018_vs_2019 | South | Functional |
| Fishing_Vulnerability | 1.852 | 10.081 | 22.444 | 0.08 | 0.65 | 0.32 | 2 | 0.790 | 0.010 | 2018_vs_2019 | South | Functional |
| FecundityMax | 1.690 | 10.081 | 18.638 | 0.09 | 0.45 | -0.02 | 4 | 1.000 | 0.020 | 2018_vs_2019 | South | Functional |
| tm | 1.250 | 10.081 | 10.808 | 0.12 | 0.33 | -0.00 | 1 | 0.901 | 0.010 | 2018_vs_2019 | South | Functional |
| a | 1.200 | 10.081 | 12.942 | 0.09 | -0.16 | -0.11 | 5 | 0.379 | 0.010 | 2018_vs_2019 | South | Functional |
| MaxSizeTL | 1.122 | 10.081 | 12.479 | 0.09 | 0.23 | 0.03 | 3 | 0.196 | 0.010 | 2018_vs_2019 | South | Functional |
| Trophic_level | 0.994 | 10.081 | 7.364 | 0.13 | 0.15 | -0.26 | 6 | 0.671 | 0.010 | 2018_vs_2019 | South | Functional |
| Trophic_level | 0.701 | 2.755 | 4.105 | 0.17 | -0.26 | 0.12 | 1 | 0.708 | 0.020 | 2019_vs_2020 | South | Functional |
| FecundityMax | 0.507 | 2.755 | 4.318 | 0.12 | -0.02 | 0.30 | 6 | 1.000 | 0.040 | 2019_vs_2020 | South | Functional |
| Fishing_Vulnerability | 0.389 | 2.755 | 10.463 | 0.04 | 0.32 | 0.72 | 3 | 0.811 | 0.178 | 2019_vs_2020 | South | Functional |
| MaxSizeTL | 0.355 | 2.755 | 7.378 | 0.05 | 0.03 | 0.26 | 4 | 0.254 | 0.099 | 2019_vs_2020 | South | Functional |
| b | 0.283 | 2.755 | 6.306 | 0.04 | 0.34 | 0.01 | 5 | 0.580 | 0.129 | 2019_vs_2020 | South | Functional |
| a | 0.266 | 2.755 | 3.064 | 0.09 | -0.11 | -0.13 | 7 | 0.438 | 0.030 | 2019_vs_2020 | South | Functional |
| tm | 0.254 | 2.755 | 7.115 | 0.04 | -0.00 | 0.29 | 2 | 0.908 | 0.238 | 2019_vs_2020 | South | Functional |
| FecundityMax | 0.201 | 0.719 | 0.138 | 1.46 | 0.30 | 0.74 | 5 | 1.000 | 0.059 | 2020_vs_2021 | South | Functional |
| tm | 0.124 | 0.719 | 0.109 | 1.14 | 0.29 | 0.16 | 4 | 0.905 | 0.782 | 2020_vs_2021 | South | Functional |
| MaxSizeTL | 0.092 | 0.719 | 0.092 | 1.00 | 0.26 | 0.38 | 7 | 0.279 | 0.891 | 2020_vs_2021 | South | Functional |
| a | 0.091 | 0.719 | 0.079 | 1.15 | -0.13 | -0.33 | 6 | 0.452 | 0.525 | 2020_vs_2021 | South | Functional |
| b | 0.072 | 0.719 | 0.071 | 1.01 | 0.01 | 0.16 | 1 | 0.580 | 0.396 | 2020_vs_2021 | South | Functional |
| Trophic_level | 0.070 | 0.719 | 0.049 | 1.44 | 0.12 | 0.21 | 2 | 0.707 | 0.911 | 2020_vs_2021 | South | Functional |
| Fishing_Vulnerability | 0.069 | 0.719 | 0.050 | 1.36 | 0.72 | 0.83 | 3 | 0.807 | 0.653 | 2020_vs_2021 | South | Functional |
| a | 0.213 | 0.840 | 1.610 | 0.13 | -0.33 | 0.18 | 3 | 0.440 | 0.356 | 2021_vs_2022 | South | Functional |
| b | 0.156 | 0.840 | 1.073 | 0.15 | 0.16 | -0.05 | 7 | 0.615 | 0.406 | 2021_vs_2022 | South | Functional |
| FecundityMax | 0.147 | 0.840 | 2.279 | 0.06 | 0.74 | 0.22 | 6 | 1.000 | 0.396 | 2021_vs_2022 | South | Functional |
| Trophic_level | 0.124 | 0.840 | 1.121 | 0.11 | 0.21 | 0.01 | 4 | 0.762 | 0.218 | 2021_vs_2022 | South | Functional |
| MaxSizeTL | 0.106 | 0.840 | 1.367 | 0.08 | 0.38 | 0.04 | 2 | 0.254 | 0.406 | 2021_vs_2022 | South | Functional |
| Fishing_Vulnerability | 0.048 | 0.840 | 2.455 | 0.02 | 0.83 | 0.37 | 1 | 0.889 | 0.436 | 2021_vs_2022 | South | Functional |
| tm | 0.045 | 0.840 | 1.288 | 0.04 | 0.16 | -0.06 | 5 | 0.946 | 0.396 | 2021_vs_2022 | South | Functional |
| Trophic_level | -0.331 | -9.433 | 22.942 | -0.01 | 0.01 | 0.01 | 3 | 0.367 | 0.426 | 2022_vs_2023 | South | Functional |
| tm | -0.779 | -9.433 | 31.787 | -0.02 | -0.06 | 0.01 | 5 | 0.696 | 0.743 | 2022_vs_2023 | South | Functional |
| MaxSizeTL | -1.043 | -9.433 | 42.561 | -0.02 | 0.04 | 0.13 | 4 | 0.035 | 0.624 | 2022_vs_2023 | South | Functional |
| b | -1.307 | -9.433 | 70.806 | -0.02 | -0.05 | 0.12 | 1 | 0.228 | 0.624 | 2022_vs_2023 | South | Functional |
| FecundityMax | -1.528 | -9.433 | 99.820 | -0.02 | 0.22 | 0.32 | 2 | 1.000 | 0.614 | 2022_vs_2023 | South | Functional |
| Fishing_Vulnerability | -1.574 | -9.433 | 69.161 | -0.02 | 0.37 | 0.52 | 7 | 0.529 | 0.604 | 2022_vs_2023 | South | Functional |
| a | -2.871 | -9.433 | 167.062 | -0.02 | 0.18 | -0.09 | 6 | 0.118 | 0.673 | 2022_vs_2023 | South | Functional |
| FecundityMax | 0.267 | 0.074 | 2.885 | 0.09 | 0.32 | 0.38 | 2 | 1.000 | 0.287 | 2023_vs_2024 | South | Functional |
| Fishing_Vulnerability | 0.027 | 0.074 | 3.986 | 0.01 | 0.52 | 0.62 | 6 | 3.951 | 0.485 | 2023_vs_2024 | South | Functional |
| MaxSizeTL | 0.017 | 0.074 | 4.201 | 0.00 | 0.13 | 0.15 | 7 | 3.596 | 0.485 | 2023_vs_2024 | South | Functional |
| Trophic_level | 0.002 | 0.074 | 5.886 | 0.00 | 0.01 | 0.06 | 4 | 4.225 | 0.485 | 2023_vs_2024 | South | Functional |
| tm | -0.020 | 0.074 | 4.017 | -0.01 | 0.01 | -0.01 | 3 | 3.200 | 0.931 | 2023_vs_2024 | South | Functional |
| b | -0.056 | 0.074 | 5.150 | -0.01 | 0.12 | 0.16 | 1 | 4.194 | 0.485 | 2023_vs_2024 | South | Functional |
| a | -0.163 | 0.074 | 9.343 | -0.02 | -0.09 | -0.05 | 5 | 3.963 | 0.485 | 2023_vs_2024 | South | Functional |

**Table S8 | Temporal trends in biomass of SIMPER-identified top species**

Linear trend analyses (biomass ~ Year) for the five most influential species identified by SIMPER in each subregion. Slope represents the annual change in biomass (g m⁻² yr⁻¹); R² indicates the proportion of variance explained by Year. Trend categories use p < 0.05 and slope sign.

| **Subregion** | **Species** | **Slope** | **p** | **R²** | **Trend** |
| --- | --- | --- | --- | --- | --- |
| North | *Chromis punctipinnis* | 2.05 | 0.051 | 0.441 | Increasing (ns) |
| North | *Embiotoca jacksoni* | 0.64 | 0.527 | 0.060 | Increasing (ns) |
| North | *Hypsypops rubicundus* | 0.62 | 0.390 | 0.107 | Increasing (ns) |
| North | *Paralabrax clathratus* | 0.76 | 0.203 | 0.220 | Increasing (ns) |
| North | *BodianusSemicossyphus pulcher* | -0.83 | 0.583 | 0.045 | Decreasing (ns) |
| Middle | *Chromis punctipinnis* | 2.34 | 0.107 | 0.434 | Increasing (ns) |
| Middle | *Girella nigricans* | 1.59 | 0.114 | 0.505 | Increasing (ns) |
| Middle | *Hypsypops rubicundus* | 1.19 | 0.328 | 0.190 | Increasing (ns) |
| Middle | *Paralabrax clathratus* | 0.17 | 0.971 | 0.000 | Increasing (ns) |
| Middle | *BodianusSemicossyphus pulcher* | -0.94 | 0.929 | 0.002 | Decreasing (ns) |
| South | *Hypsypops rubicundus* | -1.66 | 0.128 | 0.298 | Decreasing (ns) |
| South | *Paralabrax clathratus* | -6.83 | 0.147 | 0.275 | Decreasing (ns) |
| South | *BodianusSemicossyphus pulcher* | -4.21 | 0.174 | 0.246 | Decreasing (ns) |
| South | *Sphyraena argentea* | NA | NA | 0 | No trend |
| South | *Zapteryx exasperata* | -25.95 | 0.018 | 0.786 | Decreasing (significant) |

**Table S9 | GLMM results for biomass-weighted fish size**

Generalised linear mixed-effects model for log-transformed biomass-weighted mean fish size at the transect level. Fixed effects: Subregion (North = reference), Year (scaled), functional group (Herbivore = reference), and their interactions. Site and Sub-site (nested within Site) were included as random intercepts. Significance codes: *** p < 0.001, ** p < 0.01, * p < 0.05, . p < 0.1.

| **Term** | **Estimate** | **SE** | **CI low** | **CI high** | **p** |  |
| --- | --- | --- | --- | --- | --- | --- |
| (Intercept) | 3.168 | 0.056 | 3.059 | 3.278 | <0.001 | *** |
| Middle vs North | 0.030 | 0.087 | -0.141 | 0.201 | 0.731 |  |
| South vs North | -0.085 | 0.081 | -0.245 | 0.075 | 0.296 |  |
| Year | 0.081 | 0.068 | -0.052 | 0.214 | 0.232 |  |
| Macroinvertivore | 0.112 | 0.054 | 0.007 | 0.218 | 0.037 | * |
| Microinvertivore | -0.277 | 0.057 | -0.389 | -0.165 | <0.001 | *** |
| Piscivore | 0.045 | 0.055 | -0.063 | 0.153 | 0.416 |  |
| Planktivore | -0.629 | 0.055 | -0.737 | -0.521 | <0.001 | *** |
| Middle × Year | -0.011 | 0.084 | -0.176 | 0.155 | 0.898 |  |
| South × Year | -0.164 | 0.085 | -0.330 | 0.003 | 0.054 |  |
| Middle × Macroinvertivore | 0.146 | 0.085 | -0.020 | 0.312 | 0.084 |  |
| South × Macroinvertivore | 0.241 | 0.074 | 0.096 | 0.387 | 0.001 | ** |
| Middle × Microinvertivore | 0.063 | 0.088 | -0.110 | 0.236 | 0.475 |  |
| South × Microinvertivore | 0.048 | 0.077 | -0.103 | 0.199 | 0.537 |  |
| Middle × Piscivore | 0.165 | 0.088 | -0.007 | 0.337 | 0.061 |  |
| South × Piscivore | 0.229 | 0.076 | 0.081 | 0.377 | 0.002 | ** |
| Middle × Planktivore | 0.039 | 0.086 | -0.130 | 0.209 | 0.648 |  |
| South × Planktivore | -0.006 | 0.078 | -0.158 | 0.147 | 0.942 |  |
| Year × Macroinvertivore | -0.129 | 0.068 | -0.262 | 0.004 | 0.057 |  |
| Year × Microinvertivore | -0.054 | 0.070 | -0.192 | 0.084 | 0.446 |  |
| Year × Piscivore | -0.043 | 0.069 | -0.179 | 0.092 | 0.531 |  |
| Year × Planktivore | -0.144 | 0.070 | -0.280 | -0.008 | 0.038 | * |
| Middle × Year × Macroinvertivore | 0.096 | 0.086 | -0.072 | 0.263 | 0.263 |  |
| South × Year × Macroinvertivore | 0.144 | 0.086 | -0.024 | 0.313 | 0.093 |  |
| Middle × Year × Microinvertivore | 0.092 | 0.088 | -0.081 | 0.265 | 0.298 |  |
| South × Year × Microinvertivore | 0.103 | 0.088 | -0.070 | 0.276 | 0.243 |  |
| Middle × Year × Piscivore | 0.087 | 0.088 | -0.085 | 0.260 | 0.321 |  |
| South × Year × Piscivore | 0.133 | 0.087 | -0.038 | 0.304 | 0.128 |  |
| Middle × Year × Planktivore | 0.078 | 0.087 | -0.092 | 0.249 | 0.369 |  |
| South × Year × Planktivore | 0.227 | 0.090 | 0.052 | 0.403 | 0.011 | * |

**Table S10 | Pairwise comparisons of biomass-weighted fish size among subregions**

Pairwise comparisons of estimated marginal means (from Table S9) among subregions within each functional group, with Tukey adjustment for multiple testing. Estimates and SE on the log scale.

| **Contrast** | **Functional group** | **Estimate** | **SE** | **df** | **t** | **p** |
| --- | --- | --- | --- | --- | --- | --- |
| North - Middle | Herbivore | -0.030 | 0.087 | 1909 | -0.34 | 0.937 |
| North - South | Herbivore | 0.085 | 0.081 | 1909 | 1.04 | 0.549 |
| Middle - South | Herbivore | 0.115 | 0.089 | 1909 | 1.29 | 0.402 |
| North - Middle | Macroinvertivore | -0.176 | 0.068 | 1909 | -2.60 | 0.025 |
| North - South | Macroinvertivore | -0.156 | 0.060 | 1909 | -2.61 | 0.025 |
| Middle - South | Macroinvertivore | 0.020 | 0.072 | 1909 | 0.28 | 0.958 |
| North - Middle | Microinvertivore | -0.093 | 0.073 | 1909 | -1.29 | 0.404 |
| North - South | Microinvertivore | 0.038 | 0.063 | 1909 | 0.60 | 0.822 |
| Middle - South | Microinvertivore | 0.131 | 0.074 | 1909 | 1.76 | 0.185 |
| North - Middle | Piscivore | -0.195 | 0.072 | 1909 | -2.70 | 0.019 |
| North - South | Piscivore | -0.144 | 0.062 | 1909 | -2.33 | 0.052 |
| Middle - South | Piscivore | 0.051 | 0.076 | 1909 | 0.67 | 0.780 |
| North - Middle | Planktivore | -0.069 | 0.070 | 1909 | -0.99 | 0.584 |
| North - South | Planktivore | 0.091 | 0.064 | 1909 | 1.41 | 0.335 |
| Middle - South | Planktivore | 0.160 | 0.076 | 1909 | 2.11 | 0.089 |

**Table S11 | Species-specific mixed-effects results for temporal trends in body size**

Species-specific GLMMs for log-transformed biomass-weighted mean size, fitted separately for each species within each subregion with Site as random intercept. Analyses restricted to species with n > 20 observations per subregion. The estimate column refers to the annual effect on the log scale; percent change is the back-transformed annual percentage change. Significance codes: *** p < 0.001, ** p < 0.01, * p < 0.05, . p < 0.1.

| **Species** | **Subregion** | **Estimate** | **% change yr⁻¹** | **SE** | **p** |  | **R²** |
| --- | --- | --- | --- | --- | --- | --- | --- |
| *Chromis punctipinnis* | North | -0.020 | -1.94 | 0.072 | 0.784 |  | 0.003 |
| *Chromis punctipinnis* | South | -0.063 | -6.13 | 0.061 | 0.302 |  | 0.051 |
| *Halichoeres semicinctus* | South | -0.095 | -9.06 | 0.044 | 0.030 | * | 0.190 |
| *Hypsypops rubicundus* | North | 0.036 | 3.66 | 0.043 | 0.400 |  | 0.028 |
| *Hypsypops rubicundus* | South | -0.062 | -6.05 | 0.035 | 0.070 |  | 0.141 |
| *Oxyjulis californica* | North | -0.020 | -1.95 | 0.060 | 0.744 |  | 0.005 |
| *Paralabrax clathratus* | North | 0.101 | 10.61 | 0.045 | 0.024 | * | 0.170 |
| *Paralabrax clathratus* | South | 0.001 | 0.11 | 0.037 | 0.977 |  | 0.000 |
| *Semicossyphus pulcher* | North | -0.018 | -1.81 | 0.067 | 0.787 |  | 0.003 |
| *Semicossyphus pulcher* | South | -0.047 | -4.62 | 0.043 | 0.271 |  | 0.057 |

**Figure S1 | Species-specific body-size trends**

Species-specific temporal trends in biomass-weighted mean body size across subregions for species with n > 20 observations per subregion. Lines are species-specific GLMM predictions (log scale); shaded areas represent 95% confidence intervals. Corresponding statistics are reported in Table S11.


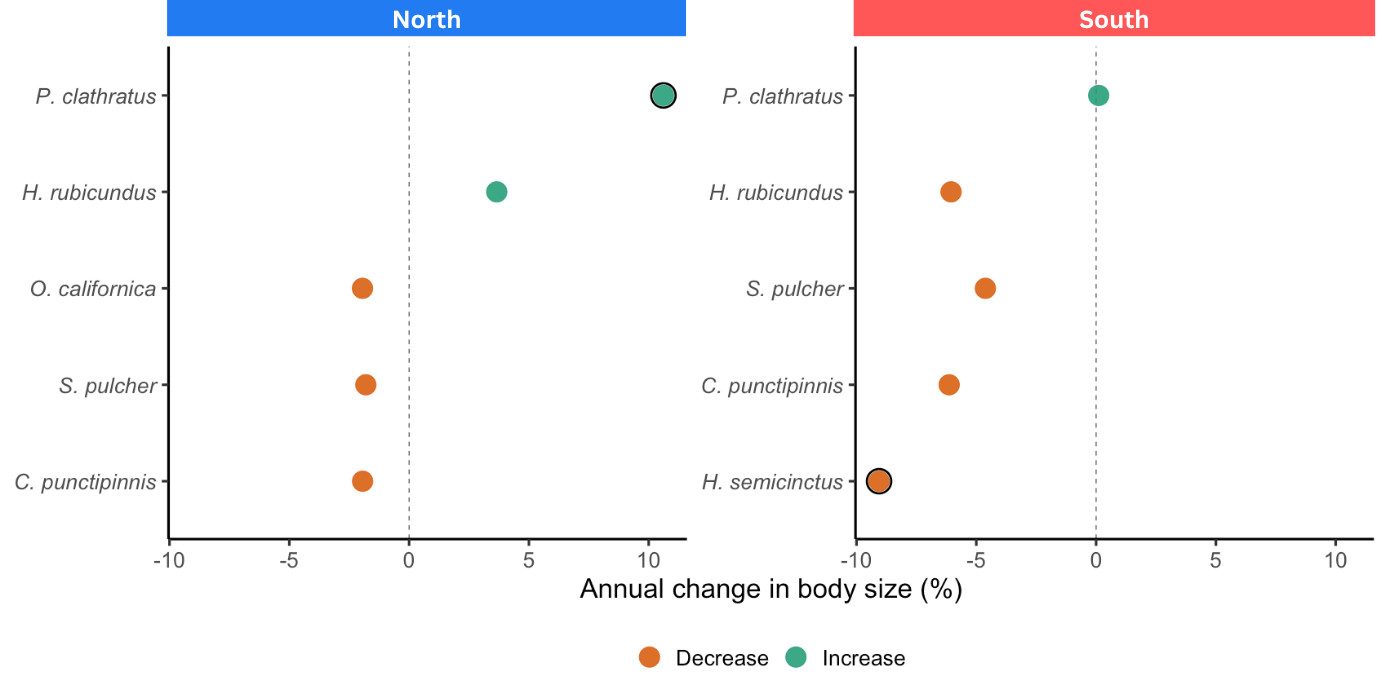


**Table S12 | GLMM results for biomass turnover (P/B)**

Generalised linear mixed-effects model for biomass turnover (P/B × 100%) at the transect level, fitted with a Gaussian error distribution after log-transformation. Fixed effects: Subregion, Year (scaled), functional group, and their interactions. Site and Sub-site (nested) were included as random intercepts. Significance codes: *** p < 0.001, ** p < 0.01, * p < 0.05.

| **Term** | **Estimate** | **SE** | **z** | **p** | **CI low** | **CI high** |  |
| --- | --- | --- | --- | --- | --- | --- | --- |
| (Intercept) | -1.865 | 0.301 | -6.20 | <0.001 | -2.454 | -1.276 | *** |
| Middle | -0.150 | 0.452 | -0.33 | 0.740 | -1.036 | 0.735 |  |
| South | -0.329 | 0.420 | -0.79 | 0.432 | -1.152 | 0.493 |  |
| Year | -0.116 | 0.387 | -0.30 | 0.765 | -0.875 | 0.643 |  |
| Macroinvertivore | -0.704 | 0.338 | -2.08 | 0.037 | -1.367 | -0.042 | * |
| Microinvertivore | -0.912 | 0.360 | -2.53 | 0.011 | -1.619 | -0.206 | * |
| Piscivore | -0.198 | 0.347 | -0.57 | 0.568 | -0.879 | 0.482 |  |
| Planktivore | -3.568 | 0.347 | -10.27 | <0.001 | -4.248 | -2.887 | *** |
| Middle × Year | 0.001 | 0.469 | 0.00 | 0.999 | -0.918 | 0.920 |  |
| South × Year | -0.148 | 0.482 | -0.31 | 0.759 | -1.093 | 0.797 |  |
| Middle × Macroinvertivore | -0.070 | 0.531 | -0.13 | 0.894 | -1.111 | 0.970 |  |
| South × Macroinvertivore | 0.103 | 0.468 | 0.22 | 0.826 | -0.814 | 1.020 |  |
| Middle × Microinvertivore | 0.924 | 0.557 | 1.66 | 0.097 | -0.168 | 2.015 |  |
| South × Microinvertivore | 0.490 | 0.485 | 1.01 | 0.312 | -0.461 | 1.441 |  |
| Middle × Piscivore | 0.063 | 0.551 | 0.11 | 0.909 | -1.018 | 1.143 |  |
| South × Piscivore | 0.413 | 0.477 | 0.87 | 0.387 | -0.522 | 1.348 |  |
| Middle × Planktivore | 1.245 | 0.544 | 2.29 | 0.022 | 0.179 | 2.310 | * |
| South × Planktivore | 0.598 | 0.490 | 1.22 | 0.223 | -0.363 | 1.558 |  |
| Year × Macroinvertivore | 0.125 | 0.425 | 0.29 | 0.768 | -0.707 | 0.957 |  |
| Year × Microinvertivore | -0.045 | 0.442 | -0.10 | 0.918 | -0.911 | 0.821 |  |
| Year × Piscivore | 0.214 | 0.434 | 0.49 | 0.621 | -0.636 | 1.064 |  |
| Year × Planktivore | 0.162 | 0.435 | 0.37 | 0.710 | -0.691 | 1.014 |  |
| Middle × Year × Macroinvertivore | -0.132 | 0.536 | -0.25 | 0.805 | -1.183 | 0.919 |  |
| South × Year × Macroinvertivore | 0.582 | 0.540 | 1.08 | 0.281 | -0.477 | 1.641 |  |
| Middle × Year × Microinvertivore | 0.012 | 0.555 | 0.02 | 0.983 | -1.077 | 1.101 |  |
| South × Year × Microinvertivore | 0.558 | 0.554 | 1.01 | 0.314 | -0.529 | 1.644 |  |
| Middle × Year × Piscivore | -0.218 | 0.552 | -0.39 | 0.693 | -1.300 | 0.864 |  |
| South × Year × Piscivore | 0.047 | 0.548 | 0.09 | 0.932 | -1.027 | 1.121 |  |
| Middle × Year × Planktivore | -0.353 | 0.546 | -0.65 | 0.518 | -1.423 | 0.717 |  |
| South × Year × Planktivore | 0.410 | 0.563 | 0.73 | 0.466 | -0.692 | 1.513 |  |

**SIMPER analysis**

To identify the main drivers of temporal shifts in β-diversity, we summarised SIMPER results across consecutive year-pair comparisons within each subregion. Table S13 (taxonomic) and Table S14 (functional) summarise the strongest SIMPER contributors across subregions. Results are presented separately for taxonomic (species-level) and functional (trait-based) components, focusing on contributors with significant effects (p < 0.05).

**Taxonomic drivers of temporal dissimilarity**

Temporal changes in community composition were driven by a limited number of species (Table S13), with regional differences in the consistency and strength of these contributions. In the North, several species contributed significantly to temporal dissimilarity, but their contributions were generally infrequent and distributed across taxa. The most consistent contributors were *Paralabrax clathratus* and *Chromis punctipinnis*, each significant in two year-pair comparisons, while *Hypsypops rubicundus* contributed significantly once. Other high-ranking species, including Bodianus pulcher and Rhacochilus vacca, did not contribute significantly in any comparison. Overall, taxonomic change in the North was characterised by variable and non-repeated species contributions. In the Middle, taxonomic signals were weaker and more restricted. Only a few species contributed significantly, with *Embiotoca jacksoni* showing the strongest and most consistent pattern (significant in three year-pair comparisons). *Hypsypops rubicundus*, *Halichoeres semicinctus*, and *Paralabrax clathratus* each contributed significantly in a single comparison. Most high-ranking species did not show significant contributions, indicating that temporal dissimilarity in this subregion was driven by a small subset of species rather than the dominant taxa. In the South, taxonomic contributions were both stronger and more consistent. Several species contributed significantly across multiple year-pair comparisons, including *Paralabrax clathratus* and *Anisotremus davidsonii* (each significant in three comparisons), as well as *Zapteryx exasperata*, *Urobatis concentricus*, and *Paralabrax nebulifer* (each significant in two comparisons). Additional species contributed significantly in single comparisons. This indicates that temporal community reorganisation in the South was driven by a broader set of taxa with repeated contributions over time.

**Table S13 | Major taxonomic contributors to temporal community change (SIMPER)**

Mean contribution represents the average contribution of each species to Bray–Curtis dissimilarity across all consecutive year-pair comparisons within a subregion. n sig indicates the number of comparisons in which the species was a significant SIMPER contributor (permutation p < 0.05, 999 permutations). Entries within each subregion are sorted by mean contribution from highest to lowest.

| **Subregion** | **Species** | **Mean contribution** | **n sig** |
| --- | --- | --- | --- |
| North | *Bodianus pulcher* | 0.150 | 0 |
| North | *Hypsypops rubicundus* | 0.120 | 1 |
| North | *Paralabrax clathratus* | 0.110 | 2 |
| North | *Chromis punctipinnis* | 0.090 | 2 |
| North | *Rhacochilus vacca* | 0.070 | 0 |
| Middle | *Bodianus pulcher* | 0.240 | 0 |
| Middle | *Paralabrax clathratus* | 0.130 | 1 |
| Middle | *Rhacochilus vacca* | 0.080 | 0 |
| Middle | *Chromis punctipinnis* | 0.070 | 0 |
| Middle | *Girella nigricans* | 0.060 | 0 |
| South | *Paralabrax clathratus* | 0.180 | 3 |
| South | *Bodianus pulcher* | 0.170 | 0 |
| South | *Zapteryx exasperata* | 0.090 | 2 |
| South | *Hypsypops rubicundus* | 0.060 | 1 |
| South | *Halichoeres semicinctus* | 0.050 | 1 |

**Functional drivers of temporal dissimilarity**

Patterns of functional change (Table S14) differed among subregions. In the North, functional change was driven by several traits, with a (growth coefficient) and Fishing_Vulnerability showing the strongest signals. Fishing_Vulnerability contributed significantly in four year-pair comparisons, and a and b were each significant in one and two comparisons respectively, while FecundityMax and Trophic_level showed no significant contributions. This indicates that temporal functional change in the North was primarily associated with shifts in growth parameters and vulnerability to fishing pressure rather than trophic structure or reproductive traits. In the Middle, functional change was most strongly associated with trophic and life-history traits. Trophic_level showed the largest mean contribution and was significant in two year-pair comparisons, while a (growth coefficient), Fishing_Vulnerability, and MaxSizeTL each contributed significantly once. FecundityMax had a moderate mean contribution but was not significant in any comparison. These results suggest that temporal functional change in the Middle was driven by coordinated shifts across trophic position, growth, and body size dimensions. In the South, multiple traits contributed significantly, indicating a multidimensional pattern of functional change. FecundityMax showed the strongest and most consistent signal, contributing significantly in four year-pair comparisons. Trophic_level was significant in one comparison, while tm and Fishing_Vulnerability each contributed significantly once. a showed a comparable mean contribution to Trophic_level but was not significant in any comparison. Overall, functional change in the South was characterised by coordinated shifts across reproductive output, trophic position, and vulnerability, suggesting a broader restructuring of functional community composition over time.

**Table S14 | Major functional contributors to temporal community change (SIMPER)**

Mean contribution represents the average contribution of each trait to Bray–Curtis dissimilarity across all consecutive year-pair comparisons within a subregion. n sig indicates the number of comparisons in which the trait was a significant SIMPER contributor (permutation p < 0.05, 999 permutations). Traits: a and b, length–weight coefficients; FecundityMax, maximum fecundity; Fishing_Vulnerability, fishing vulnerability; MaxSizeTL, maximum total length; tm, age at maturity; Trophic_level, trophic level. Entries within each subregion are sorted by mean contribution from highest to lowest.

| **Subregion** | **Trait** | **Mean contribution** | **n sig** |
| --- | --- | --- | --- |
| North | a | 0.031 | 1 |
| North | FecundityMax | 0.030 | 0 |
| North | Fishing_Vulnerability | 0.027 | 4 |
| North | Trophic_level | 0.025 | 0 |
| North | b | 0.020 | 2 |
| Middle | Trophic_level | 0.031 | 2 |
| Middle | FecundityMax | 0.026 | 0 |
| Middle | a | 0.019 | 1 |
| Middle | Fishing_Vulnerability | 0.017 | 1 |
| Middle | MaxSizeTL | 0.014 | 1 |
| South | FecundityMax | 0.036 | 4 |
| South | Trophic_level | 0.021 | 1 |
| South | a | 0.020 | 0 |
| South | tm | 0.018 | 1 |
| South | Fishing_Vulnerability | 0.017 | 1 |

**Clustering of SIMPER contribution patterns**

Hierarchical clustering of significant SIMPER contributors revealed clear differences in the structure of temporal contribution patterns among subregions. At the taxonomic level, clustering in the North showed relatively high dissimilarity among species, indicating that significant contributions occurred at different times and did not form consistent groups. The Middle exhibited a simplified structure, with only two species contributing significantly and merging at high clustering distances, reflecting non-overlapping temporal contributions. In contrast, the South showed a more structured clustering pattern, with several species forming tight clusters at low dissimilarity, indicating shared temporal contribution patterns, while key taxa such as *P. clathratus* and *Z. exasperata* remained more distinct.

At the functional level, clustering in the North reflected contributions concentrated in fishing vulnerability and growth parameters, with traits merging at varying dissimilarity levels consistent with partially shared but not fully synchronised temporal dynamics. In the Middle, trophic level and the growth coefficient a showed distinct contribution patterns, while fishing vulnerability and maximum body size formed a separate group, and these clusters merged only at high dissimilarity, reflecting limited coherence among functional contributors. In the South, functional clustering was more pronounced, with reproductive traits including maximum fecundity and age at maturity forming one group, and trophic position and fishing vulnerability showing partially overlapping temporal dynamics, consistent with the broader multidimensional reorganisation observed in that subregion.

Comparison of SIMPER-based trait dissimilarity with independent trait-space dissimilarity based on Gower distance and principal coordinates analysis revealed a statistically significant but weak correlation between the two approaches (Mantel r = 0.014, p = 0.001). This indicates that while the trait profiles captured a consistent and non-random signal of functional community structure, SIMPER-based functional patterns primarily reflect biomass-weighted changes in community composition rather than overall similarity in trait space, and the two representations are largely complementary rather than redundant.

**Figure S2 | SIMPER clustering dendrograms**

Hierarchical clustering dendrograms of significant SIMPER contributors for each subregion, based on Euclidean distance and complete linkage applied to species-by-comparison (A: taxonomic) and trait-by-comparison (B: functional) contribution matrices. Clusters reflect similarity in temporal contribution patterns rather than ecological similarity in trait space.


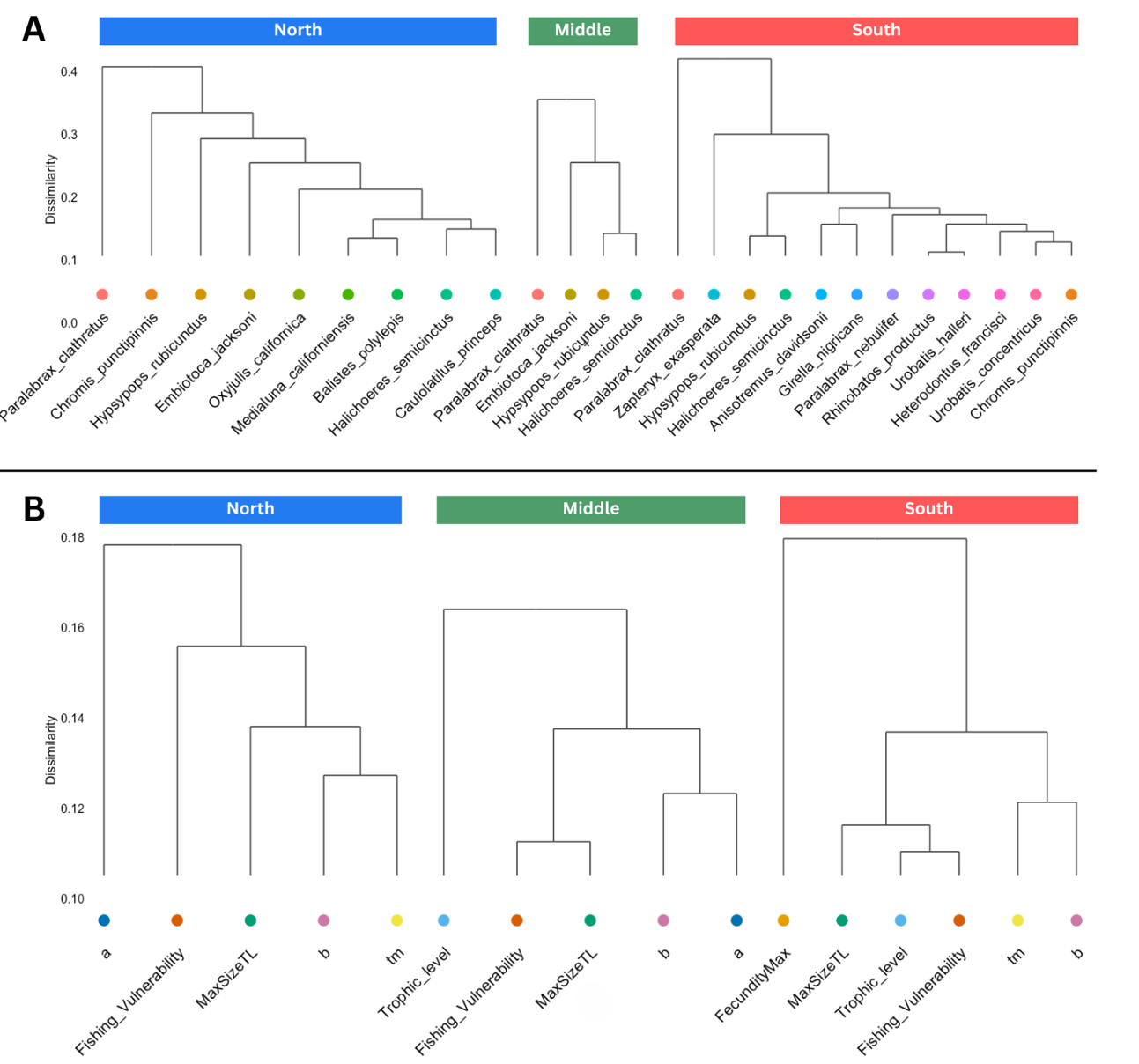

Supplement: Supplemental Information 3 [file peerj-14-21452-s003.docx]
